# Maternal psychiatric disorders before, during, and after pregnancy: a national cohort study in Sweden

**DOI:** 10.1038/s41380-025-03212-9

**Published:** 2025-09-25

**Authors:** Emma Bränn, Jerry Guintivano, Yihui Yang, Louise Lundborg, Marion Opatowski, Fang Fang, Unnur A. Valdimarsdóttir, Emma Fransson, Alkistis Skalkidou, Yi Lu, Donghao Lu

**Affiliations:** 1 Institute of Environmental Medicine, Karolinska Institutet, Stockholm, Sweden; 2 Centre for Epidemiology and Community Medicine, Region Stockholm, Stockholm, Sweden; 3 Department of Psychiatry, University of North Carolina at Chapel Hill, Chapel Hill, NC USA; 4 Clinical Epidemiology Division, Department of Medicine, Karolinska Institutet, Stockholm, Sweden; 5 Center of Public Health Sciences, Faculty of Medicine, University of Iceland, Reykjavík, Iceland; 6 Department of Epidemiology, Harvard TH Chan School of Public Health, Harvard University, Boston, MA USA; 7 Department of Women´s and Children´s health, Uppsala University, Uppsala, Sweden; 8 Centre for Translational Microbiome Research, Department of Microbiology, Tumor and Cell Biology, Karolinska Institutet, Stockholm, Sweden; 9 Department of Medical Epidemiology and Biostatistics, Karolinska Institutet, Stockholm, Sweden

**Keywords:** Psychiatric disorders, Diseases

## Abstract

Maternal mental health is a critical public health issue, yet the evidence on rates of incident psychiatric disorders before, during, and after pregnancy is limited. This study aimed to describe the calendar time trends and characterize and compare the risk of maternal psychiatric disorders before, during, and after pregnancy. Leveraging the national and regional registers in Sweden, we conducted a cohort study of all women who gave birth 2003–2019 in Sweden (1,799,010 pregnancies from 1,052,977 women). We identified any incident diagnosis of psychiatric disorders recorded during three periods: the preconceptional year, pregnancy, and the postpartum year. We calculated age and calendar year standardized incidence rate (SIR) of psychiatric disorders annually, and by week across three periods. We further estimated the incidence rate ratio (IRR) using the rate during corresponding preconceptional weeks as the reference. The SIR of maternal psychiatric disorder overall increased from 2003–2019, especially for preconceptional disorders. During the preconceptional year the weekly SIR of any psychiatric disorder was stable at around 25 per 1000 person-years. The SIR gradually decreased during pregnancy to a minimum of 4 per 1000 person-years and bounced back to the preconceptional levels during the postpartum year. This trend was similar in all subtypes of psychiatric disorders, except for depression and psychosis for which an increase was noted at 5–15 and 0–20 postpartum weeks, respectively. An increased incidence rate of maternal psychiatric disorder diagnosed before, during, and after pregnancy was found over time. Our findings suggest an increased risk of depression and psychosis shortly after delivery, although a lowered risk of other psychiatric disorders during and after pregnancy, compared to before pregnancy.

## Introduction

Maternal mental ill-health negatively affects short-term physical health and has lasting consequences on the mother’s psychological well-being [1]. Moreover, our recent studies have shown long-term negative effects on mothers [2]. For example, depression during or after pregnancy has been associated with increased risks of maternal autoimmune disorders [3], cardiovascular disorders [4], premenstrual disorders [5], suicidal behavior [6], and premature death [7]. Hence, it is of paramount importance to address maternal mental ill-health through early detection and intervention.

The entire pregnancy and postpartum period encompasses significant alterations in body function, hormonal levels [8], and inflammatory responses [9]. It also comprises a life changing event, including adaptation to a new family constellation [10]. The dynamic changes and interactions between these biological and psychosocial factors may predispose mothers to mental ill-health at different rates from pregnancy to postpartum. Understanding the risk of psychiatric disorders during this critical time window may help allocate clinical resources effectively.

Many studies have shown a higher prevalence of depressive episodes in women during pregnancy and postpartum [11–13] compared to the general population. The general population includes both birthing and non-birthing women and may differ in risk of psychiatric disorders [14, 15]. A few studies have described mental health before [16, 17] and after [18] pregnancy in birthing women. Moreover, several studies compared mental health before, and/or during and after to a non-birthing population [19, 20]. To our knowledge, only one Danish study evaluated the incidence rate of specialist-diagnosed depression [21] and one Australian study assessed rates of hospitalized depression [22] before, during, and after pregnancy within a birthing population. These studies described a higher rate of depression after pregnancy than before pregnancy, although conflicting results were observed for during pregnancy. However, these studies did not capture patients attended in primary care. Moreover, no formal statistical comparison was carried out yielding conclusions based on visual assessments only, which can be subject to random errors, and all pregnancies were counted as 9 months in length.

There has been increased recognition of psychiatric disorders other than depression that are common during and after pregnancy, such as anxiety, bipolar disorders, eating disorders, alcohol use disorders, substance use disorder, stress-related disorders, and psychosis [23–28]. While some of these disorders were characterized in the Australian study which found a higher hospitalization rate after pregnancy [22], most patients in practice require no inpatient care. Further, the timing and incidence rates of these disorders during the perinatal period largely remains unclear.

Here, we aim to describe the calendar time trends and assessed the risk of clinically diagnosed maternal psychiatric disorders, both overall and type-specific, during and after pregnancy, as compared to such risks before pregnancy, using both primary and specialist healthcare registers in Sweden. Herein, we use the terms ‘woman,’ ‘mother,’ and ‘maternal’ as they align with the terminology used in the original dataset, and for ease and legibility, although we recognize that not all birthing individuals identify accordingly.

## Material and methods

### Study design

We conducted a nationwide cohort study including all women who gave birth during 2003–2019 in Sweden (1,854,187 pregnancies in 1,071,086 women) according to the Medical Birth Register (MBR). The MBR is a high-quality register that contains information from prenatal and postpartum clinic visits since 1973 and covers virtually all births in Sweden [29, 30]. After excluding erroneous records (*n* = 29,318), and abundant records for multiple gestation (*n* = 25,859), 1,799,010 pregnancies from 1,052,977 women remained. Using the women’s unique personal identity number [31], the MBR was linked to other registers. All women were then followed from one year before pregnancy, immigration, or January 1, 2003, whichever came later, until one year after pregnancy, emigration, death, or December 31, 2019, whichever came first.

### Ascertainment of preconception, pregnancy, and postpartum periods

We defined three time periods: preconception (the year before pregnancy), antepartum (from estimated start of pregnancy to the delivery), and postpartum (the year after the delivery). The antepartum and postpartum periods were considered risk periods, while the preconception period was considered as the reference period. Due to data holder’s policy, only month and year of delivery were provided. Date of delivery was imputed using a validated method (accuracy = 98% for delivery date within ± 3 days) based on the dates of admission and discharge to the delivery ward and mode of delivery (Supplementary Methods). From this imputed date of delivery, the estimated gestational length recorded in the MBR (mostly based on the routine ultrasound assessment at gestational week 18) was used to calculate retrospectively for the estimated date of start of pregnancy at two weeks before conception. As the study includes deliveries from 2003–2019, preconception data is only available up to 2018.

### Ascertainment of psychiatric disorders

We identified any incident diagnosis of psychiatric disorders recorded in the National Patient Register (NPR; specialist care, with a positive predictive value of 85–95% [32]) and regional primary care registers during the follow-up (ICD-10: F10–F99 during follow-up, and ICD-9 and 8 to confirm incidence) and excluded all pregnancies with a diagnosis of psychiatric disorders recorded more than one year before estimated date of start of pregnancy (*n* = 306,008 in analyses of any psychiatric disorder). The NPR has collected data from inpatient care since 1973 and data from specialist outpatient visits since 2001. The primary care register data were available from the three most populated Swedish counties, namely Stockholm from 2003, Skåne from 2003, and Västra Götaland from 2005. During the study period, 2003–2019, 1,005,886 (54.2%) pregnancies were from these counties. To avoid inclusion of invalid, non-incident diagnoses registered within maternal healthcare as part of anamnesis during obstetric inpatient episodes, and to avoid surveillance bias, we excluded all diagnoses set at obstetrics and gynecology facilities. We further classified psychiatric disorders into seven subgroups: depression, anxiety, stress-related disorders, psychosis, bipolar disorder, alcohol use disorder, and other substance use disorders (ICD-codes are presented in Supplementary Table 1).

### Covariates

We derived age, calendar year, and season at delivery using date of birth and date of delivery. We also obtained data on country of birth, educational level, region of residence, and civil status from the Total Population Register [33] and the longitudinal integrated database for health insurance and labour market (LISA [34]). Moreover, information on smoking three months before pregnancy, body mass index (BMI) in early pregnancy, parity in early pregnancy, diabetic and hypertensive disorders (ICD-codes are presented in Supplementary Table 1), multiple gestation, mode of delivery, gestational length, and birthweight was extracted from the MBR.

### Statistical analysis

We censored the follow-up at the first diagnosis of any psychiatric disorder, if any, in the analysis of overall psychiatric disorder, and the first diagnosis of the corresponding disorder for the type-specific analysis. To illustrate trends over calendar time, we first calculated age-standardized incidence rate (SIR) of overall and type-specific psychiatric disorder before (preconception), during (antepartum), and after (postpartum) pregnancy, yearly from 2003–2019.

Next, we calculated SIR of psychiatric disorders, by week across preconception, antepartum, and postpartum periods. Moreover, we estimated the incidence rate ratio (IRR) of psychiatric disorders in antepartum and postpartum periods compared with the preconception period, by every five weeks. Due to few events, IRR was not calculated for > 40 weeks in antepartum period. We constructed three different models. Model 1, due to the standardization, was adjusted for age and calendar year at delivery and week at follow-up. Model 2 was additionally adjusted for demographics, including country of birth, region of residence, educational level, and season at follow-up. In Model 3 we additionally adjusted for pregnancy characteristics, including civil status in early pregnancy, smoking before pregnancy, BMI category in early pregnancy, multiple gestation, hypertensive disease, diabetes, and parity in early pregnancy. Because most variables in Model 3 happened after the preconception period, Model 2 was considered the primary model.

To evaluate possible effects of the introduction of national guidelines for postpartum depressive symptom screening at 6–8 weeks postpartum introduced in 2010, we performed analyses of any psychiatric disorder and depression, stratified on year of delivery before and after 2010.

### Additional analyses

To also study the type-specific psychiatric disorders as the first-ever psychiatric disorder, we reproduced the type-specific analysis by excluding those with a history of any psychiatric disorder.

Because primary care data were not available nationwide, in another additional analysis, we restricted to three counties where both primary care and specialist care data were available. For this analysis, we applied a one-year wash-out period and additionally censored the woman if she moved out of these counties. This analysis included data from Stockholm; 431,547 (46.9%) pregnancies with births between 2004–2019; Skåne 228,365 (24.8%) pregnancies with births between 2004–2019, and Västra Götaland; 260,686 (28.3%) pregnancies with births between 2006–2019, resulting in 920,598 pregnancies in total.

Finally, to investigate potential effect modification, we stratified the analyses of any psychiatric disorder and depression by educational level, parity at start of pregnancy, and country of birth.

## Results

Characteristics of the included women and pregnancies are presented in Table 1. The majority of women were born in Sweden, lived in the central part of Sweden, had more than 13 years of education, and were non-smoking before pregnancy. The women were more likely to be cohabiting, give birth to their first-born, and have a normal BMI during early pregnancy, than otherwise. They gave birth at an average age of 31.86 years.

**Table 1:** Characteristics of women and pregnancies in Sweden, 2003–2019.

		N of pregnancies	% of pregnancies
**Total**		1 799 010	100.0
Country of birth	Sweden	1 374 164	76.4
	Europe	132 137	7.3
	Other	292 709	16.3
*Before pregnancy:*			
Region of residence	South	409 895	22.8
	Central	1 105 329	61.4
	North	283 786	15.8
Educational level, years	<10	187 580	10.4
	10–12	668 109	37.1
	≥13	875 293	48.7
	Unknown	68 028	3.8
Smoking^a^	No	1 446 826	80.4
	1–9 cigarettes per day	130 278	7.2
	≥10 cigarettes per day	128 589	7.1
	Unknown	93 317	5.2
*During pregnancy:*			
Civil status	Cohabitated	1 681 634	93.5
	Non-cohabitated	117 376	6.5
Parity	0	790 911	44.0
	1	665 258	37.0
	2	239 187	13.3
	≥3	103 654	5.8
BMI, kg/m^2^	<18.5	39 531	2.2
	18.5 to < 25	975 159	54.2
	25 to < 30	428 486	23.8
	≥30	199 035	11.1
	Unknown	156 799	8.7
Multiple gestation	Yes	25 677	1.4
	No	1 773 333	98.6
Hypertensive disease	No	1 734 687	96.4
	Essential hypertension	11 270	0.6
	Preeclampsia	53 053	2.9
Diabetes	No	1 762 896	98.0
	Gestational diabetes	24 834	1.4
	Pregestational diabetes	11 280	0.6
*At delivery:*			
Age, years	Mean and SD	31.86	5.16
	<20	24 139	1.3
	20–24	218 898	12.2
	25–29	543 459	30.2
	30–34	623 754	34.7
	35–39	318 371	17.7
	≥40	70 389	3.9
Calendar year	2003–2005	288 785	16.1
	2006–2010	522 092	29.0
	2011–2015	542 653	30.2
	2016–2019	445 480	24.8
Season at start of follow-up	Spring (Mar to May)	475 882	26.5
	Summer (June to Aug)	478 656	26.6
	Autumn (Sept to Nov)	427 622	23.8
	Winter (Dec to Feb)	416 850	23.2
Birth weight, grams	≥2500	1 728 337	96.1
	1500 to < 2500	54 787	3.0
	<1500	13 414	0.7
	Unknown	2 472	0.1
Gestational length, weeks	<32	15 821	0.9
	32–36	81 313	4.5
	37–41	1 578 980	87.8
	≥42	122 382	6.8
	Unknown	514	0.0
Mode of delivery	Cesarean section	309 274	17.2
	Assisted vaginal delivery	122 717	6.8
	Unassisted vaginal delivery	1 367 019	76.0

*N* number of observations, *BMI* body mass index, *SD* standard deviation.

^a^Smoking three months before pregnancy.

### Trends over calendar time

**Fig. 1:**
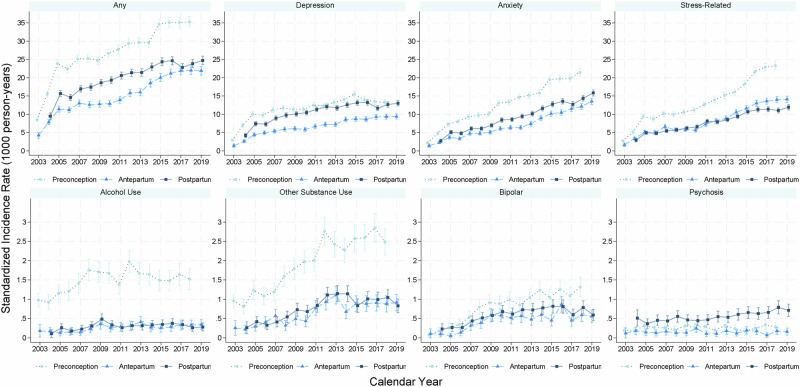
Standardized incidence rate of psychiatric disorders before (preconception), during (antepartum), and after (postpartum) pregnancy during 2003–2019. The incidence rate was standardized by age at delivery.

During the study period, we identified 31,108 (2.30%) first-ever psychiatric disorders diagnosed in preconceptional period, 16,922 (1.16%) in pregnancy period, and 27,612 (1.92%) in postpartum period. From 2003–2019, the preconceptional, antepartum and postpartum SIRs of any psychiatric disorder increased over time, especially in the preconceptional period (Fig. 1 and Supplementary Table 2). Similar patterns were observed for most psychiatric disorders, except for psychosis (all three periods) and alcohol use disorders (antepartum and postpartum periods) where the SIRs were somewhat stable over calendar time. From approximately 2007, the increase in SIRs seems slower for depression than for example anxiety and stress-related disorders.

### Comparisons across the perinatal periods

**Fig. 2:**
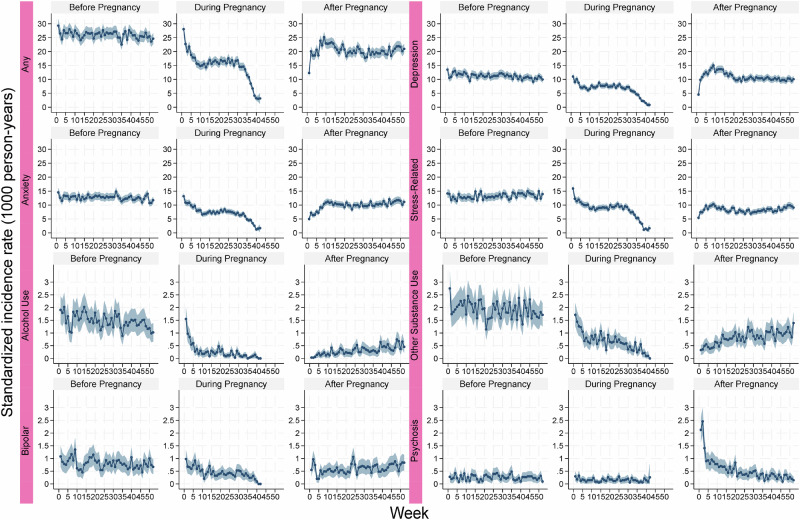
Standardized incidence rate of psychiatric disorders before, during, and after pregnancy by week. The incidence rate was standardized by age and calendar year at delivery.

Across the three perinatal periods, the SIR of any psychiatric disorder was stable over the preconception period at around 25 per 1000 person-years, decreasing across the antepartum period reaching down to 4 per 1000 person-years, before bouncing back to around 20 per 1000 person-years during the postpartum period (Fig. 2 and Supplementary Table 3). A similar trend was found for most types of psychiatric disorders, except for depression and psychosis where an increase was present during the first weeks postpartum.

**Fig. 3:**
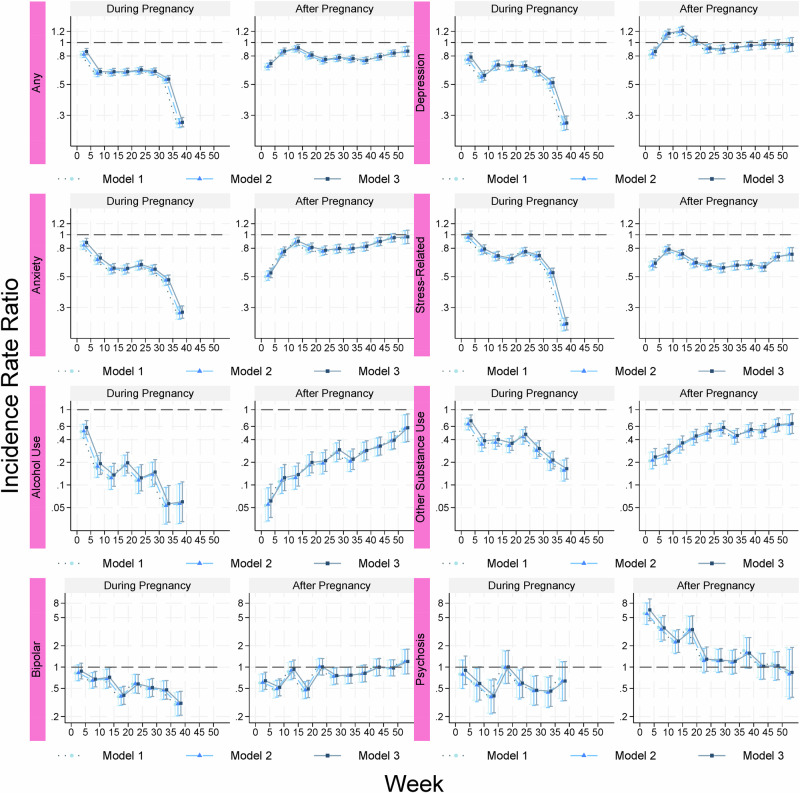
Incidence rate ratio of psychiatric disorders during and after pregnancy compared with before pregnancy. The incidence rate ratio was estimated by every 5 weeks. Model 1 was adjusted for age and calendar year at delivery and week at follow-up. Model 2 was additionally adjusted for country of birth, region of residence, educational level, and season at follow-up. Model 3 was additionally adjusted for civil status, smoking, BMI category, multiple gestation, hypertensive disease, diabetes, and parity.

Compared to the corresponding weeks in preconception period, the risk of any psychiatric disorder gradually decreased during antepartum period in all three models (Fig. 3). During the postpartum period, the risk remained reduced although the IRR attenuated towards null by the end of the postpartum year. Notably, the risk of depression increased by 20% from 5–15 weeks postpartum, while the risk of psychosis was 6–7 times higher within first 5 weeks postpartum and remained doubled during week 5–20 postpartum, compared to before pregnancy (Supplementary Table 4). Other subtypes showed a pattern comparable to that of any psychiatric disorder. The attenuation towards null during the postpartum period was particularly pronounced for alcohol and other substance use disorders.

**Fig. 4:**
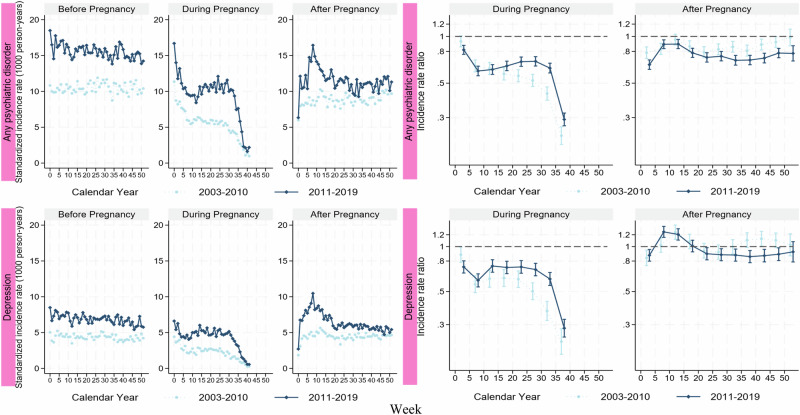
Standardized incidence rate and incidence rate ratio of any psychiatric disorder, and depression, stratified on year of delivery. The incidence rate was standardized by age and calendar year at delivery. The incidence rate ratio was estimated by every 5 weeks and adjusted for age and calendar year at delivery, week at follow-up, country of birth, region of residence, educational level, season at follow-up, civil status, smoking, BMI category, multiple gestation, hypertensive disease, diabetes, and parity.

When stratifying the analyses by year of delivery, the SIRs of both any psychiatric disorder and depression were in general higher throughout the three periods during 2011–2019 than 2003–2010 (Fig. 4). During the postpartum period, the SIRs peaked at around week 8 during 2011–2019 but peaked at around week 13 during 2003–2010. Despite such shift over time, the change in IRRs, in comparison to corresponding preconceptional weeks, was less pronounced (Supplementary Table 5).

Results remained largely unchanged after excluding women with a history of any other psychiatric disorders in the type-specific analyses (Supplementary Fig. 1), and when restricting the analysis to three counties with both primary care and specialist care data (Supplementary Fig. 2 and Supplementary Fig. 3).

In stratified analyses, higher SIRs of both any psychiatric disorder and depression throughout the three periods were noted among women with a lower educational attainment, while the IRRs during and after pregnancy were lowest for this group (Supplementary Fig. 4). The risks of both any psychiatric disorder and depression were not clearly modified by parity (Supplementary Fig. 5) or by country of birth (Supplementary Fig. 6).

## Discussion

In this nationwide cohort including 1,799,010 pregnancies from 1,052,977 women, we found that the incident rates of maternal psychiatric disorder before, during, and after pregnancy increased from 2003–2019. However, the risk of being diagnosed with any psychiatric disorder was lower during and after pregnancy compared to before pregnancy. This pattern was observed for all types of psychiatric disorders, except for depression and psychosis, of which an increased risk was noted during 5–15 and 0–20 postpartum weeks, respectively. These results provide valuable insights to identify high-risk time windows for early detection and potential intervention of maternal perinatal psychiatric disorders.

The increase in psychiatric disorders over time is alarming, but in line with reports of non-perinatal psychiatric disorders globally [35], and with preconceptional mental disorders [17]. Such trend could partly be explained by the population change in lifestyle (including working culture, less physical activity, chronic health issues such as diabetes mellitus, obesity, and chronic hypertension, and high social media usage, all linked to mental health [36–38]), as well as increased awareness, optimization of diagnosing, and reduced stigma over time. Screening for depressive symptoms with the Edinburgh Postnatal Depression Scale (EPDS) [39] at 6–8 weeks postpartum was gradually introduced in Sweden since the nineties, and has been implemented nationwide since 2010 [40]. Lately, screening for antepartum depressive symptoms has been introduced in some counties in Sweden, potentially leading to more women with depression being detected and diagnosed. However, the screening could also prevent women with subclinical depression from developing a depressive episode by providing evidence-based care. Those clinical routines may explain the slower increase in the incidence of perinatal depression, compared to other psychiatric disorders, noted over the past decade.

When compared to preconceptional period, we noted a lower risk of overall psychiatric disorders during both antepartum and postpartum periods. This lower risk during antepartum period is in line with the recorded hospital admission rate in Australia; however, the same study found a higher hospital admission rate postpartum than the preconceptional period [22]. Other Swedish population-based studies have suggested that pregnancy, and transition to motherhood, has a protective effect on psychiatric disorders [41, 42]. This protective effect is possibly due to lifestyle and social changes, but also alterations in biological systems, such as oxytocin [43] and estrogen [44]. In addition, the continuous prenatal checkups and the relatively accessible support provided for mental health during pregnancy might also contribute [45]. In the present study, we used the preconceptional year as the reference, arguably deemed as a healthier period, particularly for planned pregnancy, compared to general population [46]. As many couples struggle to conceive (approximately 15% needs assisted reproductive technology to succeed), this period could however be stressful for some and possibly associated with a higher risk of psychiatric disorders than at other times in life [47, 48]. However, in the present study, the IR of psychiatric disorders appeared to be quite stable across the preconceptional weeks.

Previous studies have shown highest incidence rate of postpartum depression during the first months postpartum [49]. Further, prevalence of positive screening for depressive symptoms during the 12th month postpartum has been suggested as high as in the first month postpartum [50], and trajectories of depressive symptoms has been suggested as stable across the first two years postpartum [51]. Whereas such prevalence estimates can be informative, these data do not indicate when the new cases emerged, which matters for clinical resource allocation. Our data showed a higher risk after delivery compared to the preconceptional period. This finding is largely comparable with both the Danish [21] and Australian [22] studies, although these two studies did not assess the relative risk. We found that the risk of depression increased by 20% during postpartum weeks 5–15, compared to the corresponding weeks in the preconceptional period. While this trend may be supported by the well-known hormone withdrawal theory [8] and inflammatory responses [9], other factors such as sleep deprivation and adaptation to the new family constellation could contribute [10]. Moreover, this peak can be attributed to the screening deployed at 6–8 weeks postpartum in Sweden. Importantly, we observed a 4-week shift of the peak risk towards childbirth after the nationwide screening was introduced. Although depression diagnosis is believably often later than the disease onset, this finding supports the potential benefit of screening for early detection. If proper care is provided in time to those in need, this could save a tremendous suffering time and avoid negative impacts on the patients [52].

For psychosis, the drastic peak observed postpartum clearly highlights the vulnerability during early postpartum period. This peak has been observed [53] also in women without previous severe psychiatric disorders [54]. Postpartum psychosis can have long-term impact on the woman and baby [55, 56] and, although progress has been made [57], effective interventions or preventions have been long missing [58, 59].

In addition, a remarkable increase in incidence rate of substance use disorders was observed in the preconceptional period over the study period. This increase may be due to increased availability to substance, especially for cannabis, in Sweden during the past two decades [60], in the context of reduced stigma in public. Reassuringly, our results and other studies [41] have suggested a lower risk during pregnancy and after childbirth, although the underreporting during this sensitive period cannot be ruled out.

Previous studies have shown a high prevalence of antepartum depression when including self-reported depressive symptoms [61]. While our results found a lower risk of depression during pregnancy compared to preconceptional period, screening for depressive symptoms during pregnancy remains valid for the detection of prevalent untreated major depression, which could have negative impact on pregnancy outcomes [62, 63]. Moreover, although the risk for most psychiatric disorders was lower during pregnancy and postpartum, the impact of the diseases at this specific time in life may have more severe consequences than at other times. This is not only true for the mother [64], but also for the baby [1], the family [65] and the society [66]. Given the fact that these women are under surveillance through maternity care, potential preventions and interventions could be integrated to the existing routines for further improvements.

### Strengths and limitations

Our study has many strengths. First, the large sample size, including all birthing women in Sweden, allows for powerful investigation on less common psychiatric disorders. Moreover, the exposure window is precisely defined due to the estimation of gestational length and conception, limiting the risk of misclassification of the diagnosis of psychiatric disorders before and during pregnancy. Notably, the timing of diagnosis is often later than the disease onset, although we aimed to provide evidence for timely detection and prevention in clinical practice. Although pregnant and postpartum women are in contact with healthcare professionals on a regular basis, our results are unlikely explained by surveillance bias as we have noted a lower risk of psychiatric disorders overall during antepartum and postpartum periods, compared to before pregnancy. Our study has some limitations. Although we sourced primary care data of the three most populated counties in Sweden, covering > 50% of the study population, we might have missed psychiatric disorders diagnosed both before and during the perinatal period by general practitioners in other counties, and psychiatric disorders handled within maternity care. However, an additional analysis restricted to these three counties yielded similar patterns, and psychiatric disorders are rarely diagnosed within maternity care. Our study population consist of women who became pregnant and maintained their pregnancies. Notably, women newly diagnosed with a psychiatric disorder might choose not to become pregnant or to terminate their pregnancy, and hence, are not included in this study. Lastly, Sweden is a high-income country with high quality and tax-funded universal healthcare. Our findings may not be generalized to countries with different healthcare settings and screening strategies.

## Conclusions

Our data highlighted an alarmingly increased rate of maternal psychiatric disorders before, during, and after pregnancy over time. Although the risk of maternal psychiatric disorders was lower during and after pregnancy, compared to before pregnancy, the risk of depression and psychosis increased shortly after the delivery. Throughout all stages of life, perinatal periods are the vital points that women are in frequent contacts with the healthcare system. Potential practical approaches can be adopted for education, risk assessment, and early detection and intervention, with the goal of improving maternal mental health for the well-being of the mothers and families.

## Data avaliability

Swedish register data can only be accessed after granted ethical approval by appropriate authorities due to privacy protection governed by the General Data Protection Regulation. Information can be found at the Swedish National Board of Health and Welfare (https://bestalladata.socialstyrelsen.se/, email: registerservice@socialstyrelsen.se) and/or Statistics Sweden (https://www.scb.se/vara-tjanster/bestall-data-och-statistik/, email: scb@scb.se). To access data from primary care registers in Sweden, separate applications to each region is needed (information can be found here: https://kliniskastudier.se/).

## Supplementary information

Supplementary material

## Data Availability

Data management was conducted using SAS (version 9.4) while statistical analyses were performed using STATA (version 17). Code is available upon reasonable request to corresponding author.
