# Supplementary material for: Maternal psychiatric disorders before, during, and after pregnancy: a national cohort study in Sweden
Source: Mol Psychiatry. 2025 Sep 25;31(1):309–17. doi: 10.1038/s41380-025-03212-9 (PMC12700809; doi:10.1038/s41380-025-03212-9)
Supplement: Supplementary file 1 — Supplementary material [file 41380_2025_3212_MOESM1_ESM.docx]

**Supplementary Information**

**Maternal psychiatric disorders before, during, and after pregnancy: a national cohort study in Sweden**

Running title: Maternal perinatal psychiatric disorders

Emma Bränn, PhD^1,2^, Jerry Guintivano, PhD^3^, Yihui Yang, MS^1^, Louise Lundborg, PhD^4^, Marion Opatowski, PhD^1^, Fang Fang, PhD^1^, Unnur A. Valdimarsdóttir, PhD^1,5,6^, Emma Fransson, PhD^7,8^, Alkistis Skalkidou, MD, PhD^7^, Yi Lu, PhD^9^, Donghao Lu, MD, PhD^1^

1. Institute of Environmental Medicine, Karolinska Institutet, Stockholm, Sweden
2. Center for Epidemiology and Community Medicine, Region Stockholm, Stockholm, Sweden.
3. Department of Psychiatry, University of North Carolina at Chapel Hill, Chapel Hill, NC, USA.
4. Clinical Epidemiology Division, Department of Medicine, Karolinska Institutet, Stockholm, Sweden
5. Center of Public Health Sciences, Faculty of Medicine, University of Iceland, Reykjavík, Iceland
6. Department of Epidemiology, Harvard TH Chan School of Public Health, Harvard University, Boston, Massachusetts, USA
7. Department of Women´s and Children´s health, Uppsala University, Uppsala, Sweden
8. Centre for Translational Microbiome Research, Department of Microbiology, Tumor and Cell Biology, Karolinska Institutet, Stockholm, Sweden
9. Department of Medical Epidemiology and Biostatistics, Karolinska Institutet, Stockholm, Sweden

This supplementary information presents information about imputation of delivery date, ICD-codes used, tables with estimates presented in the graphs in the main figures, and figures for additional analyses performed.

**Supplementary Information**

**Supplementary Methods: Imputation of birth date**

Due to ethical considerations, only birth month and year was available, hence, date of birth was imputed based on the admission and discharge dates to the delivery ward. If the length of stay was within 2 days, the admission date was used as the date of delivery. If the length of stay was between 3 and 6 days, the date of delivery was imputed based on parity and mode of delivery by using the date of discharge minus the mean length of stay from reports (<https://www.socialstyrelsen.se/globalassets/sharepoint-dokument/artikelkatalog/ovrigt/2017-4-13.pdf>). For outliers of length of stay, 1 day after the admission was estimated as the date of delivery. In a subset of the data (767,760 pregnancies), we had access to the exact delivery date and were able to run a validation test. We found 98% of the pregnancies had imputed delivery date within ± 3 days, 96% of the pregnancies had imputed delivery date within ± 2 days, 76% of the pregnancies had imputed delivery date within ± 1 days, and 62% of the pregnancies had imputed delivery date exactly at the true delivery date.

| **Supplementary Table 1.** Swedish International Classification of Diseases codes used to identify psychiatric and pregnancy related diseases. | | | |
| --- | --- | --- | --- |
|  | **ICD-8** | **ICD-9** | **ICD-10** |
| **Psychiatric disorders** |  |  |  |
| Any | **-** | 290-319 | F10-F99 |
| Depression | 3004, 300E | 300E, 311 | F32-F33, F53.0 |
| Anxiety | 3000, 3002, | 300A, 300C | F40-F41 |
| Stress-related disorder | 307 | 308-309 | F43 |
| Psychosis | 291-292, 295, 297-299 | 2960, 2962, 2969, 296B, 296X | F20-F29, F531 |
| Bipolar disorder | 296 | 296A, 296C, 296D, 296E, 296W | F30-F31 |
| Alcohol use disorder | 303 | 305A | F10 |
| Other substance use disorder | 304 | 305X | F11-F19 |
| **Other diseases** |  |  |  |
| Preeclampsia | 63703-99 | 642E-G | O14-15 |
| Essential hypertension | 400-404 | 401-405, 642A-642C, 642H | O10- O11, I10-I-15 |
| Pregestational diabetes | 250 | 648A, O240-O234 | E10-E14 |
| Gestational diabetes | - | 648W | O244 |
| ICD = International Classification of Diseases | | | |

**Supplementary Table 2.** Standardized incidence rate and 95% confidence intervals of psychiatric disorders before (preconception), during (antepartum), and after (postpartum) pregnancy during 2003-2019.

| **Psychiatric disorder** | **Phase** | **Year** | **SIR** | **95% CI** |
| --- | --- | --- | --- | --- |
| Any | Preconception | 2003 | 8.51 | (7.97 - 9.05) |
| Any | Preconception | 2004 | 15.51 | (14.65 - 16.37) |
| Any | Preconception | 2005 | 23.91 | (22.8 - 25.02) |
| Any | Preconception | 2006 | 22.42 | (21.35 - 23.5) |
| Any | Preconception | 2007 | 25.15 | (24.01 - 26.28) |
| Any | Preconception | 2008 | 25.28 | (24.14 - 26.42) |
| Any | Preconception | 2009 | 24.75 | (23.62 - 25.88) |
| Any | Preconception | 2010 | 26.67 | (25.5 - 27.84) |
| Any | Preconception | 2011 | 27.78 | (26.56 - 29) |
| Any | Preconception | 2012 | 29.43 | (28.16 - 30.69) |
| Any | Preconception | 2013 | 29.70 | (28.41 - 30.98) |
| Any | Preconception | 2014 | 29.57 | (28.28 - 30.87) |
| Any | Preconception | 2015 | 34.65 | (33.24 - 36.06) |
| Any | Preconception | 2016 | 35.12 | (33.68 - 36.56) |
| Any | Preconception | 2017 | 35.12 | (33.65 - 36.58) |
| Any | Preconception | 2018 | 35.31 | (33.84 - 36.78) |
| Any | Antepartum | 2003 | 4.25 | (3.46 - 5.03) |
| Any | Antepartum | 2004 | 7.86 | (7.19 - 8.53) |
| Any | Antepartum | 2005 | 11.41 | (10.61 - 12.21) |
| Any | Antepartum | 2006 | 11.22 | (10.43 - 12.02) |
| Any | Antepartum | 2007 | 13.03 | (12.17 - 13.89) |
| Any | Antepartum | 2008 | 12.62 | (11.78 - 13.47) |
| Any | Antepartum | 2009 | 12.80 | (11.95 - 13.65) |
| Any | Antepartum | 2010 | 12.94 | (12.09 - 13.79) |
| Any | Antepartum | 2011 | 13.93 | (13.04 - 14.82) |
| Any | Antepartum | 2012 | 15.83 | (14.87 - 16.8) |
| Any | Antepartum | 2013 | 16.03 | (15.06 - 17.01) |
| Any | Antepartum | 2014 | 18.55 | (17.49 - 19.61) |
| Any | Antepartum | 2015 | 20.08 | (18.97 - 21.19) |
| Any | Antepartum | 2016 | 21.23 | (20.08 - 22.37) |
| Any | Antepartum | 2017 | 21.94 | (20.77 - 23.12) |
| Any | Antepartum | 2018 | 22.03 | (20.84 - 23.22) |
| Any | Antepartum | 2019 | 21.93 | (20.73 - 23.14) |
| Any | Postpartum | 2004 | 9.49 | (8.59 - 10.38) |
| Any | Postpartum | 2005 | 15.71 | (14.89 - 16.54) |
| Any | Postpartum | 2006 | 14.62 | (13.82 - 15.42) |
| Any | Postpartum | 2007 | 16.95 | (16.09 - 17.82) |
| Any | Postpartum | 2008 | 17.46 | (16.59 - 18.34) |
| Any | Postpartum | 2009 | 18.66 | (17.76 - 19.57) |
| Any | Postpartum | 2010 | 19.36 | (18.43 - 20.28) |
| Any | Postpartum | 2011 | 20.60 | (19.65 - 21.55) |
| Any | Postpartum | 2012 | 21.38 | (20.41 - 22.36) |
| Any | Postpartum | 2013 | 21.45 | (20.46 - 22.45) |
| Any | Postpartum | 2014 | 22.97 | (21.93 - 24) |
| Any | Postpartum | 2015 | 24.39 | (23.31 - 25.47) |
| Any | Postpartum | 2016 | 24.68 | (23.59 - 25.78) |
| Any | Postpartum | 2017 | 22.84 | (21.78 - 23.89) |
| Any | Postpartum | 2018 | 23.86 | (22.77 - 24.95) |
| Any | Postpartum | 2019 | 24.77 | (23.65 - 25.9) |
| Depression | Preconception | 2003 | 2.93 | (2.62 - 3.24) |
| Depression | Preconception | 2004 | 6.90 | (6.33 - 7.46) |
| Depression | Preconception | 2005 | 10.01 | (9.3 - 10.71) |
| Depression | Preconception | 2006 | 9.78 | (9.09 - 10.47) |
| Depression | Preconception | 2007 | 11.22 | (10.49 - 11.96) |
| Depression | Preconception | 2008 | 11.72 | (10.98 - 12.47) |
| Depression | Preconception | 2009 | 11.40 | (10.67 - 12.13) |
| Depression | Preconception | 2010 | 11.38 | (10.65 - 12.1) |
| Depression | Preconception | 2011 | 12.49 | (11.72 - 13.27) |
| Depression | Preconception | 2012 | 12.38 | (11.61 - 13.15) |
| Depression | Preconception | 2013 | 13.27 | (12.47 - 14.07) |
| Depression | Preconception | 2014 | 14.06 | (13.23 - 14.89) |
| Depression | Preconception | 2015 | 15.52 | (14.65 - 16.39) |
| Depression | Preconception | 2016 | 13.97 | (13.14 - 14.8) |
| Depression | Preconception | 2017 | 13.49 | (12.66 - 14.31) |
| Depression | Preconception | 2018 | 13.27 | (12.45 - 14.09) |
| Depression | Antepartum | 2003 | 1.42 | (0.97 - 1.87) |
| Depression | Antepartum | 2004 | 2.63 | (2.25 - 3.01) |
| Depression | Antepartum | 2005 | 4.40 | (3.91 - 4.89) |
| Depression | Antepartum | 2006 | 4.94 | (4.43 - 5.45) |
| Depression | Antepartum | 2007 | 5.40 | (4.86 - 5.93) |
| Depression | Antepartum | 2008 | 5.94 | (5.39 - 6.5) |
| Depression | Antepartum | 2009 | 6.10 | (5.54 - 6.66) |
| Depression | Antepartum | 2010 | 5.86 | (5.32 - 6.41) |
| Depression | Antepartum | 2011 | 6.70 | (6.12 - 7.28) |
| Depression | Antepartum | 2012 | 7.25 | (6.64 - 7.86) |
| Depression | Antepartum | 2013 | 7.27 | (6.65 - 7.88) |
| Depression | Antepartum | 2014 | 8.57 | (7.9 - 9.24) |
| Depression | Antepartum | 2015 | 8.75 | (8.08 - 9.43) |
| Depression | Antepartum | 2016 | 8.64 | (7.98 - 9.31) |
| Depression | Antepartum | 2017 | 9.28 | (8.58 - 9.97) |
| Depression | Antepartum | 2018 | 9.35 | (8.65 - 10.06) |
| Depression | Antepartum | 2019 | 9.39 | (8.68 - 10.1) |
| Depression | Postpartum | 2004 | 4.22 | (3.63 - 4.8) |
| Depression | Postpartum | 2005 | 7.44 | (6.88 - 8) |
| Depression | Postpartum | 2006 | 7.32 | (6.77 - 7.87) |
| Depression | Postpartum | 2007 | 8.95 | (8.35 - 9.56) |
| Depression | Postpartum | 2008 | 9.77 | (9.14 - 10.4) |
| Depression | Postpartum | 2009 | 10.10 | (9.47 - 10.74) |
| Depression | Postpartum | 2010 | 10.47 | (9.83 - 11.12) |
| Depression | Postpartum | 2011 | 11.36 | (10.7 - 12.03) |
| Depression | Postpartum | 2012 | 12.12 | (11.43 - 12.81) |
| Depression | Postpartum | 2013 | 11.77 | (11.08 - 12.46) |
| Depression | Postpartum | 2014 | 12.62 | (11.91 - 13.33) |
| Depression | Postpartum | 2015 | 13.20 | (12.47 - 13.93) |
| Depression | Postpartum | 2016 | 13.24 | (12.51 - 13.98) |
| Depression | Postpartum | 2017 | 11.67 | (10.98 - 12.35) |
| Depression | Postpartum | 2018 | 12.69 | (11.97 - 13.4) |
| Depression | Postpartum | 2019 | 13.00 | (12.27 - 13.73) |
| Anxiety | Preconception | 2003 | 2.23 | (1.96 - 2.5) |
| Anxiety | Preconception | 2004 | 4.78 | (4.31 - 5.24) |
| Anxiety | Preconception | 2005 | 7.30 | (6.7 - 7.9) |
| Anxiety | Preconception | 2006 | 8.08 | (7.46 - 8.7) |
| Anxiety | Preconception | 2007 | 9.25 | (8.59 - 9.92) |
| Anxiety | Preconception | 2008 | 9.76 | (9.08 - 10.43) |
| Anxiety | Preconception | 2009 | 10.11 | (9.43 - 10.8) |
| Anxiety | Preconception | 2010 | 13.08 | (12.3 - 13.85) |
| Anxiety | Preconception | 2011 | 13.39 | (12.6 - 14.19) |
| Anxiety | Preconception | 2012 | 14.69 | (13.86 - 15.53) |
| Anxiety | Preconception | 2013 | 15.20 | (14.35 - 16.05) |
| Anxiety | Preconception | 2014 | 15.85 | (14.97 - 16.72) |
| Anxiety | Preconception | 2015 | 19.51 | (18.54 - 20.49) |
| Anxiety | Preconception | 2016 | 19.76 | (18.77 - 20.75) |
| Anxiety | Preconception | 2017 | 19.88 | (18.87 - 20.89) |
| Anxiety | Preconception | 2018 | 21.49 | (20.44 - 22.55) |
| Anxiety | Antepartum | 2003 | 1.43 | (0.99 - 1.88) |
| Anxiety | Antepartum | 2004 | 2.27 | (1.92 - 2.62) |
| Anxiety | Antepartum | 2005 | 3.69 | (3.24 - 4.14) |
| Anxiety | Antepartum | 2006 | 3.35 | (2.93 - 3.77) |
| Anxiety | Antepartum | 2007 | 4.76 | (4.26 - 5.25) |
| Anxiety | Antepartum | 2008 | 4.78 | (4.29 - 5.28) |
| Anxiety | Antepartum | 2009 | 5.08 | (4.58 - 5.59) |
| Anxiety | Antepartum | 2010 | 6.11 | (5.56 - 6.66) |
| Anxiety | Antepartum | 2011 | 6.35 | (5.79 - 6.91) |
| Anxiety | Antepartum | 2012 | 6.39 | (5.82 - 6.96) |
| Anxiety | Antepartum | 2013 | 7.41 | (6.79 - 8.02) |
| Anxiety | Antepartum | 2014 | 8.97 | (8.29 - 9.65) |
| Anxiety | Antepartum | 2015 | 10.17 | (9.44 - 10.89) |
| Anxiety | Antepartum | 2016 | 10.48 | (9.74 - 11.22) |
| Anxiety | Antepartum | 2017 | 11.52 | (10.74 - 12.29) |
| Anxiety | Antepartum | 2018 | 12.16 | (11.35 - 12.97) |
| Anxiety | Antepartum | 2019 | 13.56 | (12.7 - 14.42) |
| Anxiety | Postpartum | 2004 | 2.75 | (2.28 - 3.22) |
| Anxiety | Postpartum | 2005 | 5.11 | (4.65 - 5.57) |
| Anxiety | Postpartum | 2006 | 4.81 | (4.37 - 5.26) |
| Anxiety | Postpartum | 2007 | 6.08 | (5.58 - 6.58) |
| Anxiety | Postpartum | 2008 | 6.12 | (5.62 - 6.62) |
| Anxiety | Postpartum | 2009 | 6.99 | (6.47 - 7.52) |
| Anxiety | Postpartum | 2010 | 8.47 | (7.89 - 9.05) |
| Anxiety | Postpartum | 2011 | 8.60 | (8.02 - 9.17) |
| Anxiety | Postpartum | 2012 | 9.46 | (8.85 - 10.06) |
| Anxiety | Postpartum | 2013 | 10.19 | (9.55 - 10.82) |
| Anxiety | Postpartum | 2014 | 11.62 | (10.94 - 12.3) |
| Anxiety | Postpartum | 2015 | 12.77 | (12.05 - 13.48) |
| Anxiety | Postpartum | 2016 | 13.58 | (12.84 - 14.32) |
| Anxiety | Postpartum | 2017 | 12.73 | (12.01 - 13.44) |
| Anxiety | Postpartum | 2018 | 14.39 | (13.62 - 15.16) |
| Anxiety | Postpartum | 2019 | 15.86 | (15.04 - 16.68) |
| Alcohol Use | Preconception | 2003 | 0.98 | (0.8 - 1.16) |
| Alcohol Use | Preconception | 2004 | 0.93 | (0.72 - 1.13) |
| Alcohol Use | Preconception | 2005 | 1.15 | (0.92 - 1.39) |
| Alcohol Use | Preconception | 2006 | 1.21 | (0.97 - 1.44) |
| Alcohol Use | Preconception | 2007 | 1.41 | (1.16 - 1.67) |
| Alcohol Use | Preconception | 2008 | 1.75 | (1.47 - 2.03) |
| Alcohol Use | Preconception | 2009 | 1.71 | (1.43 - 1.98) |
| Alcohol Use | Preconception | 2010 | 1.68 | (1.41 - 1.95) |
| Alcohol Use | Preconception | 2011 | 1.38 | (1.13 - 1.63) |
| Alcohol Use | Preconception | 2012 | 1.97 | (1.68 - 2.27) |
| Alcohol Use | Preconception | 2013 | 1.67 | (1.4 - 1.95) |
| Alcohol Use | Preconception | 2014 | 1.64 | (1.37 - 1.92) |
| Alcohol Use | Preconception | 2015 | 1.49 | (1.23 - 1.75) |
| Alcohol Use | Preconception | 2016 | 1.48 | (1.21 - 1.75) |
| Alcohol Use | Preconception | 2017 | 1.64 | (1.36 - 1.92) |
| Alcohol Use | Preconception | 2018 | 1.52 | (1.25 - 1.79) |
| Alcohol Use | Antepartum | 2003 | 0.18 | (0.02 - 0.35) |
| Alcohol Use | Antepartum | 2004 | 0.17 | (0.07 - 0.27) |
| Alcohol Use | Antepartum | 2005 | 0.14 | (0.05 - 0.22) |
| Alcohol Use | Antepartum | 2006 | 0.16 | (0.07 - 0.25) |
| Alcohol Use | Antepartum | 2007 | 0.14 | (0.06 - 0.23) |
| Alcohol Use | Antepartum | 2008 | 0.24 | (0.13 - 0.35) |
| Alcohol Use | Antepartum | 2009 | 0.36 | (0.23 - 0.49) |
| Alcohol Use | Antepartum | 2010 | 0.27 | (0.16 - 0.38) |
| Alcohol Use | Antepartum | 2011 | 0.29 | (0.17 - 0.4) |
| Alcohol Use | Antepartum | 2012 | 0.31 | (0.19 - 0.44) |
| Alcohol Use | Antepartum | 2013 | 0.41 | (0.27 - 0.55) |
| Alcohol Use | Antepartum | 2014 | 0.27 | (0.16 - 0.39) |
| Alcohol Use | Antepartum | 2015 | 0.26 | (0.15 - 0.37) |
| Alcohol Use | Antepartum | 2016 | 0.29 | (0.17 - 0.41) |
| Alcohol Use | Antepartum | 2017 | 0.33 | (0.2 - 0.46) |
| Alcohol Use | Antepartum | 2018 | 0.33 | (0.2 - 0.46) |
| Alcohol Use | Antepartum | 2019 | 0.34 | (0.21 - 0.48) |
| Alcohol Use | Postpartum | 2004 | 0.11 | (0.01 - 0.21) |
| Alcohol Use | Postpartum | 2005 | 0.26 | (0.16 - 0.37) |
| Alcohol Use | Postpartum | 2006 | 0.18 | (0.09 - 0.26) |
| Alcohol Use | Postpartum | 2007 | 0.23 | (0.13 - 0.33) |
| Alcohol Use | Postpartum | 2008 | 0.31 | (0.2 - 0.42) |
| Alcohol Use | Postpartum | 2009 | 0.49 | (0.35 - 0.62) |
| Alcohol Use | Postpartum | 2010 | 0.33 | (0.22 - 0.44) |
| Alcohol Use | Postpartum | 2011 | 0.27 | (0.17 - 0.36) |
| Alcohol Use | Postpartum | 2012 | 0.32 | (0.21 - 0.43) |
| Alcohol Use | Postpartum | 2013 | 0.32 | (0.21 - 0.42) |
| Alcohol Use | Postpartum | 2014 | 0.34 | (0.22 - 0.45) |
| Alcohol Use | Postpartum | 2015 | 0.35 | (0.24 - 0.47) |
| Alcohol Use | Postpartum | 2016 | 0.38 | (0.26 - 0.5) |
| Alcohol Use | Postpartum | 2017 | 0.35 | (0.23 - 0.46) |
| Alcohol Use | Postpartum | 2018 | 0.27 | (0.17 - 0.36) |
| Alcohol Use | Postpartum | 2019 | 0.28 | (0.18 - 0.39) |
| Other Substance Use | Preconception | 2003 | 0.96 | (0.78 - 1.13) |
| Other Substance Use | Preconception | 2004 | 0.81 | (0.62 - 1) |
| Other Substance Use | Preconception | 2005 | 1.23 | (0.99 - 1.47) |
| Other Substance Use | Preconception | 2006 | 1.09 | (0.86 - 1.31) |
| Other Substance Use | Preconception | 2007 | 1.19 | (0.96 - 1.42) |
| Other Substance Use | Preconception | 2008 | 1.60 | (1.33 - 1.86) |
| Other Substance Use | Preconception | 2009 | 1.79 | (1.51 - 2.07) |
| Other Substance Use | Preconception | 2010 | 1.98 | (1.68 - 2.27) |
| Other Substance Use | Preconception | 2011 | 2.01 | (1.71 - 2.31) |
| Other Substance Use | Preconception | 2012 | 2.76 | (2.41 - 3.11) |
| Other Substance Use | Preconception | 2013 | 2.42 | (2.09 - 2.76) |
| Other Substance Use | Preconception | 2014 | 2.27 | (1.95 - 2.59) |
| Other Substance Use | Preconception | 2015 | 2.57 | (2.22 - 2.92) |
| Other Substance Use | Preconception | 2016 | 2.60 | (2.25 - 2.94) |
| Other Substance Use | Preconception | 2017 | 2.85 | (2.48 - 3.22) |
| Other Substance Use | Preconception | 2018 | 2.49 | (2.14 - 2.83) |
| Other Substance Use | Antepartum | 2003 | 0.26 | (0.06 - 0.45) |
| Other Substance Use | Antepartum | 2004 | 0.22 | (0.11 - 0.33) |
| Other Substance Use | Antepartum | 2005 | 0.30 | (0.17 - 0.42) |
| Other Substance Use | Antepartum | 2006 | 0.40 | (0.26 - 0.55) |
| Other Substance Use | Antepartum | 2007 | 0.57 | (0.4 - 0.74) |
| Other Substance Use | Antepartum | 2008 | 0.32 | (0.2 - 0.45) |
| Other Substance Use | Antepartum | 2009 | 0.49 | (0.34 - 0.65) |
| Other Substance Use | Antepartum | 2010 | 0.44 | (0.29 - 0.58) |
| Other Substance Use | Antepartum | 2011 | 0.77 | (0.58 - 0.96) |
| Other Substance Use | Antepartum | 2012 | 0.93 | (0.72 - 1.15) |
| Other Substance Use | Antepartum | 2013 | 1.11 | (0.88 - 1.34) |
| Other Substance Use | Antepartum | 2014 | 0.68 | (0.5 - 0.86) |
| Other Substance Use | Antepartum | 2015 | 0.89 | (0.68 - 1.09) |
| Other Substance Use | Antepartum | 2016 | 0.89 | (0.68 - 1.09) |
| Other Substance Use | Antepartum | 2017 | 0.91 | (0.7 - 1.12) |
| Other Substance Use | Antepartum | 2018 | 0.88 | (0.67 - 1.09) |
| Other Substance Use | Antepartum | 2019 | 0.91 | (0.7 - 1.12) |
| Other Substance Use | Postpartum | 2004 | 0.26 | (0.11 - 0.42) |
| Other Substance Use | Postpartum | 2005 | 0.42 | (0.29 - 0.55) |
| Other Substance Use | Postpartum | 2006 | 0.33 | (0.21 - 0.45) |
| Other Substance Use | Postpartum | 2007 | 0.41 | (0.29 - 0.54) |
| Other Substance Use | Postpartum | 2008 | 0.55 | (0.4 - 0.69) |
| Other Substance Use | Postpartum | 2009 | 0.73 | (0.56 - 0.9) |
| Other Substance Use | Postpartum | 2010 | 0.68 | (0.52 - 0.84) |
| Other Substance Use | Postpartum | 2011 | 0.84 | (0.67 - 1.02) |
| Other Substance Use | Postpartum | 2012 | 1.11 | (0.91 - 1.31) |
| Other Substance Use | Postpartum | 2013 | 1.15 | (0.94 - 1.35) |
| Other Substance Use | Postpartum | 2014 | 1.14 | (0.94 - 1.35) |
| Other Substance Use | Postpartum | 2015 | 0.83 | (0.66 - 1.01) |
| Other Substance Use | Postpartum | 2016 | 1.01 | (0.82 - 1.21) |
| Other Substance Use | Postpartum | 2017 | 1.00 | (0.81 - 1.19) |
| Other Substance Use | Postpartum | 2018 | 1.05 | (0.85 - 1.25) |
| Other Substance Use | Postpartum | 2019 | 0.83 | (0.65 - 1.01) |
| Psychosis | Preconception | 2003 | 0.19 | (0.11 - 0.27) |
| Psychosis | Preconception | 2004 | 0.18 | (0.09 - 0.27) |
| Psychosis | Preconception | 2005 | 0.31 | (0.19 - 0.44) |
| Psychosis | Preconception | 2006 | 0.27 | (0.16 - 0.38) |
| Psychosis | Preconception | 2007 | 0.27 | (0.16 - 0.38) |
| Psychosis | Preconception | 2008 | 0.21 | (0.11 - 0.31) |
| Psychosis | Preconception | 2009 | 0.31 | (0.19 - 0.42) |
| Psychosis | Preconception | 2010 | 0.27 | (0.16 - 0.38) |
| Psychosis | Preconception | 2011 | 0.30 | (0.18 - 0.41) |
| Psychosis | Preconception | 2012 | 0.20 | (0.11 - 0.3) |
| Psychosis | Preconception | 2013 | 0.32 | (0.2 - 0.44) |
| Psychosis | Preconception | 2014 | 0.24 | (0.14 - 0.34) |
| Psychosis | Preconception | 2015 | 0.17 | (0.08 - 0.25) |
| Psychosis | Preconception | 2016 | 0.25 | (0.15 - 0.35) |
| Psychosis | Preconception | 2017 | 0.34 | (0.21 - 0.46) |
| Psychosis | Preconception | 2018 | 0.29 | (0.17 - 0.4) |
| Psychosis | Antepartum | 2003 | 0.12 | (0 - 0.26) |
| Psychosis | Antepartum | 2004 | 0.18 | (0.08 - 0.28) |
| Psychosis | Antepartum | 2005 | 0.15 | (0.06 - 0.23) |
| Psychosis | Antepartum | 2006 | 0.13 | (0.05 - 0.21) |
| Psychosis | Antepartum | 2007 | 0.17 | (0.08 - 0.26) |
| Psychosis | Antepartum | 2008 | 0.13 | (0.05 - 0.21) |
| Psychosis | Antepartum | 2009 | 0.13 | (0.05 - 0.21) |
| Psychosis | Antepartum | 2010 | 0.25 | (0.14 - 0.35) |
| Psychosis | Antepartum | 2011 | 0.11 | (0.04 - 0.18) |
| Psychosis | Antepartum | 2012 | 0.11 | (0.04 - 0.18) |
| Psychosis | Antepartum | 2013 | 0.18 | (0.09 - 0.28) |
| Psychosis | Antepartum | 2014 | 0.12 | (0.05 - 0.2) |
| Psychosis | Antepartum | 2015 | 0.20 | (0.1 - 0.29) |
| Psychosis | Antepartum | 2016 | 0.18 | (0.09 - 0.26) |
| Psychosis | Antepartum | 2017 | 0.07 | (0.01 - 0.13) |
| Psychosis | Antepartum | 2018 | 0.18 | (0.09 - 0.27) |
| Psychosis | Antepartum | 2019 | 0.16 | (0.07 - 0.25) |
| Psychosis | Postpartum | 2004 | 0.52 | (0.31 - 0.72) |
| Psychosis | Postpartum | 2005 | 0.37 | (0.25 - 0.49) |
| Psychosis | Postpartum | 2006 | 0.46 | (0.32 - 0.59) |
| Psychosis | Postpartum | 2007 | 0.44 | (0.31 - 0.57) |
| Psychosis | Postpartum | 2008 | 0.56 | (0.41 - 0.7) |
| Psychosis | Postpartum | 2009 | 0.47 | (0.33 - 0.6) |
| Psychosis | Postpartum | 2010 | 0.45 | (0.32 - 0.58) |
| Psychosis | Postpartum | 2011 | 0.47 | (0.34 - 0.6) |
| Psychosis | Postpartum | 2012 | 0.55 | (0.41 - 0.69) |
| Psychosis | Postpartum | 2013 | 0.53 | (0.39 - 0.68) |
| Psychosis | Postpartum | 2014 | 0.61 | (0.46 - 0.76) |
| Psychosis | Postpartum | 2015 | 0.66 | (0.5 - 0.81) |
| Psychosis | Postpartum | 2016 | 0.63 | (0.48 - 0.78) |
| Psychosis | Postpartum | 2017 | 0.66 | (0.51 - 0.81) |
| Psychosis | Postpartum | 2018 | 0.79 | (0.62 - 0.96) |
| Psychosis | Postpartum | 2019 | 0.71 | (0.55 - 0.87) |
| Bipolar | Preconception | 2003 | 0.08 | (0.03 - 0.13) |
| Bipolar | Preconception | 2004 | 0.18 | (0.09 - 0.27) |
| Bipolar | Preconception | 2005 | 0.27 | (0.15 - 0.38) |
| Bipolar | Preconception | 2006 | 0.35 | (0.22 - 0.48) |
| Bipolar | Preconception | 2007 | 0.54 | (0.38 - 0.7) |
| Bipolar | Preconception | 2008 | 0.80 | (0.6 - 0.99) |
| Bipolar | Preconception | 2009 | 0.92 | (0.71 - 1.12) |
| Bipolar | Preconception | 2010 | 0.88 | (0.69 - 1.08) |
| Bipolar | Preconception | 2011 | 0.99 | (0.78 - 1.2) |
| Bipolar | Preconception | 2012 | 0.85 | (0.66 - 1.05) |
| Bipolar | Preconception | 2013 | 1.05 | (0.83 - 1.26) |
| Bipolar | Preconception | 2014 | 1.23 | (1 - 1.47) |
| Bipolar | Preconception | 2015 | 1.05 | (0.83 - 1.26) |
| Bipolar | Preconception | 2016 | 1.26 | (1.02 - 1.49) |
| Bipolar | Preconception | 2017 | 1.09 | (0.87 - 1.32) |
| Bipolar | Preconception | 2018 | 1.32 | (1.07 - 1.56) |
| Bipolar | Antepartum | 2003 | 0.11 | (0 - 0.23) |
| Bipolar | Antepartum | 2004 | 0.10 | (0.03 - 0.17) |
| Bipolar | Antepartum | 2005 | 0.06 | (0 - 0.11) |
| Bipolar | Antepartum | 2006 | 0.14 | (0.05 - 0.23) |
| Bipolar | Antepartum | 2007 | 0.32 | (0.19 - 0.45) |
| Bipolar | Antepartum | 2008 | 0.39 | (0.25 - 0.53) |
| Bipolar | Antepartum | 2009 | 0.59 | (0.42 - 0.76) |
| Bipolar | Antepartum | 2010 | 0.58 | (0.42 - 0.75) |
| Bipolar | Antepartum | 2011 | 0.52 | (0.36 - 0.68) |
| Bipolar | Antepartum | 2012 | 0.57 | (0.4 - 0.73) |
| Bipolar | Antepartum | 2013 | 0.48 | (0.33 - 0.63) |
| Bipolar | Antepartum | 2014 | 0.60 | (0.43 - 0.77) |
| Bipolar | Antepartum | 2015 | 0.45 | (0.31 - 0.6) |
| Bipolar | Antepartum | 2016 | 0.87 | (0.67 - 1.07) |
| Bipolar | Antepartum | 2017 | 0.45 | (0.31 - 0.59) |
| Bipolar | Antepartum | 2018 | 0.59 | (0.43 - 0.76) |
| Bipolar | Antepartum | 2019 | 0.56 | (0.4 - 0.72) |
| Bipolar | Postpartum | 2004 | 0.23 | (0.09 - 0.37) |
| Bipolar | Postpartum | 2005 | 0.27 | (0.16 - 0.37) |
| Bipolar | Postpartum | 2006 | 0.27 | (0.16 - 0.37) |
| Bipolar | Postpartum | 2007 | 0.44 | (0.31 - 0.57) |
| Bipolar | Postpartum | 2008 | 0.52 | (0.38 - 0.66) |
| Bipolar | Postpartum | 2009 | 0.58 | (0.43 - 0.73) |
| Bipolar | Postpartum | 2010 | 0.68 | (0.52 - 0.84) |
| Bipolar | Postpartum | 2011 | 0.62 | (0.47 - 0.77) |
| Bipolar | Postpartum | 2012 | 0.73 | (0.57 - 0.89) |
| Bipolar | Postpartum | 2013 | 0.73 | (0.56 - 0.89) |
| Bipolar | Postpartum | 2014 | 0.78 | (0.61 - 0.95) |
| Bipolar | Postpartum | 2015 | 0.82 | (0.65 - 0.99) |
| Bipolar | Postpartum | 2016 | 0.81 | (0.64 - 0.99) |
| Bipolar | Postpartum | 2017 | 0.60 | (0.46 - 0.75) |
| Bipolar | Postpartum | 2018 | 0.79 | (0.62 - 0.96) |
| Bipolar | Postpartum | 2019 | 0.59 | (0.44 - 0.74) |
| Stress-Related | Preconception | 2003 | 2.71 | (2.41 - 3.02) |
| Stress-Related | Preconception | 2004 | 5.17 | (4.69 - 5.66) |
| Stress-Related | Preconception | 2005 | 9.41 | (8.73 - 10.09) |
| Stress-Related | Preconception | 2006 | 8.77 | (8.11 - 9.42) |
| Stress-Related | Preconception | 2007 | 10.16 | (9.46 - 10.86) |
| Stress-Related | Preconception | 2008 | 10.00 | (9.32 - 10.69) |
| Stress-Related | Preconception | 2009 | 10.59 | (9.89 - 11.3) |
| Stress-Related | Preconception | 2010 | 11.19 | (10.47 - 11.9) |
| Stress-Related | Preconception | 2011 | 12.67 | (11.9 - 13.44) |
| Stress-Related | Preconception | 2012 | 13.93 | (13.12 - 14.74) |
| Stress-Related | Preconception | 2013 | 15.17 | (14.32 - 16.02) |
| Stress-Related | Preconception | 2014 | 16.11 | (15.24 - 16.99) |
| Stress-Related | Preconception | 2015 | 18.33 | (17.4 - 19.26) |
| Stress-Related | Preconception | 2016 | 21.70 | (20.69 - 22.72) |
| Stress-Related | Preconception | 2017 | 22.92 | (21.86 - 23.98) |
| Stress-Related | Preconception | 2018 | 23.32 | (22.26 - 24.39) |
| Stress-Related | Antepartum | 2003 | 1.65 | (1.17 - 2.13) |
| Stress-Related | Antepartum | 2004 | 3.38 | (2.95 - 3.81) |
| Stress-Related | Antepartum | 2005 | 5.10 | (4.57 - 5.62) |
| Stress-Related | Antepartum | 2006 | 5.01 | (4.5 - 5.53) |
| Stress-Related | Antepartum | 2007 | 6.62 | (6.03 - 7.2) |
| Stress-Related | Antepartum | 2008 | 5.70 | (5.16 - 6.24) |
| Stress-Related | Antepartum | 2009 | 5.80 | (5.25 - 6.34) |
| Stress-Related | Antepartum | 2010 | 5.65 | (5.12 - 6.18) |
| Stress-Related | Antepartum | 2011 | 7.31 | (6.71 - 7.91) |
| Stress-Related | Antepartum | 2012 | 8.18 | (7.54 - 8.83) |
| Stress-Related | Antepartum | 2013 | 8.87 | (8.2 - 9.55) |
| Stress-Related | Antepartum | 2014 | 10.56 | (9.83 - 11.3) |
| Stress-Related | Antepartum | 2015 | 11.60 | (10.84 - 12.37) |
| Stress-Related | Antepartum | 2016 | 13.06 | (12.25 - 13.87) |
| Stress-Related | Antepartum | 2017 | 13.60 | (12.77 - 14.44) |
| Stress-Related | Antepartum | 2018 | 14.00 | (13.15 - 14.86) |
| Stress-Related | Antepartum | 2019 | 14.12 | (13.26 - 14.98) |
| Stress-Related | Postpartum | 2004 | 2.95 | (2.46 - 3.45) |
| Stress-Related | Postpartum | 2005 | 4.97 | (4.51 - 5.42) |
| Stress-Related | Postpartum | 2006 | 4.84 | (4.39 - 5.29) |
| Stress-Related | Postpartum | 2007 | 5.49 | (5.01 - 5.96) |
| Stress-Related | Postpartum | 2008 | 5.76 | (5.28 - 6.24) |
| Stress-Related | Postpartum | 2009 | 6.22 | (5.72 - 6.72) |
| Stress-Related | Postpartum | 2010 | 6.59 | (6.08 - 7.1) |
| Stress-Related | Postpartum | 2011 | 8.08 | (7.53 - 8.64) |
| Stress-Related | Postpartum | 2012 | 7.93 | (7.37 - 8.48) |
| Stress-Related | Postpartum | 2013 | 8.54 | (7.96 - 9.12) |
| Stress-Related | Postpartum | 2014 | 9.43 | (8.82 - 10.04) |
| Stress-Related | Postpartum | 2015 | 10.74 | (10.09 - 11.4) |
| Stress-Related | Postpartum | 2016 | 11.32 | (10.64 - 11.99) |
| Stress-Related | Postpartum | 2017 | 11.38 | (10.71 - 12.06) |
| Stress-Related | Postpartum | 2018 | 11.06 | (10.4 - 11.73) |
| Stress-Related | Postpartum | 2019 | 11.94 | (11.24 - 12.64) |

SIR = standardized incidence rate per 1,000 person-years; CI = confidence intervals

**Supplementary Table 3.** Standardized incidence rate and 95% confidence intervals of psychiatric disorders before, during, and after pregnancy by week.

| **Psychiatric disorder** | **Phase** | **Week** | **SIR** | **95% CI** |
| --- | --- | --- | --- | --- |
| Any | Preconception | 0 | 29.33 | (27 - 31.65) |
| Any | Preconception | 1 | 26.53 | (24.32 - 28.74) |
| Any | Preconception | 2 | 24.42 | (22.31 - 26.54) |
| Any | Preconception | 3 | 27.67 | (25.43 - 29.92) |
| Any | Preconception | 4 | 26.65 | (24.45 - 28.85) |
| Any | Preconception | 5 | 26.64 | (24.44 - 28.84) |
| Any | Preconception | 6 | 28.02 | (25.77 - 30.27) |
| Any | Preconception | 7 | 26.92 | (24.72 - 29.12) |
| Any | Preconception | 8 | 25.36 | (23.22 - 27.49) |
| Any | Preconception | 9 | 27.58 | (25.36 - 29.8) |
| Any | Preconception | 10 | 26.54 | (24.37 - 28.71) |
| Any | Preconception | 11 | 25.78 | (23.65 - 27.92) |
| Any | Preconception | 12 | 24.14 | (22.07 - 26.2) |
| Any | Preconception | 13 | 25.00 | (22.91 - 27.1) |
| Any | Preconception | 14 | 25.86 | (23.73 - 27.99) |
| Any | Preconception | 15 | 27.03 | (24.86 - 29.2) |
| Any | Preconception | 16 | 26.58 | (24.43 - 28.73) |
| Any | Preconception | 17 | 26.46 | (24.32 - 28.6) |
| Any | Preconception | 18 | 26.70 | (24.55 - 28.84) |
| Any | Preconception | 19 | 24.71 | (22.65 - 26.77) |
| Any | Preconception | 20 | 26.30 | (24.17 - 28.42) |
| Any | Preconception | 21 | 26.51 | (24.38 - 28.63) |
| Any | Preconception | 22 | 26.89 | (24.75 - 29.02) |
| Any | Preconception | 23 | 25.43 | (23.35 - 27.5) |
| Any | Preconception | 24 | 27.70 | (25.54 - 29.86) |
| Any | Preconception | 25 | 25.93 | (23.85 - 28.02) |
| Any | Preconception | 26 | 26.87 | (24.75 - 28.99) |
| Any | Preconception | 27 | 26.47 | (24.37 - 28.57) |
| Any | Preconception | 28 | 26.62 | (24.51 - 28.72) |
| Any | Preconception | 29 | 26.40 | (24.31 - 28.49) |
| Any | Preconception | 30 | 27.18 | (25.07 - 29.3) |
| Any | Preconception | 31 | 27.18 | (25.07 - 29.29) |
| Any | Preconception | 32 | 25.09 | (23.07 - 27.12) |
| Any | Preconception | 33 | 25.00 | (22.98 - 27.02) |
| Any | Preconception | 34 | 22.57 | (20.66 - 24.48) |
| Any | Preconception | 35 | 26.28 | (24.22 - 28.35) |
| Any | Preconception | 36 | 27.34 | (25.24 - 29.44) |
| Any | Preconception | 37 | 24.26 | (22.29 - 26.23) |
| Any | Preconception | 38 | 27.68 | (25.57 - 29.78) |
| Any | Preconception | 39 | 26.82 | (24.75 - 28.88) |
| Any | Preconception | 40 | 25.83 | (23.81 - 27.85) |
| Any | Preconception | 41 | 25.90 | (23.88 - 27.92) |
| Any | Preconception | 42 | 24.71 | (22.74 - 26.68) |
| Any | Preconception | 43 | 24.00 | (22.06 - 25.94) |
| Any | Preconception | 44 | 25.59 | (23.59 - 27.59) |
| Any | Preconception | 45 | 25.47 | (23.48 - 27.46) |
| Any | Preconception | 46 | 25.30 | (23.32 - 27.28) |
| Any | Preconception | 47 | 26.79 | (24.76 - 28.82) |
| Any | Preconception | 48 | 25.09 | (23.13 - 27.05) |
| Any | Preconception | 49 | 25.51 | (23.53 - 27.48) |
| Any | Preconception | 50 | 23.62 | (21.72 - 25.51) |
| Any | Preconception | 51 | 24.65 | (22.72 - 26.59) |
| Any | Antepartum | 0 | 28.07 | (26.08 - 30.06) |
| Any | Antepartum | 1 | 22.75 | (20.96 - 24.53) |
| Any | Antepartum | 2 | 19.98 | (18.31 - 21.66) |
| Any | Antepartum | 3 | 21.77 | (20.02 - 23.52) |
| Any | Antepartum | 4 | 18.83 | (17.21 - 20.45) |
| Any | Antepartum | 5 | 17.87 | (16.29 - 19.46) |
| Any | Antepartum | 6 | 17.80 | (16.22 - 19.37) |
| Any | Antepartum | 7 | 15.68 | (14.2 - 17.16) |
| Any | Antepartum | 8 | 15.43 | (13.96 - 16.9) |
| Any | Antepartum | 9 | 14.83 | (13.39 - 16.28) |
| Any | Antepartum | 10 | 15.05 | (13.6 - 16.5) |
| Any | Antepartum | 11 | 15.60 | (14.12 - 17.07) |
| Any | Antepartum | 12 | 14.39 | (12.97 - 15.81) |
| Any | Antepartum | 13 | 15.66 | (14.18 - 17.14) |
| Any | Antepartum | 14 | 16.97 | (15.43 - 18.51) |
| Any | Antepartum | 15 | 15.65 | (14.17 - 17.13) |
| Any | Antepartum | 16 | 16.38 | (14.87 - 17.89) |
| Any | Antepartum | 17 | 16.85 | (15.31 - 18.38) |
| Any | Antepartum | 18 | 16.19 | (14.69 - 17.69) |
| Any | Antepartum | 19 | 15.60 | (14.12 - 17.07) |
| Any | Antepartum | 20 | 16.98 | (15.44 - 18.52) |
| Any | Antepartum | 21 | 16.50 | (14.98 - 18.02) |
| Any | Antepartum | 22 | 16.68 | (15.15 - 18.21) |
| Any | Antepartum | 23 | 15.77 | (14.29 - 17.25) |
| Any | Antepartum | 24 | 17.56 | (15.99 - 19.12) |
| Any | Antepartum | 25 | 16.03 | (14.54 - 17.53) |
| Any | Antepartum | 26 | 16.87 | (15.34 - 18.41) |
| Any | Antepartum | 27 | 15.02 | (13.58 - 16.47) |
| Any | Antepartum | 28 | 16.67 | (15.15 - 18.2) |
| Any | Antepartum | 29 | 16.87 | (15.33 - 18.41) |
| Any | Antepartum | 30 | 14.55 | (13.12 - 15.98) |
| Any | Antepartum | 31 | 14.50 | (13.07 - 15.93) |
| Any | Antepartum | 32 | 14.64 | (13.2 - 16.07) |
| Any | Antepartum | 33 | 13.62 | (12.24 - 15.01) |
| Any | Antepartum | 34 | 11.52 | (10.24 - 12.79) |
| Any | Antepartum | 35 | 10.79 | (9.55 - 12.03) |
| Any | Antepartum | 36 | 8.52 | (7.41 - 9.63) |
| Any | Antepartum | 37 | 6.74 | (5.73 - 7.76) |
| Any | Antepartum | 38 | 4.18 | (3.34 - 5.03) |
| Any | Antepartum | 39 | 3.23 | (2.37 - 4.09) |
| Any | Antepartum | 40 | 3.06 | (1.93 - 4.2) |
| Any | Antepartum | 41 | 3.17 | (1.2 - 5.13) |
| Any | Postpartum | 0 | 12.33 | (11.01 - 13.64) |
| Any | Postpartum | 1 | 20.07 | (18.4 - 21.74) |
| Any | Postpartum | 2 | 18.58 | (16.96 - 20.19) |
| Any | Postpartum | 3 | 18.63 | (17.02 - 20.24) |
| Any | Postpartum | 4 | 20.67 | (18.97 - 22.37) |
| Any | Postpartum | 5 | 18.65 | (17.04 - 20.27) |
| Any | Postpartum | 6 | 23.91 | (22.08 - 25.74) |
| Any | Postpartum | 7 | 22.24 | (20.48 - 24) |
| Any | Postpartum | 8 | 25.20 | (23.32 - 27.07) |
| Any | Postpartum | 9 | 22.93 | (21.14 - 24.73) |
| Any | Postpartum | 10 | 22.80 | (21.01 - 24.59) |
| Any | Postpartum | 11 | 23.01 | (21.21 - 24.82) |
| Any | Postpartum | 12 | 23.35 | (21.53 - 25.17) |
| Any | Postpartum | 13 | 22.88 | (21.08 - 24.68) |
| Any | Postpartum | 14 | 21.50 | (19.75 - 23.25) |
| Any | Postpartum | 15 | 20.69 | (18.97 - 22.41) |
| Any | Postpartum | 16 | 19.55 | (17.88 - 21.22) |
| Any | Postpartum | 17 | 20.77 | (19.04 - 22.49) |
| Any | Postpartum | 18 | 22.52 | (20.72 - 24.32) |
| Any | Postpartum | 19 | 20.76 | (19.03 - 22.49) |
| Any | Postpartum | 20 | 18.32 | (16.7 - 19.95) |
| Any | Postpartum | 21 | 19.62 | (17.93 - 21.31) |
| Any | Postpartum | 22 | 20.05 | (18.35 - 21.76) |
| Any | Postpartum | 23 | 19.39 | (17.71 - 21.07) |
| Any | Postpartum | 24 | 20.37 | (18.64 - 22.09) |
| Any | Postpartum | 25 | 19.48 | (17.79 - 21.17) |
| Any | Postpartum | 26 | 21.80 | (20.01 - 23.59) |
| Any | Postpartum | 27 | 20.92 | (19.16 - 22.68) |
| Any | Postpartum | 28 | 19.54 | (17.84 - 21.24) |
| Any | Postpartum | 29 | 19.43 | (17.73 - 21.13) |
| Any | Postpartum | 30 | 17.75 | (16.12 - 19.37) |
| Any | Postpartum | 31 | 18.03 | (16.38 - 19.67) |
| Any | Postpartum | 32 | 21.01 | (19.23 - 22.79) |
| Any | Postpartum | 33 | 18.84 | (17.15 - 20.52) |
| Any | Postpartum | 34 | 19.93 | (18.19 - 21.66) |
| Any | Postpartum | 35 | 19.01 | (17.31 - 20.71) |
| Any | Postpartum | 36 | 19.72 | (17.99 - 21.46) |
| Any | Postpartum | 37 | 19.37 | (17.65 - 21.09) |
| Any | Postpartum | 38 | 19.34 | (17.62 - 21.07) |
| Any | Postpartum | 39 | 20.36 | (18.59 - 22.14) |
| Any | Postpartum | 40 | 20.18 | (18.41 - 21.95) |
| Any | Postpartum | 41 | 19.09 | (17.36 - 20.81) |
| Any | Postpartum | 42 | 18.95 | (17.22 - 20.67) |
| Any | Postpartum | 43 | 21.74 | (19.9 - 23.59) |
| Any | Postpartum | 44 | 18.49 | (16.78 - 20.2) |
| Any | Postpartum | 45 | 20.19 | (18.4 - 21.99) |
| Any | Postpartum | 46 | 21.06 | (19.23 - 22.89) |
| Any | Postpartum | 47 | 21.85 | (19.98 - 23.72) |
| Any | Postpartum | 48 | 21.97 | (20.08 - 23.85) |
| Any | Postpartum | 49 | 21.71 | (19.83 - 23.58) |
| Any | Postpartum | 50 | 20.17 | (18.36 - 21.98) |
| Any | Postpartum | 51 | 21.00 | (19.26 - 22.74) |
| Depression | Preconception | 0 | 13.53 | (12.04 - 15.02) |
| Depression | Preconception | 1 | 10.54 | (9.23 - 11.85) |
| Depression | Preconception | 2 | 10.96 | (9.63 - 12.3) |
| Depression | Preconception | 3 | 12.69 | (11.26 - 14.12) |
| Depression | Preconception | 4 | 13.09 | (11.64 - 14.55) |
| Depression | Preconception | 5 | 11.52 | (10.17 - 12.88) |
| Depression | Preconception | 6 | 12.15 | (10.76 - 13.54) |
| Depression | Preconception | 7 | 11.93 | (10.55 - 13.31) |
| Depression | Preconception | 8 | 11.69 | (10.33 - 13.05) |
| Depression | Preconception | 9 | 12.63 | (11.22 - 14.04) |
| Depression | Preconception | 10 | 12.12 | (10.73 - 13.5) |
| Depression | Preconception | 11 | 11.03 | (9.72 - 12.35) |
| Depression | Preconception | 12 | 9.39 | (8.18 - 10.6) |
| Depression | Preconception | 13 | 11.90 | (10.54 - 13.26) |
| Depression | Preconception | 14 | 11.75 | (10.4 - 13.1) |
| Depression | Preconception | 15 | 11.57 | (10.24 - 12.91) |
| Depression | Preconception | 16 | 10.60 | (9.33 - 11.88) |
| Depression | Preconception | 17 | 11.33 | (10.01 - 12.65) |
| Depression | Preconception | 18 | 12.03 | (10.67 - 13.38) |
| Depression | Preconception | 19 | 11.31 | (10 - 12.62) |
| Depression | Preconception | 20 | 10.92 | (9.63 - 12.2) |
| Depression | Preconception | 21 | 11.10 | (9.81 - 12.39) |
| Depression | Preconception | 22 | 12.11 | (10.76 - 13.46) |
| Depression | Preconception | 23 | 10.70 | (9.44 - 11.97) |
| Depression | Preconception | 24 | 11.04 | (9.76 - 12.33) |
| Depression | Preconception | 25 | 11.31 | (10.01 - 12.6) |
| Depression | Preconception | 26 | 11.26 | (9.97 - 12.55) |
| Depression | Preconception | 27 | 11.33 | (10.04 - 12.62) |
| Depression | Preconception | 28 | 12.12 | (10.79 - 13.46) |
| Depression | Preconception | 29 | 12.23 | (10.89 - 13.56) |
| Depression | Preconception | 30 | 11.87 | (10.56 - 13.18) |
| Depression | Preconception | 31 | 11.15 | (9.88 - 12.42) |
| Depression | Preconception | 32 | 11.73 | (10.43 - 13.03) |
| Depression | Preconception | 33 | 11.13 | (9.87 - 12.4) |
| Depression | Preconception | 34 | 11.20 | (9.93 - 12.46) |
| Depression | Preconception | 35 | 10.14 | (8.94 - 11.34) |
| Depression | Preconception | 36 | 11.88 | (10.59 - 13.18) |
| Depression | Preconception | 37 | 11.18 | (9.92 - 12.43) |
| Depression | Preconception | 38 | 10.14 | (8.95 - 11.34) |
| Depression | Preconception | 39 | 11.13 | (9.88 - 12.38) |
| Depression | Preconception | 40 | 9.68 | (8.52 - 10.84) |
| Depression | Preconception | 41 | 10.51 | (9.3 - 11.72) |
| Depression | Preconception | 42 | 10.72 | (9.5 - 11.94) |
| Depression | Preconception | 43 | 11.03 | (9.8 - 12.26) |
| Depression | Preconception | 44 | 10.52 | (9.32 - 11.72) |
| Depression | Preconception | 45 | 10.55 | (9.35 - 11.74) |
| Depression | Preconception | 46 | 9.18 | (8.06 - 10.3) |
| Depression | Preconception | 47 | 10.44 | (9.25 - 11.63) |
| Depression | Preconception | 48 | 10.19 | (9.01 - 11.36) |
| Depression | Preconception | 49 | 11.18 | (9.96 - 12.41) |
| Depression | Preconception | 50 | 10.71 | (9.52 - 11.91) |
| Depression | Preconception | 51 | 9.98 | (8.83 - 11.14) |
| Depression | Antepartum | 0 | 11.05 | (9.88 - 12.21) |
| Depression | Antepartum | 1 | 9.07 | (8.01 - 10.13) |
| Depression | Antepartum | 2 | 10.08 | (8.97 - 11.2) |
| Depression | Antepartum | 3 | 8.83 | (7.79 - 9.87) |
| Depression | Antepartum | 4 | 7.16 | (6.23 - 8.1) |
| Depression | Antepartum | 5 | 7.00 | (6.07 - 7.93) |
| Depression | Antepartum | 6 | 7.38 | (6.43 - 8.33) |
| Depression | Antepartum | 7 | 6.86 | (5.95 - 7.78) |
| Depression | Antepartum | 8 | 6.16 | (5.29 - 7.03) |
| Depression | Antepartum | 9 | 6.57 | (5.68 - 7.47) |
| Depression | Antepartum | 10 | 7.78 | (6.81 - 8.76) |
| Depression | Antepartum | 11 | 7.18 | (6.24 - 8.11) |
| Depression | Antepartum | 12 | 7.08 | (6.15 - 8.01) |
| Depression | Antepartum | 13 | 7.49 | (6.53 - 8.45) |
| Depression | Antepartum | 14 | 8.83 | (7.79 - 9.87) |
| Depression | Antepartum | 15 | 7.71 | (6.74 - 8.68) |
| Depression | Antepartum | 16 | 7.71 | (6.73 - 8.68) |
| Depression | Antepartum | 17 | 7.70 | (6.73 - 8.67) |
| Depression | Antepartum | 18 | 8.15 | (7.15 - 9.15) |
| Depression | Antepartum | 19 | 7.35 | (6.4 - 8.29) |
| Depression | Antepartum | 20 | 7.44 | (6.49 - 8.39) |
| Depression | Antepartum | 21 | 7.47 | (6.51 - 8.42) |
| Depression | Antepartum | 22 | 8.20 | (7.2 - 9.2) |
| Depression | Antepartum | 23 | 7.02 | (6.1 - 7.95) |
| Depression | Antepartum | 24 | 7.69 | (6.72 - 8.66) |
| Depression | Antepartum | 25 | 8.26 | (7.26 - 9.27) |
| Depression | Antepartum | 26 | 7.15 | (6.22 - 8.09) |
| Depression | Antepartum | 27 | 6.84 | (5.92 - 7.75) |
| Depression | Antepartum | 28 | 6.59 | (5.69 - 7.48) |
| Depression | Antepartum | 29 | 7.26 | (6.32 - 8.2) |
| Depression | Antepartum | 30 | 7.23 | (6.29 - 8.17) |
| Depression | Antepartum | 31 | 6.03 | (5.17 - 6.89) |
| Depression | Antepartum | 32 | 5.75 | (4.91 - 6.6) |
| Depression | Antepartum | 33 | 5.48 | (4.66 - 6.3) |
| Depression | Antepartum | 34 | 4.68 | (3.91 - 5.44) |
| Depression | Antepartum | 35 | 4.46 | (3.71 - 5.2) |
| Depression | Antepartum | 36 | 3.24 | (2.6 - 3.88) |
| Depression | Antepartum | 37 | 2.31 | (1.76 - 2.87) |
| Depression | Antepartum | 38 | 2.14 | (1.58 - 2.71) |
| Depression | Antepartum | 39 | 1.53 | (0.97 - 2.09) |
| Depression | Antepartum | 40 | 0.87 | (0.3 - 1.43) |
| Depression | Antepartum | 41 | 0.85 | (0 - 1.81) |
| Depression | Postpartum | 0 | 4.56 | (3.82 - 5.31) |
| Depression | Postpartum | 1 | 9.85 | (8.76 - 10.94) |
| Depression | Postpartum | 2 | 10.85 | (9.7 - 11.99) |
| Depression | Postpartum | 3 | 11.96 | (10.75 - 13.16) |
| Depression | Postpartum | 4 | 12.56 | (11.32 - 13.79) |
| Depression | Postpartum | 5 | 11.88 | (10.67 - 13.08) |
| Depression | Postpartum | 6 | 13.31 | (12.04 - 14.59) |
| Depression | Postpartum | 7 | 14.18 | (12.86 - 15.49) |
| Depression | Postpartum | 8 | 15.01 | (13.66 - 16.36) |
| Depression | Postpartum | 9 | 12.70 | (11.46 - 13.95) |
| Depression | Postpartum | 10 | 13.51 | (12.22 - 14.8) |
| Depression | Postpartum | 11 | 13.67 | (12.38 - 14.97) |
| Depression | Postpartum | 12 | 13.70 | (12.4 - 15) |
| Depression | Postpartum | 13 | 13.59 | (12.29 - 14.88) |
| Depression | Postpartum | 14 | 11.98 | (10.76 - 13.2) |
| Depression | Postpartum | 15 | 12.21 | (10.98 - 13.45) |
| Depression | Postpartum | 16 | 11.20 | (10.02 - 12.39) |
| Depression | Postpartum | 17 | 12.06 | (10.83 - 13.29) |
| Depression | Postpartum | 18 | 11.52 | (10.32 - 12.72) |
| Depression | Postpartum | 19 | 10.36 | (9.22 - 11.51) |
| Depression | Postpartum | 20 | 9.74 | (8.63 - 10.85) |
| Depression | Postpartum | 21 | 9.76 | (8.65 - 10.87) |
| Depression | Postpartum | 22 | 10.35 | (9.21 - 11.5) |
| Depression | Postpartum | 23 | 9.32 | (8.23 - 10.41) |
| Depression | Postpartum | 24 | 10.91 | (9.73 - 12.09) |
| Depression | Postpartum | 25 | 9.88 | (8.75 - 11) |
| Depression | Postpartum | 26 | 9.97 | (8.84 - 11.1) |
| Depression | Postpartum | 27 | 10.41 | (9.25 - 11.57) |
| Depression | Postpartum | 28 | 11.00 | (9.81 - 12.19) |
| Depression | Postpartum | 29 | 10.38 | (9.22 - 11.53) |
| Depression | Postpartum | 30 | 9.77 | (8.65 - 10.9) |
| Depression | Postpartum | 31 | 10.74 | (9.56 - 11.92) |
| Depression | Postpartum | 32 | 10.44 | (9.27 - 11.61) |
| Depression | Postpartum | 33 | 10.40 | (9.24 - 11.57) |
| Depression | Postpartum | 34 | 10.50 | (9.33 - 11.68) |
| Depression | Postpartum | 35 | 9.91 | (8.77 - 11.06) |
| Depression | Postpartum | 36 | 10.40 | (9.22 - 11.57) |
| Depression | Postpartum | 37 | 10.84 | (9.64 - 12.04) |
| Depression | Postpartum | 38 | 10.31 | (9.14 - 11.49) |
| Depression | Postpartum | 39 | 9.87 | (8.72 - 11.02) |
| Depression | Postpartum | 40 | 9.99 | (8.83 - 11.15) |
| Depression | Postpartum | 41 | 10.46 | (9.27 - 11.65) |
| Depression | Postpartum | 42 | 10.00 | (8.84 - 11.17) |
| Depression | Postpartum | 43 | 10.49 | (9.29 - 11.68) |
| Depression | Postpartum | 44 | 9.53 | (8.38 - 10.67) |
| Depression | Postpartum | 45 | 9.82 | (8.66 - 10.99) |
| Depression | Postpartum | 46 | 9.79 | (8.62 - 10.96) |
| Depression | Postpartum | 47 | 9.44 | (8.29 - 10.58) |
| Depression | Postpartum | 48 | 10.37 | (9.17 - 11.58) |
| Depression | Postpartum | 49 | 10.26 | (9.06 - 11.46) |
| Depression | Postpartum | 50 | 9.56 | (8.39 - 10.72) |
| Depression | Postpartum | 51 | 10.09 | (8.97 - 11.21) |
| Anxiety | Preconception | 0 | 14.58 | (13.03 - 16.12) |
| Anxiety | Preconception | 1 | 12.61 | (11.18 - 14.05) |
| Anxiety | Preconception | 2 | 11.52 | (10.15 - 12.89) |
| Anxiety | Preconception | 3 | 13.45 | (11.97 - 14.92) |
| Anxiety | Preconception | 4 | 13.30 | (11.84 - 14.77) |
| Anxiety | Preconception | 5 | 13.23 | (11.77 - 14.69) |
| Anxiety | Preconception | 6 | 12.13 | (10.74 - 13.52) |
| Anxiety | Preconception | 7 | 13.00 | (11.56 - 14.44) |
| Anxiety | Preconception | 8 | 13.16 | (11.72 - 14.6) |
| Anxiety | Preconception | 9 | 13.20 | (11.76 - 14.64) |
| Anxiety | Preconception | 10 | 11.98 | (10.61 - 13.35) |
| Anxiety | Preconception | 11 | 13.63 | (12.17 - 15.09) |
| Anxiety | Preconception | 12 | 11.77 | (10.41 - 13.12) |
| Anxiety | Preconception | 13 | 12.93 | (11.51 - 14.34) |
| Anxiety | Preconception | 14 | 12.12 | (10.75 - 13.49) |
| Anxiety | Preconception | 15 | 12.52 | (11.13 - 13.9) |
| Anxiety | Preconception | 16 | 13.83 | (12.37 - 15.29) |
| Anxiety | Preconception | 17 | 12.82 | (11.42 - 14.22) |
| Anxiety | Preconception | 18 | 12.52 | (11.14 - 13.9) |
| Anxiety | Preconception | 19 | 12.87 | (11.47 - 14.27) |
| Anxiety | Preconception | 20 | 13.40 | (11.98 - 14.82) |
| Anxiety | Preconception | 21 | 13.11 | (11.71 - 14.52) |
| Anxiety | Preconception | 22 | 13.40 | (11.99 - 14.82) |
| Anxiety | Preconception | 23 | 12.81 | (11.43 - 14.2) |
| Anxiety | Preconception | 24 | 12.91 | (11.53 - 14.3) |
| Anxiety | Preconception | 25 | 12.55 | (11.19 - 13.91) |
| Anxiety | Preconception | 26 | 13.11 | (11.72 - 14.5) |
| Anxiety | Preconception | 27 | 12.75 | (11.38 - 14.12) |
| Anxiety | Preconception | 28 | 12.96 | (11.59 - 14.34) |
| Anxiety | Preconception | 29 | 12.87 | (11.5 - 14.24) |
| Anxiety | Preconception | 30 | 12.93 | (11.56 - 14.3) |
| Anxiety | Preconception | 31 | 14.84 | (13.37 - 16.3) |
| Anxiety | Preconception | 32 | 13.23 | (11.86 - 14.61) |
| Anxiety | Preconception | 33 | 12.40 | (11.07 - 13.73) |
| Anxiety | Preconception | 34 | 11.38 | (10.1 - 12.65) |
| Anxiety | Preconception | 35 | 11.96 | (10.66 - 13.27) |
| Anxiety | Preconception | 36 | 12.31 | (11 - 13.63) |
| Anxiety | Preconception | 37 | 12.35 | (11.03 - 13.67) |
| Anxiety | Preconception | 38 | 12.88 | (11.54 - 14.23) |
| Anxiety | Preconception | 39 | 13.28 | (11.92 - 14.65) |
| Anxiety | Preconception | 40 | 11.86 | (10.58 - 13.14) |
| Anxiety | Preconception | 41 | 11.88 | (10.6 - 13.16) |
| Anxiety | Preconception | 42 | 12.39 | (11.09 - 13.7) |
| Anxiety | Preconception | 43 | 12.84 | (11.51 - 14.16) |
| Anxiety | Preconception | 44 | 12.45 | (11.14 - 13.75) |
| Anxiety | Preconception | 45 | 10.94 | (9.72 - 12.17) |
| Anxiety | Preconception | 46 | 12.17 | (10.89 - 13.45) |
| Anxiety | Preconception | 47 | 13.35 | (12.01 - 14.69) |
| Anxiety | Preconception | 48 | 14.06 | (12.69 - 15.43) |
| Anxiety | Preconception | 49 | 10.73 | (9.53 - 11.92) |
| Anxiety | Preconception | 50 | 10.81 | (9.62 - 12.01) |
| Anxiety | Preconception | 51 | 11.75 | (10.5 - 12.99) |
| Anxiety | Antepartum | 0 | 13.19 | (11.92 - 14.47) |
| Anxiety | Antepartum | 1 | 10.91 | (9.75 - 12.07) |
| Anxiety | Antepartum | 2 | 10.67 | (9.53 - 11.81) |
| Anxiety | Antepartum | 3 | 10.86 | (9.7 - 12.01) |
| Anxiety | Antepartum | 4 | 9.89 | (8.79 - 10.99) |
| Anxiety | Antepartum | 5 | 9.25 | (8.19 - 10.32) |
| Anxiety | Antepartum | 6 | 9.69 | (8.6 - 10.78) |
| Anxiety | Antepartum | 7 | 7.94 | (6.95 - 8.92) |
| Anxiety | Antepartum | 8 | 8.06 | (7.07 - 9.06) |
| Anxiety | Antepartum | 9 | 8.16 | (7.16 - 9.16) |
| Anxiety | Antepartum | 10 | 6.62 | (5.72 - 7.53) |
| Anxiety | Antepartum | 11 | 6.81 | (5.9 - 7.72) |
| Anxiety | Antepartum | 12 | 6.91 | (5.99 - 7.83) |
| Anxiety | Antepartum | 13 | 7.32 | (6.37 - 8.27) |
| Anxiety | Antepartum | 14 | 7.76 | (6.78 - 8.73) |
| Anxiety | Antepartum | 15 | 7.06 | (6.13 - 7.99) |
| Anxiety | Antepartum | 16 | 7.69 | (6.72 - 8.66) |
| Anxiety | Antepartum | 17 | 6.96 | (6.03 - 7.88) |
| Anxiety | Antepartum | 18 | 7.53 | (6.57 - 8.49) |
| Anxiety | Antepartum | 19 | 7.18 | (6.24 - 8.11) |
| Anxiety | Antepartum | 20 | 8.22 | (7.22 - 9.22) |
| Anxiety | Antepartum | 21 | 7.68 | (6.71 - 8.64) |
| Anxiety | Antepartum | 22 | 8.44 | (7.42 - 9.45) |
| Anxiety | Antepartum | 23 | 7.64 | (6.68 - 8.61) |
| Anxiety | Antepartum | 24 | 7.71 | (6.74 - 8.67) |
| Anxiety | Antepartum | 25 | 7.62 | (6.65 - 8.58) |
| Anxiety | Antepartum | 26 | 7.42 | (6.47 - 8.38) |
| Anxiety | Antepartum | 27 | 7.21 | (6.27 - 8.14) |
| Anxiety | Antepartum | 28 | 6.67 | (5.77 - 7.57) |
| Anxiety | Antepartum | 29 | 7.24 | (6.3 - 8.18) |
| Anxiety | Antepartum | 30 | 6.30 | (5.42 - 7.18) |
| Anxiety | Antepartum | 31 | 6.43 | (5.55 - 7.32) |
| Anxiety | Antepartum | 32 | 6.96 | (6.03 - 7.88) |
| Anxiety | Antepartum | 33 | 5.99 | (5.13 - 6.85) |
| Anxiety | Antepartum | 34 | 4.86 | (4.09 - 5.64) |
| Anxiety | Antepartum | 35 | 4.55 | (3.79 - 5.3) |
| Anxiety | Antepartum | 36 | 4.63 | (3.86 - 5.39) |
| Anxiety | Antepartum | 37 | 3.48 | (2.8 - 4.16) |
| Anxiety | Antepartum | 38 | 2.57 | (1.95 - 3.19) |
| Anxiety | Antepartum | 39 | 1.25 | (0.75 - 1.76) |
| Anxiety | Antepartum | 40 | 1.54 | (0.78 - 2.29) |
| Anxiety | Antepartum | 41 | 1.70 | (0.34 - 3.06) |
| Anxiety | Postpartum | 0 | 4.99 | (4.22 - 5.77) |
| Anxiety | Postpartum | 1 | 7.23 | (6.29 - 8.16) |
| Anxiety | Postpartum | 2 | 6.40 | (5.52 - 7.28) |
| Anxiety | Postpartum | 3 | 6.52 | (5.63 - 7.4) |
| Anxiety | Postpartum | 4 | 7.64 | (6.69 - 8.6) |
| Anxiety | Postpartum | 5 | 7.23 | (6.3 - 8.17) |
| Anxiety | Postpartum | 6 | 9.19 | (8.14 - 10.25) |
| Anxiety | Postpartum | 7 | 9.07 | (8.02 - 10.11) |
| Anxiety | Postpartum | 8 | 10.43 | (9.31 - 11.55) |
| Anxiety | Postpartum | 9 | 10.93 | (9.78 - 12.08) |
| Anxiety | Postpartum | 10 | 11.23 | (10.06 - 12.4) |
| Anxiety | Postpartum | 11 | 10.82 | (9.67 - 11.97) |
| Anxiety | Postpartum | 12 | 11.04 | (9.87 - 12.2) |
| Anxiety | Postpartum | 13 | 10.07 | (8.96 - 11.18) |
| Anxiety | Postpartum | 14 | 10.65 | (9.5 - 11.79) |
| Anxiety | Postpartum | 15 | 10.31 | (9.19 - 11.44) |
| Anxiety | Postpartum | 16 | 10.29 | (9.16 - 11.41) |
| Anxiety | Postpartum | 17 | 10.18 | (9.06 - 11.3) |
| Anxiety | Postpartum | 18 | 10.13 | (9 - 11.25) |
| Anxiety | Postpartum | 19 | 9.24 | (8.17 - 10.32) |
| Anxiety | Postpartum | 20 | 10.90 | (9.73 - 12.06) |
| Anxiety | Postpartum | 21 | 9.97 | (8.85 - 11.09) |
| Anxiety | Postpartum | 22 | 9.84 | (8.72 - 10.95) |
| Anxiety | Postpartum | 23 | 8.42 | (7.39 - 9.46) |
| Anxiety | Postpartum | 24 | 10.03 | (8.91 - 11.16) |
| Anxiety | Postpartum | 25 | 9.90 | (8.78 - 11.02) |
| Anxiety | Postpartum | 26 | 10.04 | (8.91 - 11.17) |
| Anxiety | Postpartum | 27 | 9.44 | (8.34 - 10.54) |
| Anxiety | Postpartum | 28 | 10.94 | (9.75 - 12.12) |
| Anxiety | Postpartum | 29 | 9.59 | (8.47 - 10.7) |
| Anxiety | Postpartum | 30 | 9.67 | (8.56 - 10.79) |
| Anxiety | Postpartum | 31 | 9.61 | (8.49 - 10.73) |
| Anxiety | Postpartum | 32 | 9.95 | (8.81 - 11.09) |
| Anxiety | Postpartum | 33 | 11.31 | (10.09 - 12.53) |
| Anxiety | Postpartum | 34 | 9.79 | (8.65 - 10.92) |
| Anxiety | Postpartum | 35 | 9.68 | (8.55 - 10.81) |
| Anxiety | Postpartum | 36 | 10.37 | (9.2 - 11.55) |
| Anxiety | Postpartum | 37 | 10.21 | (9.04 - 11.38) |
| Anxiety | Postpartum | 38 | 10.50 | (9.31 - 11.69) |
| Anxiety | Postpartum | 39 | 10.27 | (9.1 - 11.45) |
| Anxiety | Postpartum | 40 | 11.41 | (10.17 - 12.66) |
| Anxiety | Postpartum | 41 | 10.33 | (9.15 - 11.52) |
| Anxiety | Postpartum | 42 | 9.62 | (8.47 - 10.76) |
| Anxiety | Postpartum | 43 | 12.11 | (10.82 - 13.4) |
| Anxiety | Postpartum | 44 | 10.42 | (9.22 - 11.62) |
| Anxiety | Postpartum | 45 | 10.99 | (9.75 - 12.22) |
| Anxiety | Postpartum | 46 | 11.42 | (10.16 - 12.69) |
| Anxiety | Postpartum | 47 | 11.74 | (10.46 - 13.02) |
| Anxiety | Postpartum | 48 | 11.79 | (10.51 - 13.08) |
| Anxiety | Postpartum | 49 | 11.54 | (10.27 - 12.82) |
| Anxiety | Postpartum | 50 | 10.17 | (8.97 - 11.38) |
| Anxiety | Postpartum | 51 | 11.13 | (9.95 - 12.32) |
| Alcohol Use | Preconception | 0 | 1.91 | (1.37 - 2.44) |
| Alcohol Use | Preconception | 1 | 1.79 | (1.27 - 2.31) |
| Alcohol Use | Preconception | 2 | 2.02 | (1.47 - 2.58) |
| Alcohol Use | Preconception | 3 | 1.39 | (0.93 - 1.84) |
| Alcohol Use | Preconception | 4 | 1.66 | (1.15 - 2.16) |
| Alcohol Use | Preconception | 5 | 1.06 | (0.66 - 1.45) |
| Alcohol Use | Preconception | 6 | 1.01 | (0.62 - 1.4) |
| Alcohol Use | Preconception | 7 | 1.83 | (1.31 - 2.35) |
| Alcohol Use | Preconception | 8 | 1.67 | (1.17 - 2.16) |
| Alcohol Use | Preconception | 9 | 1.86 | (1.33 - 2.38) |
| Alcohol Use | Preconception | 10 | 1.50 | (1.03 - 1.98) |
| Alcohol Use | Preconception | 11 | 1.57 | (1.09 - 2.05) |
| Alcohol Use | Preconception | 12 | 1.83 | (1.31 - 2.35) |
| Alcohol Use | Preconception | 13 | 2.02 | (1.48 - 2.57) |
| Alcohol Use | Preconception | 14 | 1.82 | (1.3 - 2.33) |
| Alcohol Use | Preconception | 15 | 1.55 | (1.08 - 2.03) |
| Alcohol Use | Preconception | 16 | 1.43 | (0.98 - 1.89) |
| Alcohol Use | Preconception | 17 | 1.58 | (1.1 - 2.06) |
| Alcohol Use | Preconception | 18 | 1.39 | (0.94 - 1.83) |
| Alcohol Use | Preconception | 19 | 1.80 | (1.29 - 2.3) |
| Alcohol Use | Preconception | 20 | 1.41 | (0.96 - 1.86) |
| Alcohol Use | Preconception | 21 | 1.48 | (1.02 - 1.94) |
| Alcohol Use | Preconception | 22 | 1.11 | (0.71 - 1.5) |
| Alcohol Use | Preconception | 23 | 1.25 | (0.83 - 1.67) |
| Alcohol Use | Preconception | 24 | 1.76 | (1.26 - 2.26) |
| Alcohol Use | Preconception | 25 | 1.57 | (1.1 - 2.04) |
| Alcohol Use | Preconception | 26 | 1.31 | (0.88 - 1.74) |
| Alcohol Use | Preconception | 27 | 1.34 | (0.91 - 1.77) |
| Alcohol Use | Preconception | 28 | 1.59 | (1.12 - 2.06) |
| Alcohol Use | Preconception | 29 | 1.22 | (0.81 - 1.64) |
| Alcohol Use | Preconception | 30 | 1.86 | (1.36 - 2.37) |
| Alcohol Use | Preconception | 31 | 1.64 | (1.17 - 2.12) |
| Alcohol Use | Preconception | 32 | 1.74 | (1.25 - 2.23) |
| Alcohol Use | Preconception | 33 | 1.20 | (0.8 - 1.61) |
| Alcohol Use | Preconception | 34 | 0.89 | (0.54 - 1.23) |
| Alcohol Use | Preconception | 35 | 1.30 | (0.88 - 1.72) |
| Alcohol Use | Preconception | 36 | 1.43 | (0.99 - 1.87) |
| Alcohol Use | Preconception | 37 | 1.29 | (0.87 - 1.7) |
| Alcohol Use | Preconception | 38 | 1.42 | (0.99 - 1.86) |
| Alcohol Use | Preconception | 39 | 1.52 | (1.07 - 1.96) |
| Alcohol Use | Preconception | 40 | 1.49 | (1.04 - 1.93) |
| Alcohol Use | Preconception | 41 | 1.52 | (1.07 - 1.97) |
| Alcohol Use | Preconception | 42 | 1.29 | (0.88 - 1.71) |
| Alcohol Use | Preconception | 43 | 1.32 | (0.91 - 1.74) |
| Alcohol Use | Preconception | 44 | 1.42 | (0.99 - 1.85) |
| Alcohol Use | Preconception | 45 | 1.50 | (1.06 - 1.94) |
| Alcohol Use | Preconception | 46 | 1.52 | (1.08 - 1.96) |
| Alcohol Use | Preconception | 47 | 1.27 | (0.87 - 1.67) |
| Alcohol Use | Preconception | 48 | 1.16 | (0.78 - 1.54) |
| Alcohol Use | Preconception | 49 | 1.19 | (0.8 - 1.58) |
| Alcohol Use | Preconception | 50 | 0.99 | (0.63 - 1.34) |
| Alcohol Use | Preconception | 51 | 1.02 | (0.66 - 1.38) |
| Alcohol Use | Antepartum | 0 | 1.55 | (1.13 - 1.98) |
| Alcohol Use | Antepartum | 1 | 0.97 | (0.64 - 1.31) |
| Alcohol Use | Antepartum | 2 | 0.84 | (0.53 - 1.16) |
| Alcohol Use | Antepartum | 3 | 0.55 | (0.29 - 0.8) |
| Alcohol Use | Antepartum | 4 | 0.60 | (0.34 - 0.87) |
| Alcohol Use | Antepartum | 5 | 0.27 | (0.09 - 0.45) |
| Alcohol Use | Antepartum | 6 | 0.36 | (0.16 - 0.56) |
| Alcohol Use | Antepartum | 7 | 0.18 | (0.04 - 0.32) |
| Alcohol Use | Antepartum | 8 | 0.30 | (0.11 - 0.49) |
| Alcohol Use | Antepartum | 9 | 0.18 | (0.04 - 0.32) |
| Alcohol Use | Antepartum | 10 | 0.18 | (0.04 - 0.32) |
| Alcohol Use | Antepartum | 11 | 0.24 | (0.07 - 0.41) |
| Alcohol Use | Antepartum | 12 | 0.27 | (0.09 - 0.45) |
| Alcohol Use | Antepartum | 13 | 0.09 | (0 - 0.19) |
| Alcohol Use | Antepartum | 14 | 0.30 | (0.11 - 0.49) |
| Alcohol Use | Antepartum | 15 | 0.24 | (0.07 - 0.41) |
| Alcohol Use | Antepartum | 16 | 0.18 | (0.04 - 0.32) |
| Alcohol Use | Antepartum | 17 | 0.24 | (0.07 - 0.41) |
| Alcohol Use | Antepartum | 18 | 0.48 | (0.24 - 0.71) |
| Alcohol Use | Antepartum | 19 | 0.27 | (0.09 - 0.44) |
| Alcohol Use | Antepartum | 20 | 0.36 | (0.16 - 0.56) |
| Alcohol Use | Antepartum | 21 | 0.12 | (0 - 0.24) |
| Alcohol Use | Antepartum | 22 | 0.09 | (0 - 0.19) |
| Alcohol Use | Antepartum | 23 | 0.12 | (0 - 0.24) |
| Alcohol Use | Antepartum | 24 | 0.12 | (0 - 0.24) |
| Alcohol Use | Antepartum | 25 | 0.30 | (0.11 - 0.48) |
| Alcohol Use | Antepartum | 26 | 0.15 | (0.02 - 0.28) |
| Alcohol Use | Antepartum | 27 | 0.03 | (0 - 0.09) |
| Alcohol Use | Antepartum | 28 | 0.30 | (0.11 - 0.48) |
| Alcohol Use | Antepartum | 29 | 0.18 | (0.04 - 0.32) |
| Alcohol Use | Antepartum | 30 | 0.09 | (0 - 0.19) |
| Alcohol Use | Antepartum | 31 | 0.06 | (0 - 0.14) |
| Alcohol Use | Antepartum | 32 | 0.12 | (0 - 0.24) |
| Alcohol Use | Antepartum | 33 | 0.12 | (0 - 0.24) |
| Alcohol Use | Antepartum | 34 | 0.00 | (0 - 0) |
| Alcohol Use | Antepartum | 35 | 0.03 | (0 - 0.09) |
| Alcohol Use | Antepartum | 36 | 0.06 | (0 - 0.15) |
| Alcohol Use | Antepartum | 37 | 0.10 | (0 - 0.21) |
| Alcohol Use | Antepartum | 38 | 0.15 | (0 - 0.29) |
| Alcohol Use | Antepartum | 39 | 0.05 | (0 - 0.15) |
| Alcohol Use | Antepartum | 40 | 0.00 | (0 - 0) |
| Alcohol Use | Antepartum | 41 | 0.00 | (0 - 0) |
| Alcohol Use | Postpartum | 0 | 0.03 | (0 - 0.09) |
| Alcohol Use | Postpartum | 1 | 0.03 | (0 - 0.08) |
| Alcohol Use | Postpartum | 2 | 0.06 | (0 - 0.15) |
| Alcohol Use | Postpartum | 3 | 0.14 | (0.02 - 0.26) |
| Alcohol Use | Postpartum | 4 | 0.20 | (0.05 - 0.35) |
| Alcohol Use | Postpartum | 5 | 0.12 | (0 - 0.23) |
| Alcohol Use | Postpartum | 6 | 0.18 | (0.03 - 0.32) |
| Alcohol Use | Postpartum | 7 | 0.23 | (0.07 - 0.4) |
| Alcohol Use | Postpartum | 8 | 0.12 | (0 - 0.24) |
| Alcohol Use | Postpartum | 9 | 0.18 | (0.04 - 0.32) |
| Alcohol Use | Postpartum | 10 | 0.12 | (0 - 0.24) |
| Alcohol Use | Postpartum | 11 | 0.42 | (0.2 - 0.64) |
| Alcohol Use | Postpartum | 12 | 0.21 | (0.05 - 0.36) |
| Alcohol Use | Postpartum | 13 | 0.15 | (0.02 - 0.28) |
| Alcohol Use | Postpartum | 14 | 0.18 | (0.04 - 0.32) |
| Alcohol Use | Postpartum | 15 | 0.24 | (0.07 - 0.41) |
| Alcohol Use | Postpartum | 16 | 0.39 | (0.18 - 0.61) |
| Alcohol Use | Postpartum | 17 | 0.33 | (0.14 - 0.53) |
| Alcohol Use | Postpartum | 18 | 0.18 | (0.04 - 0.33) |
| Alcohol Use | Postpartum | 19 | 0.24 | (0.07 - 0.41) |
| Alcohol Use | Postpartum | 20 | 0.22 | (0.06 - 0.38) |
| Alcohol Use | Postpartum | 21 | 0.21 | (0.06 - 0.37) |
| Alcohol Use | Postpartum | 22 | 0.18 | (0.04 - 0.33) |
| Alcohol Use | Postpartum | 23 | 0.34 | (0.14 - 0.54) |
| Alcohol Use | Postpartum | 24 | 0.40 | (0.18 - 0.62) |
| Alcohol Use | Postpartum | 25 | 0.53 | (0.28 - 0.78) |
| Alcohol Use | Postpartum | 26 | 0.25 | (0.08 - 0.42) |
| Alcohol Use | Postpartum | 27 | 0.25 | (0.08 - 0.42) |
| Alcohol Use | Postpartum | 28 | 0.44 | (0.21 - 0.67) |
| Alcohol Use | Postpartum | 29 | 0.41 | (0.19 - 0.63) |
| Alcohol Use | Postpartum | 30 | 0.35 | (0.14 - 0.55) |
| Alcohol Use | Postpartum | 31 | 0.32 | (0.12 - 0.51) |
| Alcohol Use | Postpartum | 32 | 0.25 | (0.08 - 0.43) |
| Alcohol Use | Postpartum | 33 | 0.29 | (0.1 - 0.48) |
| Alcohol Use | Postpartum | 34 | 0.32 | (0.12 - 0.52) |
| Alcohol Use | Postpartum | 35 | 0.29 | (0.1 - 0.47) |
| Alcohol Use | Postpartum | 36 | 0.22 | (0.06 - 0.39) |
| Alcohol Use | Postpartum | 37 | 0.29 | (0.1 - 0.48) |
| Alcohol Use | Postpartum | 38 | 0.64 | (0.36 - 0.93) |
| Alcohol Use | Postpartum | 39 | 0.45 | (0.22 - 0.69) |
| Alcohol Use | Postpartum | 40 | 0.49 | (0.24 - 0.73) |
| Alcohol Use | Postpartum | 41 | 0.44 | (0.2 - 0.67) |
| Alcohol Use | Postpartum | 42 | 0.56 | (0.29 - 0.82) |
| Alcohol Use | Postpartum | 43 | 0.39 | (0.17 - 0.61) |
| Alcohol Use | Postpartum | 44 | 0.33 | (0.12 - 0.53) |
| Alcohol Use | Postpartum | 45 | 0.31 | (0.11 - 0.51) |
| Alcohol Use | Postpartum | 46 | 0.43 | (0.19 - 0.66) |
| Alcohol Use | Postpartum | 47 | 0.75 | (0.43 - 1.06) |
| Alcohol Use | Postpartum | 48 | 0.68 | (0.38 - 0.98) |
| Alcohol Use | Postpartum | 49 | 0.37 | (0.15 - 0.59) |
| Alcohol Use | Postpartum | 50 | 0.66 | (0.36 - 0.96) |
| Alcohol Use | Postpartum | 51 | 0.46 | (0.22 - 0.69) |
| Other Substance Use | Preconception | 0 | 2.75 | (2.1 - 3.4) |
| Other Substance Use | Preconception | 1 | 1.74 | (1.23 - 2.26) |
| Other Substance Use | Preconception | 2 | 1.83 | (1.3 - 2.36) |
| Other Substance Use | Preconception | 3 | 1.92 | (1.38 - 2.46) |
| Other Substance Use | Preconception | 4 | 2.03 | (1.48 - 2.58) |
| Other Substance Use | Preconception | 5 | 2.11 | (1.55 - 2.67) |
| Other Substance Use | Preconception | 6 | 2.26 | (1.68 - 2.84) |
| Other Substance Use | Preconception | 7 | 2.08 | (1.52 - 2.63) |
| Other Substance Use | Preconception | 8 | 2.28 | (1.7 - 2.87) |
| Other Substance Use | Preconception | 9 | 1.73 | (1.22 - 2.23) |
| Other Substance Use | Preconception | 10 | 2.46 | (1.85 - 3.06) |
| Other Substance Use | Preconception | 11 | 2.22 | (1.65 - 2.79) |
| Other Substance Use | Preconception | 12 | 2.14 | (1.58 - 2.7) |
| Other Substance Use | Preconception | 13 | 1.82 | (1.3 - 2.33) |
| Other Substance Use | Preconception | 14 | 2.16 | (1.6 - 2.72) |
| Other Substance Use | Preconception | 15 | 1.58 | (1.1 - 2.06) |
| Other Substance Use | Preconception | 16 | 2.29 | (1.72 - 2.87) |
| Other Substance Use | Preconception | 17 | 2.32 | (1.74 - 2.89) |
| Other Substance Use | Preconception | 18 | 1.94 | (1.41 - 2.46) |
| Other Substance Use | Preconception | 19 | 1.97 | (1.44 - 2.5) |
| Other Substance Use | Preconception | 20 | 1.14 | (0.74 - 1.55) |
| Other Substance Use | Preconception | 21 | 1.55 | (1.08 - 2.01) |
| Other Substance Use | Preconception | 22 | 1.58 | (1.11 - 2.05) |
| Other Substance Use | Preconception | 23 | 1.61 | (1.13 - 2.08) |
| Other Substance Use | Preconception | 24 | 2.15 | (1.6 - 2.7) |
| Other Substance Use | Preconception | 25 | 1.92 | (1.41 - 2.44) |
| Other Substance Use | Preconception | 26 | 2.24 | (1.68 - 2.8) |
| Other Substance Use | Preconception | 27 | 1.76 | (1.27 - 2.25) |
| Other Substance Use | Preconception | 28 | 1.58 | (1.11 - 2.04) |
| Other Substance Use | Preconception | 29 | 1.96 | (1.44 - 2.48) |
| Other Substance Use | Preconception | 30 | 2.17 | (1.62 - 2.71) |
| Other Substance Use | Preconception | 31 | 1.81 | (1.31 - 2.3) |
| Other Substance Use | Preconception | 32 | 1.80 | (1.3 - 2.29) |
| Other Substance Use | Preconception | 33 | 2.21 | (1.66 - 2.76) |
| Other Substance Use | Preconception | 34 | 1.54 | (1.08 - 1.99) |
| Other Substance Use | Preconception | 35 | 1.88 | (1.38 - 2.38) |
| Other Substance Use | Preconception | 36 | 2.08 | (1.55 - 2.6) |
| Other Substance Use | Preconception | 37 | 1.52 | (1.07 - 1.97) |
| Other Substance Use | Preconception | 38 | 2.37 | (1.81 - 2.94) |
| Other Substance Use | Preconception | 39 | 1.68 | (1.21 - 2.15) |
| Other Substance Use | Preconception | 40 | 1.34 | (0.92 - 1.76) |
| Other Substance Use | Preconception | 41 | 2.28 | (1.73 - 2.82) |
| Other Substance Use | Preconception | 42 | 1.99 | (1.48 - 2.49) |
| Other Substance Use | Preconception | 43 | 1.65 | (1.19 - 2.11) |
| Other Substance Use | Preconception | 44 | 2.32 | (1.77 - 2.86) |
| Other Substance Use | Preconception | 45 | 1.58 | (1.12 - 2.03) |
| Other Substance Use | Preconception | 46 | 1.86 | (1.37 - 2.35) |
| Other Substance Use | Preconception | 47 | 1.76 | (1.28 - 2.23) |
| Other Substance Use | Preconception | 48 | 1.64 | (1.18 - 2.09) |
| Other Substance Use | Preconception | 49 | 1.60 | (1.15 - 2.04) |
| Other Substance Use | Preconception | 50 | 1.82 | (1.34 - 2.3) |
| Other Substance Use | Preconception | 51 | 1.71 | (1.25 - 2.17) |
| Other Substance Use | Antepartum | 0 | 1.72 | (1.27 - 2.16) |
| Other Substance Use | Antepartum | 1 | 1.44 | (1.04 - 1.85) |
| Other Substance Use | Antepartum | 2 | 1.26 | (0.88 - 1.65) |
| Other Substance Use | Antepartum | 3 | 1.23 | (0.85 - 1.6) |
| Other Substance Use | Antepartum | 4 | 1.05 | (0.7 - 1.4) |
| Other Substance Use | Antepartum | 5 | 0.69 | (0.41 - 0.97) |
| Other Substance Use | Antepartum | 6 | 0.81 | (0.5 - 1.11) |
| Other Substance Use | Antepartum | 7 | 0.66 | (0.38 - 0.93) |
| Other Substance Use | Antepartum | 8 | 0.60 | (0.34 - 0.86) |
| Other Substance Use | Antepartum | 9 | 0.90 | (0.58 - 1.22) |
| Other Substance Use | Antepartum | 10 | 0.75 | (0.45 - 1.04) |
| Other Substance Use | Antepartum | 11 | 0.93 | (0.6 - 1.25) |
| Other Substance Use | Antepartum | 12 | 0.57 | (0.31 - 0.82) |
| Other Substance Use | Antepartum | 13 | 0.72 | (0.43 - 1) |
| Other Substance Use | Antepartum | 14 | 1.07 | (0.72 - 1.42) |
| Other Substance Use | Antepartum | 15 | 0.71 | (0.43 - 1) |
| Other Substance Use | Antepartum | 16 | 0.80 | (0.5 - 1.11) |
| Other Substance Use | Antepartum | 17 | 0.33 | (0.13 - 0.52) |
| Other Substance Use | Antepartum | 18 | 0.92 | (0.6 - 1.25) |
| Other Substance Use | Antepartum | 19 | 0.65 | (0.38 - 0.93) |
| Other Substance Use | Antepartum | 20 | 0.62 | (0.36 - 0.89) |
| Other Substance Use | Antepartum | 21 | 0.65 | (0.38 - 0.92) |
| Other Substance Use | Antepartum | 22 | 0.86 | (0.55 - 1.17) |
| Other Substance Use | Antepartum | 23 | 0.65 | (0.38 - 0.92) |
| Other Substance Use | Antepartum | 24 | 0.74 | (0.45 - 1.03) |
| Other Substance Use | Antepartum | 25 | 0.62 | (0.36 - 0.89) |
| Other Substance Use | Antepartum | 26 | 0.59 | (0.33 - 0.85) |
| Other Substance Use | Antepartum | 27 | 0.56 | (0.31 - 0.82) |
| Other Substance Use | Antepartum | 28 | 0.59 | (0.33 - 0.85) |
| Other Substance Use | Antepartum | 29 | 0.36 | (0.15 - 0.56) |
| Other Substance Use | Antepartum | 30 | 0.47 | (0.24 - 0.71) |
| Other Substance Use | Antepartum | 31 | 0.33 | (0.13 - 0.52) |
| Other Substance Use | Antepartum | 32 | 0.30 | (0.11 - 0.48) |
| Other Substance Use | Antepartum | 33 | 0.63 | (0.36 - 0.9) |
| Other Substance Use | Antepartum | 34 | 0.24 | (0.07 - 0.41) |
| Other Substance Use | Antepartum | 35 | 0.33 | (0.14 - 0.53) |
| Other Substance Use | Antepartum | 36 | 0.55 | (0.3 - 0.81) |
| Other Substance Use | Antepartum | 37 | 0.19 | (0.04 - 0.35) |
| Other Substance Use | Antepartum | 38 | 0.22 | (0.04 - 0.39) |
| Other Substance Use | Antepartum | 39 | 0.10 | (0 - 0.23) |
| Other Substance Use | Antepartum | 40 | 0.10 | (0 - 0.29) |
| Other Substance Use | Antepartum | 41 | 0.00 | (0 - 0) |
| Other Substance Use | Postpartum | 0 | 0.33 | (0.13 - 0.52) |
| Other Substance Use | Postpartum | 1 | 0.35 | (0.15 - 0.55) |
| Other Substance Use | Postpartum | 2 | 0.49 | (0.26 - 0.73) |
| Other Substance Use | Postpartum | 3 | 0.56 | (0.31 - 0.81) |
| Other Substance Use | Postpartum | 4 | 0.45 | (0.22 - 0.67) |
| Other Substance Use | Postpartum | 5 | 0.38 | (0.17 - 0.59) |
| Other Substance Use | Postpartum | 6 | 0.41 | (0.19 - 0.62) |
| Other Substance Use | Postpartum | 7 | 0.43 | (0.21 - 0.65) |
| Other Substance Use | Postpartum | 8 | 0.65 | (0.38 - 0.93) |
| Other Substance Use | Postpartum | 9 | 0.62 | (0.36 - 0.89) |
| Other Substance Use | Postpartum | 10 | 0.47 | (0.24 - 0.7) |
| Other Substance Use | Postpartum | 11 | 0.62 | (0.36 - 0.89) |
| Other Substance Use | Postpartum | 12 | 0.93 | (0.6 - 1.25) |
| Other Substance Use | Postpartum | 13 | 0.77 | (0.47 - 1.07) |
| Other Substance Use | Postpartum | 14 | 0.75 | (0.46 - 1.05) |
| Other Substance Use | Postpartum | 15 | 0.72 | (0.43 - 1.01) |
| Other Substance Use | Postpartum | 16 | 0.78 | (0.48 - 1.08) |
| Other Substance Use | Postpartum | 17 | 0.78 | (0.48 - 1.08) |
| Other Substance Use | Postpartum | 18 | 0.88 | (0.56 - 1.19) |
| Other Substance Use | Postpartum | 19 | 1.03 | (0.68 - 1.38) |
| Other Substance Use | Postpartum | 20 | 0.70 | (0.41 - 0.98) |
| Other Substance Use | Postpartum | 21 | 0.95 | (0.61 - 1.28) |
| Other Substance Use | Postpartum | 22 | 0.70 | (0.42 - 0.99) |
| Other Substance Use | Postpartum | 23 | 0.86 | (0.54 - 1.17) |
| Other Substance Use | Postpartum | 24 | 0.64 | (0.37 - 0.92) |
| Other Substance Use | Postpartum | 25 | 0.80 | (0.49 - 1.11) |
| Other Substance Use | Postpartum | 26 | 1.24 | (0.85 - 1.62) |
| Other Substance Use | Postpartum | 27 | 1.06 | (0.7 - 1.41) |
| Other Substance Use | Postpartum | 28 | 1.12 | (0.75 - 1.49) |
| Other Substance Use | Postpartum | 29 | 0.88 | (0.55 - 1.2) |
| Other Substance Use | Postpartum | 30 | 0.75 | (0.45 - 1.05) |
| Other Substance Use | Postpartum | 31 | 0.91 | (0.58 - 1.24) |
| Other Substance Use | Postpartum | 32 | 0.79 | (0.48 - 1.1) |
| Other Substance Use | Postpartum | 33 | 0.88 | (0.56 - 1.21) |
| Other Substance Use | Postpartum | 34 | 0.77 | (0.46 - 1.07) |
| Other Substance Use | Postpartum | 35 | 0.58 | (0.31 - 0.85) |
| Other Substance Use | Postpartum | 36 | 1.07 | (0.7 - 1.43) |
| Other Substance Use | Postpartum | 37 | 1.04 | (0.68 - 1.4) |
| Other Substance Use | Postpartum | 38 | 1.04 | (0.68 - 1.39) |
| Other Substance Use | Postpartum | 39 | 1.23 | (0.84 - 1.62) |
| Other Substance Use | Postpartum | 40 | 0.94 | (0.6 - 1.28) |
| Other Substance Use | Postpartum | 41 | 0.69 | (0.39 - 0.98) |
| Other Substance Use | Postpartum | 42 | 1.15 | (0.77 - 1.54) |
| Other Substance Use | Postpartum | 43 | 1.02 | (0.66 - 1.38) |
| Other Substance Use | Postpartum | 44 | 0.99 | (0.64 - 1.35) |
| Other Substance Use | Postpartum | 45 | 0.90 | (0.56 - 1.23) |
| Other Substance Use | Postpartum | 46 | 0.97 | (0.62 - 1.33) |
| Other Substance Use | Postpartum | 47 | 1.04 | (0.67 - 1.41) |
| Other Substance Use | Postpartum | 48 | 1.24 | (0.83 - 1.64) |
| Other Substance Use | Postpartum | 49 | 1.02 | (0.65 - 1.38) |
| Other Substance Use | Postpartum | 50 | 0.83 | (0.5 - 1.16) |
| Other Substance Use | Postpartum | 51 | 1.39 | (0.99 - 1.8) |
| Psychosis | Preconception | 0 | 0.28 | (0.07 - 0.49) |
| Psychosis | Preconception | 1 | 0.19 | (0.02 - 0.36) |
| Psychosis | Preconception | 2 | 0.23 | (0.05 - 0.42) |
| Psychosis | Preconception | 3 | 0.42 | (0.17 - 0.67) |
| Psychosis | Preconception | 4 | 0.23 | (0.05 - 0.41) |
| Psychosis | Preconception | 5 | 0.08 | (0 - 0.18) |
| Psychosis | Preconception | 6 | 0.34 | (0.12 - 0.56) |
| Psychosis | Preconception | 7 | 0.19 | (0.02 - 0.36) |
| Psychosis | Preconception | 8 | 0.41 | (0.17 - 0.66) |
| Psychosis | Preconception | 9 | 0.11 | (0 - 0.24) |
| Psychosis | Preconception | 10 | 0.27 | (0.07 - 0.46) |
| Psychosis | Preconception | 11 | 0.12 | (0 - 0.25) |
| Psychosis | Preconception | 12 | 0.38 | (0.14 - 0.61) |
| Psychosis | Preconception | 13 | 0.34 | (0.12 - 0.56) |
| Psychosis | Preconception | 14 | 0.33 | (0.12 - 0.55) |
| Psychosis | Preconception | 15 | 0.19 | (0.02 - 0.35) |
| Psychosis | Preconception | 16 | 0.07 | (0 - 0.18) |
| Psychosis | Preconception | 17 | 0.19 | (0.02 - 0.35) |
| Psychosis | Preconception | 18 | 0.11 | (0 - 0.24) |
| Psychosis | Preconception | 19 | 0.33 | (0.11 - 0.55) |
| Psychosis | Preconception | 20 | 0.37 | (0.14 - 0.59) |
| Psychosis | Preconception | 21 | 0.29 | (0.09 - 0.49) |
| Psychosis | Preconception | 22 | 0.25 | (0.07 - 0.44) |
| Psychosis | Preconception | 23 | 0.18 | (0.02 - 0.34) |
| Psychosis | Preconception | 24 | 0.40 | (0.16 - 0.63) |
| Psychosis | Preconception | 25 | 0.39 | (0.16 - 0.63) |
| Psychosis | Preconception | 26 | 0.43 | (0.19 - 0.67) |
| Psychosis | Preconception | 27 | 0.32 | (0.11 - 0.53) |
| Psychosis | Preconception | 28 | 0.18 | (0.02 - 0.33) |
| Psychosis | Preconception | 29 | 0.32 | (0.11 - 0.52) |
| Psychosis | Preconception | 30 | 0.25 | (0.06 - 0.43) |
| Psychosis | Preconception | 31 | 0.21 | (0.04 - 0.38) |
| Psychosis | Preconception | 32 | 0.24 | (0.06 - 0.42) |
| Psychosis | Preconception | 33 | 0.38 | (0.16 - 0.6) |
| Psychosis | Preconception | 34 | 0.49 | (0.23 - 0.74) |
| Psychosis | Preconception | 35 | 0.17 | (0.02 - 0.32) |
| Psychosis | Preconception | 36 | 0.14 | (0 - 0.27) |
| Psychosis | Preconception | 37 | 0.17 | (0.02 - 0.32) |
| Psychosis | Preconception | 38 | 0.24 | (0.06 - 0.41) |
| Psychosis | Preconception | 39 | 0.10 | (0 - 0.22) |
| Psychosis | Preconception | 40 | 0.27 | (0.08 - 0.45) |
| Psychosis | Preconception | 41 | 0.27 | (0.08 - 0.46) |
| Psychosis | Preconception | 42 | 0.27 | (0.08 - 0.45) |
| Psychosis | Preconception | 43 | 0.30 | (0.1 - 0.5) |
| Psychosis | Preconception | 44 | 0.30 | (0.1 - 0.49) |
| Psychosis | Preconception | 45 | 0.30 | (0.1 - 0.5) |
| Psychosis | Preconception | 46 | 0.23 | (0.06 - 0.39) |
| Psychosis | Preconception | 47 | 0.23 | (0.06 - 0.4) |
| Psychosis | Preconception | 48 | 0.16 | (0.02 - 0.31) |
| Psychosis | Preconception | 49 | 0.23 | (0.06 - 0.4) |
| Psychosis | Preconception | 50 | 0.32 | (0.12 - 0.52) |
| Psychosis | Preconception | 51 | 0.10 | (0 - 0.2) |
| Psychosis | Antepartum | 0 | 0.30 | (0.11 - 0.48) |
| Psychosis | Antepartum | 1 | 0.18 | (0.04 - 0.32) |
| Psychosis | Antepartum | 2 | 0.33 | (0.13 - 0.52) |
| Psychosis | Antepartum | 3 | 0.12 | (0 - 0.24) |
| Psychosis | Antepartum | 4 | 0.18 | (0.04 - 0.32) |
| Psychosis | Antepartum | 5 | 0.15 | (0.02 - 0.28) |
| Psychosis | Antepartum | 6 | 0.09 | (0 - 0.19) |
| Psychosis | Antepartum | 7 | 0.15 | (0.02 - 0.28) |
| Psychosis | Antepartum | 8 | 0.15 | (0.02 - 0.28) |
| Psychosis | Antepartum | 9 | 0.15 | (0.02 - 0.28) |
| Psychosis | Antepartum | 10 | 0.24 | (0.07 - 0.4) |
| Psychosis | Antepartum | 11 | 0.09 | (0 - 0.19) |
| Psychosis | Antepartum | 12 | 0.09 | (0 - 0.19) |
| Psychosis | Antepartum | 13 | 0.06 | (0 - 0.14) |
| Psychosis | Antepartum | 14 | 0.09 | (0 - 0.19) |
| Psychosis | Antepartum | 15 | 0.21 | (0.05 - 0.36) |
| Psychosis | Antepartum | 16 | 0.15 | (0.02 - 0.28) |
| Psychosis | Antepartum | 17 | 0.12 | (0 - 0.23) |
| Psychosis | Antepartum | 18 | 0.26 | (0.09 - 0.44) |
| Psychosis | Antepartum | 19 | 0.18 | (0.04 - 0.32) |
| Psychosis | Antepartum | 20 | 0.18 | (0.04 - 0.32) |
| Psychosis | Antepartum | 21 | 0.12 | (0 - 0.23) |
| Psychosis | Antepartum | 22 | 0.12 | (0 - 0.23) |
| Psychosis | Antepartum | 23 | 0.18 | (0.04 - 0.32) |
| Psychosis | Antepartum | 24 | 0.29 | (0.11 - 0.47) |
| Psychosis | Antepartum | 25 | 0.12 | (0 - 0.23) |
| Psychosis | Antepartum | 26 | 0.15 | (0.02 - 0.27) |
| Psychosis | Antepartum | 27 | 0.15 | (0.02 - 0.27) |
| Psychosis | Antepartum | 28 | 0.18 | (0.04 - 0.32) |
| Psychosis | Antepartum | 29 | 0.21 | (0.05 - 0.36) |
| Psychosis | Antepartum | 30 | 0.06 | (0 - 0.14) |
| Psychosis | Antepartum | 31 | 0.23 | (0.07 - 0.4) |
| Psychosis | Antepartum | 32 | 0.15 | (0.02 - 0.28) |
| Psychosis | Antepartum | 33 | 0.15 | (0.02 - 0.28) |
| Psychosis | Antepartum | 34 | 0.12 | (0 - 0.24) |
| Psychosis | Antepartum | 35 | 0.06 | (0 - 0.14) |
| Psychosis | Antepartum | 36 | 0.09 | (0 - 0.19) |
| Psychosis | Antepartum | 37 | 0.06 | (0 - 0.15) |
| Psychosis | Antepartum | 38 | 0.22 | (0.04 - 0.39) |
| Psychosis | Antepartum | 39 | 0.14 | (0 - 0.31) |
| Psychosis | Antepartum | 40 | 0.09 | (0 - 0.26) |
| Psychosis | Antepartum | 41 | 0.26 | (0 - 0.77) |
| Psychosis | Postpartum | 0 | 2.13 | (1.64 - 2.61) |
| Psychosis | Postpartum | 1 | 2.46 | (1.93 - 2.98) |
| Psychosis | Postpartum | 2 | 1.41 | (1.01 - 1.8) |
| Psychosis | Postpartum | 3 | 0.90 | (0.58 - 1.21) |
| Psychosis | Postpartum | 4 | 0.93 | (0.61 - 1.25) |
| Psychosis | Postpartum | 5 | 0.79 | (0.49 - 1.09) |
| Psychosis | Postpartum | 6 | 0.96 | (0.63 - 1.28) |
| Psychosis | Postpartum | 7 | 0.88 | (0.57 - 1.2) |
| Psychosis | Postpartum | 8 | 0.78 | (0.49 - 1.08) |
| Psychosis | Postpartum | 9 | 0.78 | (0.49 - 1.08) |
| Psychosis | Postpartum | 10 | 0.67 | (0.39 - 0.94) |
| Psychosis | Postpartum | 11 | 0.70 | (0.42 - 0.99) |
| Psychosis | Postpartum | 12 | 0.67 | (0.4 - 0.95) |
| Psychosis | Postpartum | 13 | 0.68 | (0.4 - 0.96) |
| Psychosis | Postpartum | 14 | 0.56 | (0.31 - 0.81) |
| Psychosis | Postpartum | 15 | 0.44 | (0.22 - 0.66) |
| Psychosis | Postpartum | 16 | 0.68 | (0.4 - 0.96) |
| Psychosis | Postpartum | 17 | 0.44 | (0.22 - 0.67) |
| Psychosis | Postpartum | 18 | 0.60 | (0.34 - 0.86) |
| Psychosis | Postpartum | 19 | 0.84 | (0.53 - 1.15) |
| Psychosis | Postpartum | 20 | 0.30 | (0.11 - 0.49) |
| Psychosis | Postpartum | 21 | 0.42 | (0.2 - 0.64) |
| Psychosis | Postpartum | 22 | 0.45 | (0.22 - 0.68) |
| Psychosis | Postpartum | 23 | 0.36 | (0.16 - 0.57) |
| Psychosis | Postpartum | 24 | 0.37 | (0.16 - 0.57) |
| Psychosis | Postpartum | 25 | 0.40 | (0.18 - 0.61) |
| Psychosis | Postpartum | 26 | 0.31 | (0.12 - 0.49) |
| Psychosis | Postpartum | 27 | 0.46 | (0.23 - 0.69) |
| Psychosis | Postpartum | 28 | 0.43 | (0.21 - 0.66) |
| Psychosis | Postpartum | 29 | 0.50 | (0.25 - 0.74) |
| Psychosis | Postpartum | 30 | 0.53 | (0.28 - 0.78) |
| Psychosis | Postpartum | 31 | 0.31 | (0.12 - 0.5) |
| Psychosis | Postpartum | 32 | 0.22 | (0.06 - 0.38) |
| Psychosis | Postpartum | 33 | 0.38 | (0.17 - 0.6) |
| Psychosis | Postpartum | 34 | 0.41 | (0.19 - 0.63) |
| Psychosis | Postpartum | 35 | 0.12 | (0 - 0.25) |
| Psychosis | Postpartum | 36 | 0.35 | (0.14 - 0.55) |
| Psychosis | Postpartum | 37 | 0.19 | (0.04 - 0.34) |
| Psychosis | Postpartum | 38 | 0.32 | (0.12 - 0.52) |
| Psychosis | Postpartum | 39 | 0.42 | (0.19 - 0.64) |
| Psychosis | Postpartum | 40 | 0.38 | (0.17 - 0.6) |
| Psychosis | Postpartum | 41 | 0.30 | (0.1 - 0.49) |
| Psychosis | Postpartum | 42 | 0.19 | (0.04 - 0.35) |
| Psychosis | Postpartum | 43 | 0.37 | (0.15 - 0.58) |
| Psychosis | Postpartum | 44 | 0.26 | (0.08 - 0.44) |
| Psychosis | Postpartum | 45 | 0.10 | (0 - 0.2) |
| Psychosis | Postpartum | 46 | 0.36 | (0.15 - 0.57) |
| Psychosis | Postpartum | 47 | 0.36 | (0.15 - 0.58) |
| Psychosis | Postpartum | 48 | 0.20 | (0.04 - 0.36) |
| Psychosis | Postpartum | 49 | 0.27 | (0.08 - 0.46) |
| Psychosis | Postpartum | 50 | 0.20 | (0.04 - 0.36) |
| Psychosis | Postpartum | 51 | 0.15 | (0.02 - 0.28) |
| Bipolar | Preconception | 0 | 1.08 | (0.67 - 1.49) |
| Bipolar | Preconception | 1 | 0.87 | (0.51 - 1.23) |
| Bipolar | Preconception | 2 | 0.79 | (0.44 - 1.14) |
| Bipolar | Preconception | 3 | 0.75 | (0.41 - 1.09) |
| Bipolar | Preconception | 4 | 0.90 | (0.53 - 1.27) |
| Bipolar | Preconception | 5 | 1.02 | (0.63 - 1.41) |
| Bipolar | Preconception | 6 | 1.17 | (0.75 - 1.59) |
| Bipolar | Preconception | 7 | 0.92 | (0.55 - 1.29) |
| Bipolar | Preconception | 8 | 1.35 | (0.9 - 1.8) |
| Bipolar | Preconception | 9 | 0.62 | (0.31 - 0.92) |
| Bipolar | Preconception | 10 | 0.54 | (0.26 - 0.82) |
| Bipolar | Preconception | 11 | 0.57 | (0.28 - 0.86) |
| Bipolar | Preconception | 12 | 0.46 | (0.2 - 0.71) |
| Bipolar | Preconception | 13 | 0.76 | (0.43 - 1.09) |
| Bipolar | Preconception | 14 | 0.83 | (0.48 - 1.17) |
| Bipolar | Preconception | 15 | 0.86 | (0.51 - 1.21) |
| Bipolar | Preconception | 16 | 1.05 | (0.66 - 1.43) |
| Bipolar | Preconception | 17 | 1.04 | (0.65 - 1.42) |
| Bipolar | Preconception | 18 | 1.15 | (0.75 - 1.55) |
| Bipolar | Preconception | 19 | 0.92 | (0.56 - 1.28) |
| Bipolar | Preconception | 20 | 0.85 | (0.5 - 1.19) |
| Bipolar | Preconception | 21 | 0.91 | (0.56 - 1.27) |
| Bipolar | Preconception | 22 | 0.55 | (0.27 - 0.82) |
| Bipolar | Preconception | 23 | 0.54 | (0.27 - 0.82) |
| Bipolar | Preconception | 24 | 0.72 | (0.41 - 1.04) |
| Bipolar | Preconception | 25 | 0.93 | (0.58 - 1.29) |
| Bipolar | Preconception | 26 | 0.79 | (0.46 - 1.12) |
| Bipolar | Preconception | 27 | 0.86 | (0.51 - 1.2) |
| Bipolar | Preconception | 28 | 0.71 | (0.4 - 1.02) |
| Bipolar | Preconception | 29 | 0.92 | (0.56 - 1.27) |
| Bipolar | Preconception | 30 | 0.67 | (0.37 - 0.97) |
| Bipolar | Preconception | 31 | 0.91 | (0.56 - 1.26) |
| Bipolar | Preconception | 32 | 0.94 | (0.59 - 1.29) |
| Bipolar | Preconception | 33 | 0.83 | (0.5 - 1.16) |
| Bipolar | Preconception | 34 | 0.76 | (0.44 - 1.08) |
| Bipolar | Preconception | 35 | 0.79 | (0.47 - 1.12) |
| Bipolar | Preconception | 36 | 0.72 | (0.41 - 1.02) |
| Bipolar | Preconception | 37 | 0.58 | (0.3 - 0.86) |
| Bipolar | Preconception | 38 | 0.65 | (0.36 - 0.94) |
| Bipolar | Preconception | 39 | 1.05 | (0.68 - 1.41) |
| Bipolar | Preconception | 40 | 0.57 | (0.3 - 0.84) |
| Bipolar | Preconception | 41 | 0.64 | (0.35 - 0.92) |
| Bipolar | Preconception | 42 | 0.73 | (0.42 - 1.03) |
| Bipolar | Preconception | 43 | 0.60 | (0.32 - 0.87) |
| Bipolar | Preconception | 44 | 0.72 | (0.42 - 1.03) |
| Bipolar | Preconception | 45 | 0.46 | (0.22 - 0.7) |
| Bipolar | Preconception | 46 | 0.92 | (0.58 - 1.26) |
| Bipolar | Preconception | 47 | 0.78 | (0.47 - 1.09) |
| Bipolar | Preconception | 48 | 0.68 | (0.39 - 0.97) |
| Bipolar | Preconception | 49 | 1.00 | (0.65 - 1.36) |
| Bipolar | Preconception | 50 | 0.74 | (0.44 - 1.05) |
| Bipolar | Preconception | 51 | 0.67 | (0.38 - 0.96) |
| Bipolar | Antepartum | 0 | 0.98 | (0.65 - 1.32) |
| Bipolar | Antepartum | 1 | 0.68 | (0.4 - 0.96) |
| Bipolar | Antepartum | 2 | 0.65 | (0.38 - 0.93) |
| Bipolar | Antepartum | 3 | 0.59 | (0.33 - 0.85) |
| Bipolar | Antepartum | 4 | 0.74 | (0.45 - 1.03) |
| Bipolar | Antepartum | 5 | 0.92 | (0.6 - 1.24) |
| Bipolar | Antepartum | 6 | 0.59 | (0.33 - 0.85) |
| Bipolar | Antepartum | 7 | 0.71 | (0.43 - 0.99) |
| Bipolar | Antepartum | 8 | 0.50 | (0.26 - 0.74) |
| Bipolar | Antepartum | 9 | 0.56 | (0.31 - 0.81) |
| Bipolar | Antepartum | 10 | 0.21 | (0.05 - 0.36) |
| Bipolar | Antepartum | 11 | 0.38 | (0.18 - 0.59) |
| Bipolar | Antepartum | 12 | 0.47 | (0.24 - 0.7) |
| Bipolar | Antepartum | 13 | 0.71 | (0.42 - 0.99) |
| Bipolar | Antepartum | 14 | 0.38 | (0.18 - 0.59) |
| Bipolar | Antepartum | 15 | 0.50 | (0.26 - 0.74) |
| Bipolar | Antepartum | 16 | 0.32 | (0.13 - 0.52) |
| Bipolar | Antepartum | 17 | 0.32 | (0.13 - 0.51) |
| Bipolar | Antepartum | 18 | 0.38 | (0.17 - 0.59) |
| Bipolar | Antepartum | 19 | 0.41 | (0.2 - 0.63) |
| Bipolar | Antepartum | 20 | 0.50 | (0.26 - 0.74) |
| Bipolar | Antepartum | 21 | 0.38 | (0.17 - 0.59) |
| Bipolar | Antepartum | 22 | 0.35 | (0.15 - 0.55) |
| Bipolar | Antepartum | 23 | 0.41 | (0.2 - 0.63) |
| Bipolar | Antepartum | 24 | 0.44 | (0.22 - 0.66) |
| Bipolar | Antepartum | 25 | 0.41 | (0.2 - 0.63) |
| Bipolar | Antepartum | 26 | 0.35 | (0.15 - 0.55) |
| Bipolar | Antepartum | 27 | 0.29 | (0.11 - 0.48) |
| Bipolar | Antepartum | 28 | 0.53 | (0.28 - 0.77) |
| Bipolar | Antepartum | 29 | 0.53 | (0.28 - 0.77) |
| Bipolar | Antepartum | 30 | 0.50 | (0.26 - 0.74) |
| Bipolar | Antepartum | 31 | 0.38 | (0.17 - 0.59) |
| Bipolar | Antepartum | 32 | 0.32 | (0.13 - 0.52) |
| Bipolar | Antepartum | 33 | 0.41 | (0.2 - 0.63) |
| Bipolar | Antepartum | 34 | 0.33 | (0.13 - 0.52) |
| Bipolar | Antepartum | 35 | 0.24 | (0.07 - 0.41) |
| Bipolar | Antepartum | 36 | 0.24 | (0.08 - 0.41) |
| Bipolar | Antepartum | 37 | 0.32 | (0.12 - 0.52) |
| Bipolar | Antepartum | 38 | 0.18 | (0.02 - 0.34) |
| Bipolar | Antepartum | 39 | 0.10 | (0 - 0.23) |
| Bipolar | Antepartum | 40 | 0.00 | (0 - 0) |
| Bipolar | Antepartum | 41 | 0.00 | (0 - 0) |
| Bipolar | Postpartum | 0 | 0.55 | (0.3 - 0.8) |
| Bipolar | Postpartum | 1 | 0.97 | (0.64 - 1.3) |
| Bipolar | Postpartum | 2 | 0.74 | (0.45 - 1.03) |
| Bipolar | Postpartum | 3 | 0.20 | (0.05 - 0.35) |
| Bipolar | Postpartum | 4 | 0.20 | (0.05 - 0.35) |
| Bipolar | Postpartum | 5 | 0.53 | (0.29 - 0.78) |
| Bipolar | Postpartum | 6 | 0.38 | (0.17 - 0.59) |
| Bipolar | Postpartum | 7 | 0.49 | (0.26 - 0.73) |
| Bipolar | Postpartum | 8 | 0.49 | (0.26 - 0.72) |
| Bipolar | Postpartum | 9 | 0.56 | (0.31 - 0.81) |
| Bipolar | Postpartum | 10 | 0.59 | (0.33 - 0.85) |
| Bipolar | Postpartum | 11 | 0.50 | (0.26 - 0.74) |
| Bipolar | Postpartum | 12 | 0.47 | (0.24 - 0.71) |
| Bipolar | Postpartum | 13 | 0.53 | (0.29 - 0.78) |
| Bipolar | Postpartum | 14 | 0.65 | (0.38 - 0.92) |
| Bipolar | Postpartum | 15 | 0.45 | (0.22 - 0.67) |
| Bipolar | Postpartum | 16 | 0.54 | (0.29 - 0.79) |
| Bipolar | Postpartum | 17 | 0.54 | (0.29 - 0.79) |
| Bipolar | Postpartum | 18 | 0.45 | (0.22 - 0.67) |
| Bipolar | Postpartum | 19 | 0.39 | (0.18 - 0.6) |
| Bipolar | Postpartum | 20 | 0.48 | (0.25 - 0.72) |
| Bipolar | Postpartum | 21 | 0.45 | (0.22 - 0.68) |
| Bipolar | Postpartum | 22 | 0.79 | (0.48 - 1.09) |
| Bipolar | Postpartum | 23 | 1.06 | (0.71 - 1.42) |
| Bipolar | Postpartum | 24 | 0.76 | (0.46 - 1.06) |
| Bipolar | Postpartum | 25 | 0.37 | (0.16 - 0.57) |
| Bipolar | Postpartum | 26 | 0.64 | (0.37 - 0.92) |
| Bipolar | Postpartum | 27 | 0.68 | (0.39 - 0.96) |
| Bipolar | Postpartum | 28 | 0.71 | (0.42 - 1) |
| Bipolar | Postpartum | 29 | 0.68 | (0.4 - 0.97) |
| Bipolar | Postpartum | 30 | 0.62 | (0.35 - 0.9) |
| Bipolar | Postpartum | 31 | 0.69 | (0.4 - 0.97) |
| Bipolar | Postpartum | 32 | 0.66 | (0.38 - 0.94) |
| Bipolar | Postpartum | 33 | 0.44 | (0.21 - 0.67) |
| Bipolar | Postpartum | 34 | 0.69 | (0.4 - 0.98) |
| Bipolar | Postpartum | 35 | 0.73 | (0.43 - 1.03) |
| Bipolar | Postpartum | 36 | 0.76 | (0.46 - 1.07) |
| Bipolar | Postpartum | 37 | 0.48 | (0.24 - 0.73) |
| Bipolar | Postpartum | 38 | 0.48 | (0.24 - 0.72) |
| Bipolar | Postpartum | 39 | 0.57 | (0.31 - 0.84) |
| Bipolar | Postpartum | 40 | 0.52 | (0.26 - 0.77) |
| Bipolar | Postpartum | 41 | 0.55 | (0.29 - 0.81) |
| Bipolar | Postpartum | 42 | 0.65 | (0.36 - 0.93) |
| Bipolar | Postpartum | 43 | 0.77 | (0.46 - 1.08) |
| Bipolar | Postpartum | 44 | 0.79 | (0.47 - 1.11) |
| Bipolar | Postpartum | 45 | 0.79 | (0.47 - 1.11) |
| Bipolar | Postpartum | 46 | 0.89 | (0.55 - 1.22) |
| Bipolar | Postpartum | 47 | 0.73 | (0.43 - 1.04) |
| Bipolar | Postpartum | 48 | 0.50 | (0.25 - 0.76) |
| Bipolar | Postpartum | 49 | 0.77 | (0.46 - 1.09) |
| Bipolar | Postpartum | 50 | 0.84 | (0.51 - 1.17) |
| Bipolar | Postpartum | 51 | 0.84 | (0.53 - 1.15) |
| Stress-Related | Preconception | 0 | 14.17 | (12.65 - 15.69) |
| Stress-Related | Preconception | 1 | 14.03 | (12.53 - 15.54) |
| Stress-Related | Preconception | 2 | 11.83 | (10.45 - 13.21) |
| Stress-Related | Preconception | 3 | 13.02 | (11.58 - 14.46) |
| Stress-Related | Preconception | 4 | 12.19 | (10.79 - 13.58) |
| Stress-Related | Preconception | 5 | 11.46 | (10.11 - 12.81) |
| Stress-Related | Preconception | 6 | 13.93 | (12.44 - 15.41) |
| Stress-Related | Preconception | 7 | 12.86 | (11.44 - 14.29) |
| Stress-Related | Preconception | 8 | 12.46 | (11.06 - 13.86) |
| Stress-Related | Preconception | 9 | 12.81 | (11.4 - 14.23) |
| Stress-Related | Preconception | 10 | 12.42 | (11.02 - 13.81) |
| Stress-Related | Preconception | 11 | 12.97 | (11.55 - 14.39) |
| Stress-Related | Preconception | 12 | 12.79 | (11.39 - 14.2) |
| Stress-Related | Preconception | 13 | 12.18 | (10.81 - 13.55) |
| Stress-Related | Preconception | 14 | 12.17 | (10.8 - 13.54) |
| Stress-Related | Preconception | 15 | 14.87 | (13.36 - 16.37) |
| Stress-Related | Preconception | 16 | 11.69 | (10.35 - 13.02) |
| Stress-Related | Preconception | 17 | 13.84 | (12.4 - 15.29) |
| Stress-Related | Preconception | 18 | 14.93 | (13.43 - 16.43) |
| Stress-Related | Preconception | 19 | 11.89 | (10.55 - 13.22) |
| Stress-Related | Preconception | 20 | 13.16 | (11.76 - 14.57) |
| Stress-Related | Preconception | 21 | 12.57 | (11.2 - 13.94) |
| Stress-Related | Preconception | 22 | 12.94 | (11.55 - 14.32) |
| Stress-Related | Preconception | 23 | 12.81 | (11.44 - 14.19) |
| Stress-Related | Preconception | 24 | 13.10 | (11.71 - 14.49) |
| Stress-Related | Preconception | 25 | 12.74 | (11.37 - 14.11) |
| Stress-Related | Preconception | 26 | 13.29 | (11.9 - 14.68) |
| Stress-Related | Preconception | 27 | 13.16 | (11.78 - 14.54) |
| Stress-Related | Preconception | 28 | 13.86 | (12.44 - 15.27) |
| Stress-Related | Preconception | 29 | 12.83 | (11.47 - 14.19) |
| Stress-Related | Preconception | 30 | 14.24 | (12.81 - 15.67) |
| Stress-Related | Preconception | 31 | 14.10 | (12.68 - 15.52) |
| Stress-Related | Preconception | 32 | 13.04 | (11.67 - 14.4) |
| Stress-Related | Preconception | 33 | 11.94 | (10.64 - 13.24) |
| Stress-Related | Preconception | 34 | 12.34 | (11.02 - 13.66) |
| Stress-Related | Preconception | 35 | 14.52 | (13.09 - 15.95) |
| Stress-Related | Preconception | 36 | 14.14 | (12.73 - 15.55) |
| Stress-Related | Preconception | 37 | 12.73 | (11.4 - 14.07) |
| Stress-Related | Preconception | 38 | 14.34 | (12.93 - 15.75) |
| Stress-Related | Preconception | 39 | 14.17 | (12.77 - 15.57) |
| Stress-Related | Preconception | 40 | 14.31 | (12.9 - 15.71) |
| Stress-Related | Preconception | 41 | 13.76 | (12.39 - 15.14) |
| Stress-Related | Preconception | 42 | 13.22 | (11.87 - 14.56) |
| Stress-Related | Preconception | 43 | 14.99 | (13.56 - 16.42) |
| Stress-Related | Preconception | 44 | 14.29 | (12.9 - 15.68) |
| Stress-Related | Preconception | 45 | 13.95 | (12.58 - 15.32) |
| Stress-Related | Preconception | 46 | 13.89 | (12.53 - 15.26) |
| Stress-Related | Preconception | 47 | 12.81 | (11.5 - 14.12) |
| Stress-Related | Preconception | 48 | 12.33 | (11.05 - 13.61) |
| Stress-Related | Preconception | 49 | 14.97 | (13.56 - 16.38) |
| Stress-Related | Preconception | 50 | 12.37 | (11.09 - 13.64) |
| Stress-Related | Preconception | 51 | 13.96 | (12.61 - 15.31) |
| Stress-Related | Antepartum | 0 | 15.95 | (14.55 - 17.34) |
| Stress-Related | Antepartum | 1 | 12.28 | (11.06 - 13.51) |
| Stress-Related | Antepartum | 2 | 11.42 | (10.24 - 12.6) |
| Stress-Related | Antepartum | 3 | 11.96 | (10.75 - 13.17) |
| Stress-Related | Antepartum | 4 | 11.06 | (9.9 - 12.23) |
| Stress-Related | Antepartum | 5 | 10.42 | (9.3 - 11.55) |
| Stress-Related | Antepartum | 6 | 10.01 | (8.9 - 11.11) |
| Stress-Related | Antepartum | 7 | 9.94 | (8.84 - 11.04) |
| Stress-Related | Antepartum | 8 | 10.16 | (9.05 - 11.28) |
| Stress-Related | Antepartum | 9 | 8.74 | (7.71 - 9.77) |
| Stress-Related | Antepartum | 10 | 8.70 | (7.68 - 9.73) |
| Stress-Related | Antepartum | 11 | 9.30 | (8.24 - 10.37) |
| Stress-Related | Antepartum | 12 | 8.19 | (7.2 - 9.19) |
| Stress-Related | Antepartum | 13 | 8.73 | (7.7 - 9.76) |
| Stress-Related | Antepartum | 14 | 9.01 | (7.97 - 10.06) |
| Stress-Related | Antepartum | 15 | 9.01 | (7.96 - 10.05) |
| Stress-Related | Antepartum | 16 | 9.19 | (8.14 - 10.25) |
| Stress-Related | Antepartum | 17 | 9.10 | (8.05 - 10.15) |
| Stress-Related | Antepartum | 18 | 9.09 | (8.04 - 10.14) |
| Stress-Related | Antepartum | 19 | 8.39 | (7.39 - 9.4) |
| Stress-Related | Antepartum | 20 | 9.97 | (8.87 - 11.07) |
| Stress-Related | Antepartum | 21 | 9.30 | (8.24 - 10.37) |
| Stress-Related | Antepartum | 22 | 9.24 | (8.18 - 10.3) |
| Stress-Related | Antepartum | 23 | 9.75 | (8.66 - 10.83) |
| Stress-Related | Antepartum | 24 | 10.38 | (9.26 - 11.5) |
| Stress-Related | Antepartum | 25 | 9.40 | (8.34 - 10.47) |
| Stress-Related | Antepartum | 26 | 9.59 | (8.52 - 10.67) |
| Stress-Related | Antepartum | 27 | 8.59 | (7.57 - 9.61) |
| Stress-Related | Antepartum | 28 | 9.57 | (8.49 - 10.65) |
| Stress-Related | Antepartum | 29 | 9.33 | (8.26 - 10.39) |
| Stress-Related | Antepartum | 30 | 8.26 | (7.26 - 9.26) |
| Stress-Related | Antepartum | 31 | 7.38 | (6.44 - 8.33) |
| Stress-Related | Antepartum | 32 | 7.50 | (6.54 - 8.45) |
| Stress-Related | Antepartum | 33 | 6.53 | (5.64 - 7.43) |
| Stress-Related | Antepartum | 34 | 5.32 | (4.51 - 6.13) |
| Stress-Related | Antepartum | 35 | 5.33 | (4.52 - 6.14) |
| Stress-Related | Antepartum | 36 | 3.81 | (3.12 - 4.51) |
| Stress-Related | Antepartum | 37 | 3.26 | (2.6 - 3.91) |
| Stress-Related | Antepartum | 38 | 1.13 | (0.72 - 1.54) |
| Stress-Related | Antepartum | 39 | 1.52 | (0.96 - 2.07) |
| Stress-Related | Antepartum | 40 | 1.06 | (0.43 - 1.69) |
| Stress-Related | Antepartum | 41 | 1.67 | (0.33 - 3.01) |
| Stress-Related | Postpartum | 0 | 5.44 | (4.63 - 6.24) |
| Stress-Related | Postpartum | 1 | 7.44 | (6.49 - 8.38) |
| Stress-Related | Postpartum | 2 | 7.46 | (6.52 - 8.41) |
| Stress-Related | Postpartum | 3 | 8.33 | (7.33 - 9.33) |
| Stress-Related | Postpartum | 4 | 9.84 | (8.75 - 10.92) |
| Stress-Related | Postpartum | 5 | 8.59 | (7.57 - 9.6) |
| Stress-Related | Postpartum | 6 | 9.61 | (8.53 - 10.69) |
| Stress-Related | Postpartum | 7 | 9.88 | (8.78 - 10.97) |
| Stress-Related | Postpartum | 8 | 10.32 | (9.2 - 11.43) |
| Stress-Related | Postpartum | 9 | 9.62 | (8.54 - 10.7) |
| Stress-Related | Postpartum | 10 | 8.77 | (7.74 - 9.8) |
| Stress-Related | Postpartum | 11 | 9.04 | (7.99 - 10.08) |
| Stress-Related | Postpartum | 12 | 8.57 | (7.55 - 9.6) |
| Stress-Related | Postpartum | 13 | 9.35 | (8.28 - 10.41) |
| Stress-Related | Postpartum | 14 | 8.53 | (7.5 - 9.55) |
| Stress-Related | Postpartum | 15 | 7.78 | (6.8 - 8.76) |
| Stress-Related | Postpartum | 16 | 7.47 | (6.51 - 8.43) |
| Stress-Related | Postpartum | 17 | 7.83 | (6.85 - 8.82) |
| Stress-Related | Postpartum | 18 | 9.02 | (7.96 - 10.08) |
| Stress-Related | Postpartum | 19 | 8.96 | (7.91 - 10.02) |
| Stress-Related | Postpartum | 20 | 7.14 | (6.19 - 8.08) |
| Stress-Related | Postpartum | 21 | 7.28 | (6.33 - 8.24) |
| Stress-Related | Postpartum | 22 | 8.29 | (7.27 - 9.31) |
| Stress-Related | Postpartum | 23 | 7.79 | (6.8 - 8.77) |
| Stress-Related | Postpartum | 24 | 7.62 | (6.64 - 8.6) |
| Stress-Related | Postpartum | 25 | 6.88 | (5.95 - 7.82) |
| Stress-Related | Postpartum | 26 | 8.06 | (7.05 - 9.07) |
| Stress-Related | Postpartum | 27 | 8.32 | (7.29 - 9.35) |
| Stress-Related | Postpartum | 28 | 6.89 | (5.95 - 7.83) |
| Stress-Related | Postpartum | 29 | 7.34 | (6.37 - 8.31) |
| Stress-Related | Postpartum | 30 | 7.54 | (6.56 - 8.53) |
| Stress-Related | Postpartum | 31 | 7.49 | (6.51 - 8.47) |
| Stress-Related | Postpartum | 32 | 7.91 | (6.9 - 8.93) |
| Stress-Related | Postpartum | 33 | 7.82 | (6.81 - 8.83) |
| Stress-Related | Postpartum | 34 | 8.04 | (7.01 - 9.06) |
| Stress-Related | Postpartum | 35 | 8.42 | (7.37 - 9.47) |
| Stress-Related | Postpartum | 36 | 7.78 | (6.77 - 8.79) |
| Stress-Related | Postpartum | 37 | 8.67 | (7.6 - 9.74) |
| Stress-Related | Postpartum | 38 | 8.50 | (7.43 - 9.56) |
| Stress-Related | Postpartum | 39 | 8.69 | (7.62 - 9.77) |
| Stress-Related | Postpartum | 40 | 7.52 | (6.52 - 8.52) |
| Stress-Related | Postpartum | 41 | 7.88 | (6.85 - 8.91) |
| Stress-Related | Postpartum | 42 | 7.78 | (6.75 - 8.8) |
| Stress-Related | Postpartum | 43 | 9.28 | (8.16 - 10.4) |
| Stress-Related | Postpartum | 44 | 8.20 | (7.14 - 9.26) |
| Stress-Related | Postpartum | 45 | 8.34 | (7.27 - 9.41) |
| Stress-Related | Postpartum | 46 | 9.27 | (8.14 - 10.4) |
| Stress-Related | Postpartum | 47 | 9.07 | (7.95 - 10.19) |
| Stress-Related | Postpartum | 48 | 9.90 | (8.73 - 11.08) |
| Stress-Related | Postpartum | 49 | 9.93 | (8.75 - 11.11) |
| Stress-Related | Postpartum | 50 | 9.59 | (8.43 - 10.75) |
| Stress-Related | Postpartum | 51 | 9.11 | (8.05 - 10.17) |

SIR = incidence rate, per 1,000 person-years; CI = confidence intervals

**Supplementary Table 4.** Incidence rate ratio and 95% confidence interval of psychiatric disorders during and after pregnancy compared with before pregnancy.

| **Psychiatric disorder** | **Phase** | **Week** | **Model 1. IRR 95%CI** | **Model 2. IRR 95%CI** | **Model 3. IRR 95%CI** |
| --- | --- | --- | --- | --- | --- |
| Any | During Pregnancy | 0 | 0.82(0.78 - 0.86) | 0.82(0.78 - 0.86) | 0.86(0.82 - 0.91) |
| Any | During Pregnancy | 5 | 0.6(0.57 - 0.63) | 0.6(0.57 - 0.63) | 0.62(0.58 - 0.65) |
| Any | During Pregnancy | 10 | 0.6(0.57 - 0.64) | 0.61(0.57 - 0.64) | 0.62(0.58 - 0.65) |
| Any | During Pregnancy | 15 | 0.61(0.57 - 0.64) | 0.61(0.58 - 0.64) | 0.62(0.58 - 0.65) |
| Any | During Pregnancy | 20 | 0.62(0.59 - 0.66) | 0.62(0.59 - 0.66) | 0.64(0.6 - 0.67) |
| Any | During Pregnancy | 25 | 0.61(0.58 - 0.64) | 0.61(0.58 - 0.65) | 0.62(0.59 - 0.66) |
| Any | During Pregnancy | 30 | 0.54(0.51 - 0.57) | 0.54(0.51 - 0.57) | 0.55(0.52 - 0.58) |
| Any | During Pregnancy | 35 | 0.26(0.24 - 0.28) | 0.26(0.24 - 0.29) | 0.27(0.25 - 0.29) |
| Any | After Pregnancy | 0 | 0.67(0.63 - 0.7) | 0.67(0.64 - 0.71) | 0.71(0.67 - 0.75) |
| Any | After Pregnancy | 5 | 0.84(0.8 - 0.88) | 0.84(0.8 - 0.89) | 0.87(0.82 - 0.91) |
| Any | After Pregnancy | 10 | 0.89(0.85 - 0.94) | 0.9(0.85 - 0.95) | 0.92(0.87 - 0.97) |
| Any | After Pregnancy | 15 | 0.79(0.75 - 0.83) | 0.8(0.76 - 0.85) | 0.82(0.77 - 0.86) |
| Any | After Pregnancy | 20 | 0.73(0.7 - 0.77) | 0.74(0.7 - 0.78) | 0.76(0.72 - 0.8) |
| Any | After Pregnancy | 25 | 0.76(0.73 - 0.81) | 0.77(0.73 - 0.81) | 0.78(0.74 - 0.82) |
| Any | After Pregnancy | 30 | 0.75(0.71 - 0.8) | 0.76(0.72 - 0.8) | 0.77(0.73 - 0.81) |
| Any | After Pregnancy | 35 | 0.74(0.7 - 0.78) | 0.74(0.7 - 0.78) | 0.75(0.71 - 0.79) |
| Any | After Pregnancy | 40 | 0.78(0.74 - 0.82) | 0.78(0.74 - 0.83) | 0.79(0.75 - 0.84) |
| Any | After Pregnancy | 45 | 0.83(0.79 - 0.88) | 0.83(0.79 - 0.88) | 0.84(0.8 - 0.88) |
| Any | After Pregnancy | 50 | 0.85(0.79 - 0.93) | 0.86(0.79 - 0.93) | 0.87(0.8 - 0.94) |
| Depression | During Pregnancy | 0 | 0.75(0.7 - 0.81) | 0.76(0.7 - 0.81) | 0.79(0.73 - 0.85) |
| Depression | During Pregnancy | 5 | 0.56(0.52 - 0.61) | 0.56(0.52 - 0.61) | 0.58(0.54 - 0.63) |
| Depression | During Pregnancy | 10 | 0.68(0.62 - 0.73) | 0.68(0.63 - 0.73) | 0.69(0.64 - 0.75) |
| Depression | During Pregnancy | 15 | 0.67(0.62 - 0.73) | 0.68(0.63 - 0.73) | 0.68(0.63 - 0.74) |
| Depression | During Pregnancy | 20 | 0.67(0.62 - 0.72) | 0.67(0.62 - 0.73) | 0.68(0.63 - 0.74) |
| Depression | During Pregnancy | 25 | 0.61(0.57 - 0.66) | 0.62(0.57 - 0.67) | 0.62(0.58 - 0.67) |
| Depression | During Pregnancy | 30 | 0.51(0.47 - 0.55) | 0.51(0.47 - 0.55) | 0.52(0.47 - 0.56) |
| Depression | During Pregnancy | 35 | 0.26(0.23 - 0.29) | 0.26(0.23 - 0.29) | 0.27(0.24 - 0.3) |
| Depression | After Pregnancy | 0 | 0.82(0.76 - 0.88) | 0.83(0.77 - 0.89) | 0.86(0.8 - 0.93) |
| Depression | After Pregnancy | 5 | 1.12(1.04 - 1.19) | 1.13(1.06 - 1.21) | 1.17(1.09 - 1.25) |
| Depression | After Pregnancy | 10 | 1.18(1.1 - 1.26) | 1.2(1.12 - 1.28) | 1.22(1.14 - 1.31) |
| Depression | After Pregnancy | 15 | 1(0.94 - 1.08) | 1.02(0.95 - 1.1) | 1.04(0.97 - 1.11) |
| Depression | After Pregnancy | 20 | 0.89(0.83 - 0.96) | 0.9(0.84 - 0.97) | 0.91(0.85 - 0.98) |
| Depression | After Pregnancy | 25 | 0.88(0.82 - 0.95) | 0.89(0.83 - 0.95) | 0.9(0.84 - 0.96) |
| Depression | After Pregnancy | 30 | 0.91(0.85 - 0.97) | 0.91(0.85 - 0.98) | 0.93(0.86 - 0.99) |
| Depression | After Pregnancy | 35 | 0.94(0.87 - 1.01) | 0.94(0.88 - 1.01) | 0.95(0.88 - 1.02) |
| Depression | After Pregnancy | 40 | 0.96(0.89 - 1.03) | 0.96(0.9 - 1.04) | 0.97(0.9 - 1.04) |
| Depression | After Pregnancy | 45 | 0.96(0.9 - 1.04) | 0.97(0.9 - 1.04) | 0.98(0.91 - 1.05) |
| Depression | After Pregnancy | 50 | 0.95(0.85 - 1.07) | 0.96(0.86 - 1.08) | 0.97(0.86 - 1.09) |
| Anxiety | During Pregnancy | 0 | 0.83(0.78 - 0.89) | 0.84(0.79 - 0.9) | 0.88(0.82 - 0.94) |
| Anxiety | During Pregnancy | 5 | 0.66(0.61 - 0.71) | 0.66(0.61 - 0.71) | 0.68(0.63 - 0.73) |
| Anxiety | During Pregnancy | 10 | 0.56(0.52 - 0.6) | 0.56(0.52 - 0.61) | 0.58(0.53 - 0.62) |
| Anxiety | During Pregnancy | 15 | 0.56(0.51 - 0.6) | 0.56(0.52 - 0.61) | 0.57(0.53 - 0.62) |
| Anxiety | During Pregnancy | 20 | 0.59(0.55 - 0.64) | 0.6(0.56 - 0.65) | 0.61(0.57 - 0.66) |
| Anxiety | During Pregnancy | 25 | 0.55(0.51 - 0.6) | 0.56(0.52 - 0.6) | 0.57(0.53 - 0.61) |
| Anxiety | During Pregnancy | 30 | 0.46(0.43 - 0.5) | 0.47(0.43 - 0.51) | 0.48(0.44 - 0.51) |
| Anxiety | During Pregnancy | 35 | 0.27(0.25 - 0.3) | 0.27(0.25 - 0.3) | 0.28(0.25 - 0.31) |
| Anxiety | After Pregnancy | 0 | 0.5(0.46 - 0.54) | 0.51(0.47 - 0.55) | 0.53(0.49 - 0.58) |
| Anxiety | After Pregnancy | 5 | 0.73(0.68 - 0.78) | 0.74(0.69 - 0.79) | 0.76(0.71 - 0.82) |
| Anxiety | After Pregnancy | 10 | 0.86(0.81 - 0.93) | 0.88(0.82 - 0.94) | 0.9(0.84 - 0.96) |
| Anxiety | After Pregnancy | 15 | 0.78(0.72 - 0.83) | 0.8(0.74 - 0.85) | 0.81(0.76 - 0.87) |
| Anxiety | After Pregnancy | 20 | 0.75(0.7 - 0.8) | 0.76(0.71 - 0.82) | 0.78(0.72 - 0.83) |
| Anxiety | After Pregnancy | 25 | 0.78(0.73 - 0.83) | 0.79(0.74 - 0.85) | 0.8(0.74 - 0.86) |
| Anxiety | After Pregnancy | 30 | 0.78(0.73 - 0.84) | 0.79(0.74 - 0.85) | 0.8(0.75 - 0.86) |
| Anxiety | After Pregnancy | 35 | 0.82(0.76 - 0.87) | 0.82(0.76 - 0.88) | 0.83(0.77 - 0.89) |
| Anxiety | After Pregnancy | 40 | 0.88(0.82 - 0.94) | 0.89(0.83 - 0.95) | 0.89(0.83 - 0.96) |
| Anxiety | After Pregnancy | 45 | 0.94(0.88 - 1.01) | 0.95(0.88 - 1.01) | 0.95(0.89 - 1.02) |
| Anxiety | After Pregnancy | 50 | 0.95(0.85 - 1.06) | 0.96(0.86 - 1.07) | 0.97(0.86 - 1.08) |
| Alcohol Use | During Pregnancy | 0 | 0.51(0.41 - 0.63) | 0.52(0.42 - 0.64) | 0.58(0.47 - 0.71) |
| Alcohol Use | During Pregnancy | 5 | 0.17(0.12 - 0.24) | 0.18(0.13 - 0.25) | 0.19(0.14 - 0.27) |
| Alcohol Use | During Pregnancy | 10 | 0.12(0.09 - 0.17) | 0.12(0.09 - 0.18) | 0.14(0.1 - 0.19) |
| Alcohol Use | During Pregnancy | 15 | 0.18(0.13 - 0.25) | 0.18(0.13 - 0.25) | 0.2(0.14 - 0.27) |
| Alcohol Use | During Pregnancy | 20 | 0.11(0.08 - 0.17) | 0.12(0.08 - 0.17) | 0.12(0.08 - 0.19) |
| Alcohol Use | During Pregnancy | 25 | 0.14(0.09 - 0.2) | 0.14(0.1 - 0.2) | 0.15(0.1 - 0.22) |
| Alcohol Use | During Pregnancy | 30 | 0.05(0.03 - 0.09) | 0.05(0.03 - 0.09) | 0.06(0.03 - 0.1) |
| Alcohol Use | During Pregnancy | 35 | 0.06(0.03 - 0.1) | 0.06(0.03 - 0.1) | 0.06(0.03 - 0.11) |
| Alcohol Use | After Pregnancy | 0 | 0.05(0.03 - 0.09) | 0.06(0.03 - 0.09) | 0.06(0.04 - 0.1) |
| Alcohol Use | After Pregnancy | 5 | 0.11(0.07 - 0.17) | 0.11(0.08 - 0.17) | 0.13(0.08 - 0.19) |
| Alcohol Use | After Pregnancy | 10 | 0.12(0.09 - 0.17) | 0.13(0.09 - 0.18) | 0.14(0.1 - 0.2) |
| Alcohol Use | After Pregnancy | 15 | 0.18(0.13 - 0.25) | 0.18(0.13 - 0.25) | 0.2(0.14 - 0.28) |
| Alcohol Use | After Pregnancy | 20 | 0.19(0.14 - 0.27) | 0.19(0.14 - 0.27) | 0.21(0.15 - 0.29) |
| Alcohol Use | After Pregnancy | 25 | 0.26(0.2 - 0.35) | 0.27(0.2 - 0.36) | 0.29(0.22 - 0.39) |
| Alcohol Use | After Pregnancy | 30 | 0.21(0.15 - 0.28) | 0.21(0.15 - 0.28) | 0.22(0.16 - 0.3) |
| Alcohol Use | After Pregnancy | 35 | 0.27(0.2 - 0.36) | 0.28(0.21 - 0.37) | 0.29(0.22 - 0.39) |
| Alcohol Use | After Pregnancy | 40 | 0.31(0.24 - 0.41) | 0.32(0.24 - 0.42) | 0.33(0.25 - 0.43) |
| Alcohol Use | After Pregnancy | 45 | 0.38(0.29 - 0.5) | 0.38(0.29 - 0.5) | 0.39(0.3 - 0.51) |
| Alcohol Use | After Pregnancy | 50 | 0.56(0.37 - 0.85) | 0.56(0.37 - 0.86) | 0.58(0.38 - 0.88) |
| Other Substance Use | During Pregnancy | 0 | 0.64(0.54 - 0.77) | 0.65(0.54 - 0.77) | 0.71(0.6 - 0.85) |
| Other Substance Use | During Pregnancy | 5 | 0.34(0.28 - 0.42) | 0.35(0.28 - 0.43) | 0.39(0.31 - 0.48) |
| Other Substance Use | During Pregnancy | 10 | 0.37(0.3 - 0.45) | 0.37(0.3 - 0.45) | 0.4(0.33 - 0.49) |
| Other Substance Use | During Pregnancy | 15 | 0.33(0.27 - 0.41) | 0.33(0.27 - 0.42) | 0.36(0.29 - 0.45) |
| Other Substance Use | During Pregnancy | 20 | 0.43(0.35 - 0.54) | 0.44(0.35 - 0.55) | 0.47(0.38 - 0.59) |
| Other Substance Use | During Pregnancy | 25 | 0.28(0.22 - 0.36) | 0.29(0.23 - 0.36) | 0.3(0.24 - 0.39) |
| Other Substance Use | During Pregnancy | 30 | 0.2(0.16 - 0.27) | 0.2(0.16 - 0.27) | 0.21(0.16 - 0.28) |
| Other Substance Use | During Pregnancy | 35 | 0.15(0.11 - 0.21) | 0.16(0.11 - 0.21) | 0.16(0.12 - 0.23) |
| Other Substance Use | After Pregnancy | 0 | 0.21(0.16 - 0.27) | 0.21(0.16 - 0.28) | 0.24(0.18 - 0.31) |
| Other Substance Use | After Pregnancy | 5 | 0.24(0.19 - 0.3) | 0.24(0.19 - 0.31) | 0.27(0.21 - 0.35) |
| Other Substance Use | After Pregnancy | 10 | 0.33(0.26 - 0.4) | 0.33(0.26 - 0.41) | 0.36(0.29 - 0.45) |
| Other Substance Use | After Pregnancy | 15 | 0.41(0.34 - 0.5) | 0.41(0.34 - 0.51) | 0.45(0.37 - 0.55) |
| Other Substance Use | After Pregnancy | 20 | 0.48(0.38 - 0.59) | 0.48(0.39 - 0.6) | 0.52(0.42 - 0.65) |
| Other Substance Use | After Pregnancy | 25 | 0.54(0.44 - 0.65) | 0.54(0.45 - 0.66) | 0.58(0.48 - 0.71) |
| Other Substance Use | After Pregnancy | 30 | 0.43(0.35 - 0.53) | 0.43(0.35 - 0.53) | 0.46(0.37 - 0.56) |
| Other Substance Use | After Pregnancy | 35 | 0.52(0.43 - 0.63) | 0.52(0.43 - 0.63) | 0.55(0.45 - 0.67) |
| Other Substance Use | After Pregnancy | 40 | 0.5(0.41 - 0.61) | 0.51(0.41 - 0.62) | 0.54(0.44 - 0.65) |
| Other Substance Use | After Pregnancy | 45 | 0.61(0.5 - 0.74) | 0.61(0.5 - 0.75) | 0.64(0.52 - 0.78) |
| Other Substance Use | After Pregnancy | 50 | 0.64(0.47 - 0.86) | 0.63(0.47 - 0.86) | 0.65(0.48 - 0.88) |
| Psychosis | During Pregnancy | 0 | 0.79(0.5 - 1.26) | 0.79(0.5 - 1.25) | 0.9(0.57 - 1.43) |
| Psychosis | During Pregnancy | 5 | 0.59(0.34 - 1.01) | 0.56(0.32 - 0.96) | 0.59(0.34 - 1.01) |
| Psychosis | During Pregnancy | 10 | 0.39(0.22 - 0.67) | 0.38(0.22 - 0.66) | 0.39(0.23 - 0.68) |
| Psychosis | During Pregnancy | 15 | 1.02(0.6 - 1.74) | 0.99(0.58 - 1.69) | 1.01(0.59 - 1.72) |
| Psychosis | During Pregnancy | 20 | 0.59(0.37 - 0.95) | 0.57(0.35 - 0.91) | 0.59(0.37 - 0.95) |
| Psychosis | During Pregnancy | 25 | 0.48(0.3 - 0.77) | 0.46(0.29 - 0.74) | 0.47(0.29 - 0.76) |
| Psychosis | During Pregnancy | 30 | 0.45(0.27 - 0.74) | 0.44(0.27 - 0.72) | 0.46(0.28 - 0.75) |
| Psychosis | During Pregnancy | 35 | 0.68(0.36 - 1.29) | 0.63(0.33 - 1.19) | 0.63(0.34 - 1.2) |
| Psychosis | After Pregnancy | 0 | 5.71(4.02 - 8.12) | 5.67(3.98 - 8.06) | 6.43(4.52 - 9.16) |
| Psychosis | After Pregnancy | 5 | 3.65(2.46 - 5.41) | 3.4(2.29 - 5.04) | 3.58(2.41 - 5.32) |
| Psychosis | After Pregnancy | 10 | 2.3(1.59 - 3.33) | 2.24(1.55 - 3.24) | 2.33(1.61 - 3.37) |
| Psychosis | After Pregnancy | 15 | 3.41(2.19 - 5.33) | 3.34(2.13 - 5.21) | 3.38(2.16 - 5.29) |
| Psychosis | After Pregnancy | 20 | 1.29(0.87 - 1.91) | 1.23(0.83 - 1.83) | 1.29(0.87 - 1.92) |
| Psychosis | After Pregnancy | 25 | 1.27(0.87 - 1.84) | 1.22(0.84 - 1.78) | 1.25(0.86 - 1.82) |
| Psychosis | After Pregnancy | 30 | 1.18(0.8 - 1.74) | 1.17(0.79 - 1.72) | 1.21(0.82 - 1.79) |
| Psychosis | After Pregnancy | 35 | 1.71(1.04 - 2.82) | 1.59(0.96 - 2.61) | 1.58(0.96 - 2.61) |
| Psychosis | After Pregnancy | 40 | 1.07(0.7 - 1.62) | 1.01(0.67 - 1.54) | 1.03(0.67 - 1.56) |
| Psychosis | After Pregnancy | 45 | 1.12(0.71 - 1.77) | 1.03(0.65 - 1.63) | 1.05(0.66 - 1.66) |
| Psychosis | After Pregnancy | 50 | 0.85(0.38 - 1.9) | 0.8(0.36 - 1.78) | 0.84(0.37 - 1.89) |
| Bipolar | During Pregnancy | 0 | 0.82(0.64 - 1.06) | 0.83(0.64 - 1.07) | 0.88(0.68 - 1.14) |
| Bipolar | During Pregnancy | 5 | 0.64(0.49 - 0.82) | 0.65(0.5 - 0.83) | 0.68(0.52 - 0.87) |
| Bipolar | During Pregnancy | 10 | 0.67(0.49 - 0.92) | 0.69(0.5 - 0.94) | 0.72(0.52 - 0.98) |
| Bipolar | During Pregnancy | 15 | 0.38(0.28 - 0.51) | 0.39(0.29 - 0.52) | 0.4(0.3 - 0.54) |
| Bipolar | During Pregnancy | 20 | 0.58(0.42 - 0.78) | 0.58(0.43 - 0.79) | 0.58(0.43 - 0.79) |
| Bipolar | During Pregnancy | 25 | 0.49(0.37 - 0.66) | 0.5(0.37 - 0.67) | 0.51(0.38 - 0.69) |
| Bipolar | During Pregnancy | 30 | 0.47(0.34 - 0.63) | 0.47(0.35 - 0.64) | 0.48(0.35 - 0.64) |
| Bipolar | During Pregnancy | 35 | 0.3(0.2 - 0.44) | 0.3(0.21 - 0.45) | 0.31(0.21 - 0.46) |
| Bipolar | After Pregnancy | 0 | 0.6(0.45 - 0.79) | 0.61(0.46 - 0.8) | 0.64(0.49 - 0.85) |
| Bipolar | After Pregnancy | 5 | 0.48(0.36 - 0.63) | 0.49(0.38 - 0.65) | 0.52(0.39 - 0.68) |
| Bipolar | After Pregnancy | 10 | 0.86(0.64 - 1.16) | 0.89(0.66 - 1.2) | 0.93(0.69 - 1.26) |
| Bipolar | After Pregnancy | 15 | 0.47(0.35 - 0.61) | 0.47(0.36 - 0.62) | 0.49(0.37 - 0.65) |
| Bipolar | After Pregnancy | 20 | 0.98(0.75 - 1.29) | 1.01(0.78 - 1.33) | 1.01(0.77 - 1.32) |
| Bipolar | After Pregnancy | 25 | 0.72(0.55 - 0.95) | 0.74(0.56 - 0.96) | 0.76(0.58 - 0.99) |
| Bipolar | After Pregnancy | 30 | 0.75(0.57 - 0.98) | 0.76(0.58 - 1) | 0.77(0.59 - 1.01) |
| Bipolar | After Pregnancy | 35 | 0.8(0.6 - 1.05) | 0.8(0.61 - 1.06) | 0.82(0.62 - 1.08) |
| Bipolar | After Pregnancy | 40 | 1(0.75 - 1.32) | 0.99(0.75 - 1.3) | 1(0.76 - 1.32) |
| Bipolar | After Pregnancy | 45 | 0.96(0.74 - 1.25) | 0.97(0.75 - 1.26) | 0.97(0.75 - 1.26) |
| Bipolar | After Pregnancy | 50 | 1.19(0.8 - 1.78) | 1.19(0.8 - 1.77) | 1.2(0.8 - 1.79) |
| Stress-Related | During Pregnancy | 0 | 0.95(0.89 - 1.02) | 0.96(0.9 - 1.02) | 0.99(0.93 - 1.06) |
| Stress-Related | During Pregnancy | 5 | 0.77(0.72 - 0.82) | 0.77(0.72 - 0.83) | 0.79(0.74 - 0.85) |
| Stress-Related | During Pregnancy | 10 | 0.7(0.65 - 0.75) | 0.7(0.65 - 0.75) | 0.71(0.66 - 0.76) |
| Stress-Related | During Pregnancy | 15 | 0.66(0.61 - 0.71) | 0.66(0.62 - 0.71) | 0.67(0.63 - 0.72) |
| Stress-Related | During Pregnancy | 20 | 0.74(0.69 - 0.8) | 0.75(0.7 - 0.8) | 0.76(0.71 - 0.81) |
| Stress-Related | During Pregnancy | 25 | 0.7(0.65 - 0.75) | 0.7(0.65 - 0.75) | 0.71(0.66 - 0.76) |
| Stress-Related | During Pregnancy | 30 | 0.53(0.49 - 0.57) | 0.53(0.49 - 0.57) | 0.53(0.5 - 0.58) |
| Stress-Related | During Pregnancy | 35 | 0.23(0.2 - 0.25) | 0.23(0.2 - 0.25) | 0.23(0.21 - 0.26) |
| Stress-Related | After Pregnancy | 0 | 0.59(0.55 - 0.64) | 0.6(0.56 - 0.65) | 0.63(0.58 - 0.67) |
| Stress-Related | After Pregnancy | 5 | 0.76(0.71 - 0.81) | 0.77(0.72 - 0.82) | 0.79(0.73 - 0.85) |
| Stress-Related | After Pregnancy | 10 | 0.71(0.66 - 0.76) | 0.72(0.67 - 0.77) | 0.73(0.68 - 0.78) |
| Stress-Related | After Pregnancy | 15 | 0.61(0.57 - 0.66) | 0.62(0.58 - 0.67) | 0.63(0.59 - 0.68) |
| Stress-Related | After Pregnancy | 20 | 0.59(0.55 - 0.64) | 0.6(0.56 - 0.65) | 0.61(0.56 - 0.65) |
| Stress-Related | After Pregnancy | 25 | 0.57(0.53 - 0.62) | 0.57(0.53 - 0.62) | 0.58(0.54 - 0.63) |
| Stress-Related | After Pregnancy | 30 | 0.6(0.55 - 0.64) | 0.6(0.56 - 0.65) | 0.6(0.56 - 0.65) |
| Stress-Related | After Pregnancy | 35 | 0.61(0.56 - 0.65) | 0.61(0.56 - 0.65) | 0.61(0.57 - 0.66) |
| Stress-Related | After Pregnancy | 40 | 0.58(0.54 - 0.63) | 0.58(0.54 - 0.63) | 0.59(0.55 - 0.63) |
| Stress-Related | After Pregnancy | 45 | 0.69(0.64 - 0.74) | 0.69(0.65 - 0.74) | 0.7(0.65 - 0.75) |
| Stress-Related | After Pregnancy | 50 | 0.72(0.64 - 0.8) | 0.72(0.65 - 0.81) | 0.72(0.65 - 0.81) |

IRR = incidence rate ratio; CI = confidence intervals

Model 1: adjusted for age and calendar year at delivery and week at follow-up

Model 2: additionally adjusted for country of birth, region of residence, education, and season at follow-up

Model 3: additionally adjusted for civil status, smoking, BMI category, multiple gestation, hypertensive disease, diabetes, and parity.

**Supplementary Table 5** Standardized incidence rate and incidence rate ratio of any psychiatric disorder and depression, stratified on year of delivery.

| **Psychiatric disorder** | **Calendar year** | **Phase** | **Week** | **SIR** | **95% CI** | | **IRR** | **95% CI** | |
| --- | --- | --- | --- | --- | --- | --- | --- | --- | --- |
| Any | 2003-2010 | Before Pregnancy | 0 | 10.83 | 9.43 | 12.23 | Ref. | Ref. | Ref. |
| Any | 2003-2010 | Before Pregnancy | 1 | 10.01 | 8.66 | 11.36 |  |  |  |
| Any | 2003-2010 | Before Pregnancy | 2 | 9.87 | 8.53 | 11.21 |  |  |  |
| Any | 2003-2010 | Before Pregnancy | 3 | 9.89 | 8.56 | 11.23 |  |  |  |
| Any | 2003-2010 | Before Pregnancy | 4 | 10.49 | 9.11 | 11.86 |  |  |  |
| Any | 2003-2010 | Before Pregnancy | 5 | 10.20 | 8.84 | 11.55 | Ref. | Ref. | Ref. |
| Any | 2003-2010 | Before Pregnancy | 6 | 10.99 | 9.59 | 12.39 |  |  |  |
| Any | 2003-2010 | Before Pregnancy | 7 | 9.81 | 8.49 | 11.13 |  |  |  |
| Any | 2003-2010 | Before Pregnancy | 8 | 10.01 | 8.68 | 11.35 |  |  |  |
| Any | 2003-2010 | Before Pregnancy | 9 | 10.99 | 9.60 | 12.39 |  |  |  |
| Any | 2003-2010 | Before Pregnancy | 10 | 10.38 | 9.03 | 11.74 | Ref. | Ref. | Ref. |
| Any | 2003-2010 | Before Pregnancy | 11 | 10.66 | 9.29 | 12.02 |  |  |  |
| Any | 2003-2010 | Before Pregnancy | 12 | 9.72 | 8.42 | 11.02 |  |  |  |
| Any | 2003-2010 | Before Pregnancy | 13 | 10.21 | 8.88 | 11.55 |  |  |  |
| Any | 2003-2010 | Before Pregnancy | 14 | 9.93 | 8.61 | 11.25 |  |  |  |
| Any | 2003-2010 | Before Pregnancy | 15 | 11.42 | 10.01 | 12.83 | Ref. | Ref. | Ref. |
| Any | 2003-2010 | Before Pregnancy | 16 | 10.36 | 9.02 | 11.70 |  |  |  |
| Any | 2003-2010 | Before Pregnancy | 17 | 10.35 | 9.01 | 11.68 |  |  |  |
| Any | 2003-2010 | Before Pregnancy | 18 | 10.38 | 9.05 | 11.72 |  |  |  |
| Any | 2003-2010 | Before Pregnancy | 19 | 9.59 | 8.31 | 10.88 |  |  |  |
| Any | 2003-2010 | Before Pregnancy | 20 | 9.86 | 8.56 | 11.15 | Ref. | Ref. | Ref. |
| Any | 2003-2010 | Before Pregnancy | 21 | 11.07 | 9.70 | 12.45 |  |  |  |
| Any | 2003-2010 | Before Pregnancy | 22 | 11.52 | 10.12 | 12.92 |  |  |  |
| Any | 2003-2010 | Before Pregnancy | 23 | 10.04 | 8.73 | 11.34 |  |  |  |
| Any | 2003-2010 | Before Pregnancy | 24 | 11.19 | 9.82 | 12.56 |  |  |  |
| Any | 2003-2010 | Before Pregnancy | 25 | 9.93 | 8.64 | 11.22 | Ref. | Ref. | Ref. |
| Any | 2003-2010 | Before Pregnancy | 26 | 11.90 | 10.49 | 13.31 |  |  |  |
| Any | 2003-2010 | Before Pregnancy | 27 | 10.60 | 9.27 | 11.93 |  |  |  |
| Any | 2003-2010 | Before Pregnancy | 28 | 11.12 | 9.76 | 12.48 |  |  |  |
| Any | 2003-2010 | Before Pregnancy | 29 | 11.61 | 10.22 | 13.00 |  |  |  |
| Any | 2003-2010 | Before Pregnancy | 30 | 11.19 | 9.83 | 12.55 | Ref. | Ref. | Ref. |
| Any | 2003-2010 | Before Pregnancy | 31 | 11.67 | 10.28 | 13.06 |  |  |  |
| Any | 2003-2010 | Before Pregnancy | 32 | 10.68 | 9.36 | 12.00 |  |  |  |
| Any | 2003-2010 | Before Pregnancy | 33 | 9.97 | 8.69 | 11.25 |  |  |  |
| Any | 2003-2010 | Before Pregnancy | 34 | 8.74 | 7.55 | 9.93 |  |  |  |
| Any | 2003-2010 | Before Pregnancy | 35 | 11.18 | 9.83 | 12.53 | Ref. | Ref. | Ref. |
| Any | 2003-2010 | Before Pregnancy | 36 | 11.09 | 9.75 | 12.43 |  |  |  |
| Any | 2003-2010 | Before Pregnancy | 37 | 9.65 | 8.40 | 10.89 |  |  |  |
| Any | 2003-2010 | Before Pregnancy | 38 | 10.78 | 9.46 | 12.10 |  |  |  |
| Any | 2003-2010 | Before Pregnancy | 39 | 10.26 | 8.98 | 11.55 |  |  |  |
| Any | 2003-2010 | Before Pregnancy | 40 | 9.97 | 8.71 | 11.24 | Ref. | Ref. | Ref. |
| Any | 2003-2010 | Before Pregnancy | 41 | 11.22 | 9.88 | 12.56 |  |  |  |
| Any | 2003-2010 | Before Pregnancy | 42 | 9.81 | 8.56 | 11.05 |  |  |  |
| Any | 2003-2010 | Before Pregnancy | 43 | 9.26 | 8.05 | 10.47 |  |  |  |
| Any | 2003-2010 | Before Pregnancy | 44 | 11.36 | 10.02 | 12.70 |  |  |  |
| Any | 2003-2010 | Before Pregnancy | 45 | 10.30 | 9.02 | 11.57 | Ref. | Ref. | Ref. |
| Any | 2003-2010 | Before Pregnancy | 46 | 11.03 | 9.72 | 12.35 |  |  |  |
| Any | 2003-2010 | Before Pregnancy | 47 | 11.56 | 10.22 | 12.91 |  |  |  |
| Any | 2003-2010 | Before Pregnancy | 48 | 9.59 | 8.37 | 10.81 |  |  |  |
| Any | 2003-2010 | Before Pregnancy | 49 | 10.25 | 8.99 | 11.51 |  |  |  |
| Any | 2003-2010 | Before Pregnancy | 50 | 9.74 | 8.52 | 10.97 | Ref. | Ref. | Ref. |
| Any | 2003-2010 | Before Pregnancy | 51 | 10.41 | 9.15 | 11.68 |  |  |  |
| Any | 2003-2010 | During Pregnancy | 0 | 11.37 | 10.10 | 12.63 | 0.93 | 0.86 | 1.01 |
| Any | 2003-2010 | During Pregnancy | 1 | 8.73 | 7.62 | 9.83 |  |  |  |
| Any | 2003-2010 | During Pregnancy | 2 | 8.21 | 7.14 | 9.28 |  |  |  |
| Any | 2003-2010 | During Pregnancy | 3 | 8.57 | 7.48 | 9.67 |  |  |  |
| Any | 2003-2010 | During Pregnancy | 4 | 7.69 | 6.65 | 8.73 |  |  |  |
| Any | 2003-2010 | During Pregnancy | 5 | 7.51 | 6.48 | 8.53 | 0.65 | 0.59 | 0.71 |
| Any | 2003-2010 | During Pregnancy | 6 | 7.25 | 6.24 | 8.26 |  |  |  |
| Any | 2003-2010 | During Pregnancy | 7 | 6.01 | 5.09 | 6.93 |  |  |  |
| Any | 2003-2010 | During Pregnancy | 8 | 5.90 | 4.99 | 6.81 |  |  |  |
| Any | 2003-2010 | During Pregnancy | 9 | 5.46 | 4.59 | 6.34 |  |  |  |
| Any | 2003-2010 | During Pregnancy | 10 | 5.61 | 4.72 | 6.49 | 0.62 | 0.57 | 0.68 |
| Any | 2003-2010 | During Pregnancy | 11 | 6.12 | 5.19 | 7.04 |  |  |  |
| Any | 2003-2010 | During Pregnancy | 12 | 5.97 | 5.06 | 6.89 |  |  |  |
| Any | 2003-2010 | During Pregnancy | 13 | 6.41 | 5.46 | 7.35 |  |  |  |
| Any | 2003-2010 | During Pregnancy | 14 | 6.29 | 5.36 | 7.23 |  |  |  |
| Any | 2003-2010 | During Pregnancy | 15 | 6.04 | 5.12 | 6.96 | 0.57 | 0.52 | 0.63 |
| Any | 2003-2010 | During Pregnancy | 16 | 5.78 | 4.88 | 6.68 |  |  |  |
| Any | 2003-2010 | During Pregnancy | 17 | 5.85 | 4.95 | 6.76 |  |  |  |
| Any | 2003-2010 | During Pregnancy | 18 | 5.56 | 4.68 | 6.44 |  |  |  |
| Any | 2003-2010 | During Pregnancy | 19 | 5.56 | 4.68 | 6.44 |  |  |  |
| Any | 2003-2010 | During Pregnancy | 20 | 6.03 | 5.11 | 6.95 | 0.56 | 0.51 | 0.61 |
| Any | 2003-2010 | During Pregnancy | 21 | 5.96 | 5.04 | 6.87 |  |  |  |
| Any | 2003-2010 | During Pregnancy | 22 | 5.45 | 4.58 | 6.32 |  |  |  |
| Any | 2003-2010 | During Pregnancy | 23 | 5.88 | 4.98 | 6.79 |  |  |  |
| Any | 2003-2010 | During Pregnancy | 24 | 5.45 | 4.58 | 6.32 |  |  |  |
| Any | 2003-2010 | During Pregnancy | 25 | 5.70 | 4.81 | 6.59 | 0.52 | 0.48 | 0.57 |
| Any | 2003-2010 | During Pregnancy | 26 | 5.88 | 4.98 | 6.79 |  |  |  |
| Any | 2003-2010 | During Pregnancy | 27 | 5.23 | 4.38 | 6.09 |  |  |  |
| Any | 2003-2010 | During Pregnancy | 28 | 5.56 | 4.68 | 6.45 |  |  |  |
| Any | 2003-2010 | During Pregnancy | 29 | 5.28 | 4.42 | 6.14 |  |  |  |
| Any | 2003-2010 | During Pregnancy | 30 | 4.55 | 3.76 | 5.35 | 0.43 | 0.39 | 0.47 |
| Any | 2003-2010 | During Pregnancy | 31 | 4.23 | 3.46 | 5.00 |  |  |  |
| Any | 2003-2010 | During Pregnancy | 32 | 4.50 | 3.70 | 5.29 |  |  |  |
| Any | 2003-2010 | During Pregnancy | 33 | 4.26 | 3.48 | 5.03 |  |  |  |
| Any | 2003-2010 | During Pregnancy | 34 | 4.06 | 3.30 | 4.82 |  |  |  |
| Any | 2003-2010 | During Pregnancy | 35 | 3.16 | 2.49 | 3.84 | 0.23 | 0.20 | 0.26 |
| Any | 2003-2010 | During Pregnancy | 36 | 2.69 | 2.07 | 3.32 |  |  |  |
| Any | 2003-2010 | During Pregnancy | 37 | 2.38 | 1.78 | 2.98 |  |  |  |
| Any | 2003-2010 | During Pregnancy | 38 | 1.89 | 1.32 | 2.46 |  |  |  |
| Any | 2003-2010 | During Pregnancy | 39 | 1.09 | 0.58 | 1.59 |  |  |  |
| Any | 2003-2010 | During Pregnancy | 40 | 1.44 | 0.66 | 2.22 | - | - | |
| Any | 2003-2010 | During Pregnancy | 41 | 0.98 | 0.00 | 2.09 |  |  |  |
| Any | 2003-2010 | After Pregnancy | 0 | 5.99 | 5.06 | 6.91 | 0.79 | 0.72 | 0.86 |
| Any | 2003-2010 | After Pregnancy | 1 | 7.94 | 6.88 | 9.01 |  |  |  |
| Any | 2003-2010 | After Pregnancy | 2 | 8.17 | 7.09 | 9.25 |  |  |  |
| Any | 2003-2010 | After Pregnancy | 3 | 8.06 | 6.98 | 9.13 |  |  |  |
| Any | 2003-2010 | After Pregnancy | 4 | 8.41 | 7.31 | 9.51 |  |  |  |
| Any | 2003-2010 | After Pregnancy | 5 | 8.28 | 7.19 | 9.37 | 0.83 | 0.77 | 0.90 |
| Any | 2003-2010 | After Pregnancy | 6 | 9.16 | 8.01 | 10.31 |  |  |  |
| Any | 2003-2010 | After Pregnancy | 7 | 8.07 | 6.99 | 9.14 |  |  |  |
| Any | 2003-2010 | After Pregnancy | 8 | 8.75 | 7.63 | 9.87 |  |  |  |
| Any | 2003-2010 | After Pregnancy | 9 | 8.04 | 6.97 | 9.12 |  |  |  |
| Any | 2003-2010 | After Pregnancy | 10 | 8.64 | 7.53 | 9.76 | 0.95 | 0.88 | 1.03 |
| Any | 2003-2010 | After Pregnancy | 11 | 9.22 | 8.07 | 10.37 |  |  |  |
| Any | 2003-2010 | After Pregnancy | 12 | 10.27 | 9.06 | 11.49 |  |  |  |
| Any | 2003-2010 | After Pregnancy | 13 | 10.06 | 8.86 | 11.27 |  |  |  |
| Any | 2003-2010 | After Pregnancy | 14 | 9.29 | 8.13 | 10.45 |  |  |  |
| Any | 2003-2010 | After Pregnancy | 15 | 9.18 | 8.03 | 10.34 | 0.86 | 0.80 | 0.94 |
| Any | 2003-2010 | After Pregnancy | 16 | 7.80 | 6.74 | 8.86 |  |  |  |
| Any | 2003-2010 | After Pregnancy | 17 | 8.94 | 7.80 | 10.08 |  |  |  |
| Any | 2003-2010 | After Pregnancy | 18 | 9.49 | 8.31 | 10.66 |  |  |  |
| Any | 2003-2010 | After Pregnancy | 19 | 8.66 | 7.54 | 9.79 |  |  |  |
| Any | 2003-2010 | After Pregnancy | 20 | 7.88 | 6.81 | 8.95 | 0.79 | 0.73 | 0.86 |
| Any | 2003-2010 | After Pregnancy | 21 | 8.77 | 7.64 | 9.90 |  |  |  |
| Any | 2003-2010 | After Pregnancy | 22 | 8.63 | 7.50 | 9.75 |  |  |  |
| Any | 2003-2010 | After Pregnancy | 23 | 7.61 | 6.56 | 8.67 |  |  |  |
| Any | 2003-2010 | After Pregnancy | 24 | 8.81 | 7.67 | 9.94 |  |  |  |
| Any | 2003-2010 | After Pregnancy | 25 | 8.25 | 7.15 | 9.35 | 0.83 | 0.76 | 0.89 |
| Any | 2003-2010 | After Pregnancy | 26 | 9.80 | 8.60 | 11.00 |  |  |  |
| Any | 2003-2010 | After Pregnancy | 27 | 9.13 | 7.97 | 10.28 |  |  |  |
| Any | 2003-2010 | After Pregnancy | 28 | 9.41 | 8.23 | 10.59 |  |  |  |
| Any | 2003-2010 | After Pregnancy | 29 | 8.47 | 7.35 | 9.59 |  |  |  |
| Any | 2003-2010 | After Pregnancy | 30 | 8.03 | 6.94 | 9.12 | 0.86 | 0.79 | 0.93 |
| Any | 2003-2010 | After Pregnancy | 31 | 8.58 | 7.45 | 9.71 |  |  |  |
| Any | 2003-2010 | After Pregnancy | 32 | 9.34 | 8.16 | 10.52 |  |  |  |
| Any | 2003-2010 | After Pregnancy | 33 | 9.56 | 8.37 | 10.76 |  |  |  |
| Any | 2003-2010 | After Pregnancy | 34 | 8.84 | 7.69 | 9.99 |  |  |  |
| Any | 2003-2010 | After Pregnancy | 35 | 8.47 | 7.34 | 9.60 | 0.81 | 0.75 | 0.88 |
| Any | 2003-2010 | After Pregnancy | 36 | 8.70 | 7.55 | 9.84 |  |  |  |
| Any | 2003-2010 | After Pregnancy | 37 | 8.29 | 7.17 | 9.40 |  |  |  |
| Any | 2003-2010 | After Pregnancy | 38 | 8.11 | 7.00 | 9.22 |  |  |  |
| Any | 2003-2010 | After Pregnancy | 39 | 9.09 | 7.91 | 10.26 |  |  |  |
| Any | 2003-2010 | After Pregnancy | 40 | 9.15 | 7.97 | 10.33 | 0.89 | 0.82 | 0.96 |
| Any | 2003-2010 | After Pregnancy | 41 | 9.19 | 8.01 | 10.38 |  |  |  |
| Any | 2003-2010 | After Pregnancy | 42 | 8.26 | 7.13 | 9.38 |  |  |  |
| Any | 2003-2010 | After Pregnancy | 43 | 10.04 | 8.80 | 11.28 |  |  |  |
| Any | 2003-2010 | After Pregnancy | 44 | 8.78 | 7.62 | 9.94 |  |  |  |
| Any | 2003-2010 | After Pregnancy | 45 | 8.76 | 7.59 | 9.92 | 0.92 | 0.85 | 1.00 |
| Any | 2003-2010 | After Pregnancy | 46 | 10.07 | 8.82 | 11.32 |  |  |  |
| Any | 2003-2010 | After Pregnancy | 47 | 9.80 | 8.56 | 11.03 |  |  |  |
| Any | 2003-2010 | After Pregnancy | 48 | 10.22 | 8.96 | 11.49 |  |  |  |
| Any | 2003-2010 | After Pregnancy | 49 | 9.61 | 8.38 | 10.83 |  |  |  |
| Any | 2003-2010 | After Pregnancy | 50 | 10.05 | 8.79 | 11.31 | 0.98 | 0.87 | 1.11 |
| Any | 2003-2010 | After Pregnancy | 51 | 9.68 | 8.52 | 10.84 |  |  |  |
| Any | 2011-2019 | Before Pregnancy | 0 | 18.50 | 16.64 | 20.36 | Ref. | Ref. | Ref. |
| Any | 2011-2019 | Before Pregnancy | 1 | 16.52 | 14.76 | 18.27 |  |  |  |
| Any | 2011-2019 | Before Pregnancy | 2 | 14.55 | 12.91 | 16.19 |  |  |  |
| Any | 2011-2019 | Before Pregnancy | 3 | 17.78 | 15.97 | 19.59 |  |  |  |
| Any | 2011-2019 | Before Pregnancy | 4 | 16.16 | 14.44 | 17.89 |  |  |  |
| Any | 2011-2019 | Before Pregnancy | 5 | 16.44 | 14.71 | 18.18 | Ref. | Ref. | Ref. |
| Any | 2011-2019 | Before Pregnancy | 6 | 17.03 | 15.27 | 18.79 |  |  |  |
| Any | 2011-2019 | Before Pregnancy | 7 | 17.12 | 15.36 | 18.88 |  |  |  |
| Any | 2011-2019 | Before Pregnancy | 8 | 15.34 | 13.68 | 17.01 |  |  |  |
| Any | 2011-2019 | Before Pregnancy | 9 | 16.59 | 14.86 | 18.31 |  |  |  |
| Any | 2011-2019 | Before Pregnancy | 10 | 16.15 | 14.45 | 17.85 | Ref. | Ref. | Ref. |
| Any | 2011-2019 | Before Pregnancy | 11 | 15.13 | 13.49 | 16.77 |  |  |  |
| Any | 2011-2019 | Before Pregnancy | 12 | 14.42 | 12.82 | 16.02 |  |  |  |
| Any | 2011-2019 | Before Pregnancy | 13 | 14.79 | 13.18 | 16.41 |  |  |  |
| Any | 2011-2019 | Before Pregnancy | 14 | 15.93 | 14.26 | 17.61 |  |  |  |
| Any | 2011-2019 | Before Pregnancy | 15 | 15.61 | 13.95 | 17.26 | Ref. | Ref. | Ref. |
| Any | 2011-2019 | Before Pregnancy | 16 | 16.22 | 14.54 | 17.90 |  |  |  |
| Any | 2011-2019 | Before Pregnancy | 17 | 16.11 | 14.44 | 17.79 |  |  |  |
| Any | 2011-2019 | Before Pregnancy | 18 | 16.31 | 14.63 | 17.99 |  |  |  |
| Any | 2011-2019 | Before Pregnancy | 19 | 15.12 | 13.50 | 16.73 |  |  |  |
| Any | 2011-2019 | Before Pregnancy | 20 | 16.44 | 14.76 | 18.12 | Ref. | Ref. | Ref. |
| Any | 2011-2019 | Before Pregnancy | 21 | 15.43 | 13.81 | 17.06 |  |  |  |
| Any | 2011-2019 | Before Pregnancy | 22 | 15.37 | 13.75 | 16.98 |  |  |  |
| Any | 2011-2019 | Before Pregnancy | 23 | 15.39 | 13.78 | 17.01 |  |  |  |
| Any | 2011-2019 | Before Pregnancy | 24 | 16.51 | 14.84 | 18.18 |  |  |  |
| Any | 2011-2019 | Before Pregnancy | 25 | 16.00 | 14.36 | 17.64 | Ref. | Ref. | Ref. |
| Any | 2011-2019 | Before Pregnancy | 26 | 14.97 | 13.39 | 16.55 |  |  |  |
| Any | 2011-2019 | Before Pregnancy | 27 | 15.87 | 14.24 | 17.49 |  |  |  |
| Any | 2011-2019 | Before Pregnancy | 28 | 15.49 | 13.89 | 17.09 |  |  |  |
| Any | 2011-2019 | Before Pregnancy | 29 | 14.78 | 13.22 | 16.35 |  |  |  |
| Any | 2011-2019 | Before Pregnancy | 30 | 16.00 | 14.37 | 17.62 | Ref. | Ref. | Ref. |
| Any | 2011-2019 | Before Pregnancy | 31 | 15.51 | 13.92 | 17.11 |  |  |  |
| Any | 2011-2019 | Before Pregnancy | 32 | 14.42 | 12.88 | 15.95 |  |  |  |
| Any | 2011-2019 | Before Pregnancy | 33 | 15.03 | 13.47 | 16.59 |  |  |  |
| Any | 2011-2019 | Before Pregnancy | 34 | 13.83 | 12.34 | 15.32 |  |  |  |
| Any | 2011-2019 | Before Pregnancy | 35 | 15.10 | 13.55 | 16.66 | Ref. | Ref. | Ref. |
| Any | 2011-2019 | Before Pregnancy | 36 | 16.25 | 14.64 | 17.87 |  |  |  |
| Any | 2011-2019 | Before Pregnancy | 37 | 14.62 | 13.09 | 16.14 |  |  |  |
| Any | 2011-2019 | Before Pregnancy | 38 | 16.89 | 15.26 | 18.53 |  |  |  |
| Any | 2011-2019 | Before Pregnancy | 39 | 16.55 | 14.94 | 18.17 |  |  |  |
| Any | 2011-2019 | Before Pregnancy | 40 | 15.86 | 14.28 | 17.43 | Ref. | Ref. | Ref. |
| Any | 2011-2019 | Before Pregnancy | 41 | 14.68 | 13.17 | 16.20 |  |  |  |
| Any | 2011-2019 | Before Pregnancy | 42 | 14.90 | 13.38 | 16.42 |  |  |  |
| Any | 2011-2019 | Before Pregnancy | 43 | 14.74 | 13.22 | 16.25 |  |  |  |
| Any | 2011-2019 | Before Pregnancy | 44 | 14.23 | 12.75 | 15.71 |  |  |  |
| Any | 2011-2019 | Before Pregnancy | 45 | 15.17 | 13.64 | 16.70 | Ref. | Ref. | Ref. |
| Any | 2011-2019 | Before Pregnancy | 46 | 14.26 | 12.79 | 15.74 |  |  |  |
| Any | 2011-2019 | Before Pregnancy | 47 | 15.22 | 13.70 | 16.75 |  |  |  |
| Any | 2011-2019 | Before Pregnancy | 48 | 15.49 | 13.96 | 17.03 |  |  |  |
| Any | 2011-2019 | Before Pregnancy | 49 | 15.26 | 13.74 | 16.78 |  |  |  |
| Any | 2011-2019 | Before Pregnancy | 50 | 13.88 | 12.43 | 15.32 | Ref. | Ref. | Ref. |
| Any | 2011-2019 | Before Pregnancy | 51 | 14.24 | 12.78 | 15.70 |  |  |  |
| Any | 2011-2019 | During Pregnancy | 0 | 16.70 | 15.17 | 18.24 | 0.82 | 0.77 | 0.88 |
| Any | 2011-2019 | During Pregnancy | 1 | 14.02 | 12.61 | 15.42 |  |  |  |
| Any | 2011-2019 | During Pregnancy | 2 | 11.77 | 10.49 | 13.06 |  |  |  |
| Any | 2011-2019 | During Pregnancy | 3 | 13.20 | 11.84 | 14.56 |  |  |  |
| Any | 2011-2019 | During Pregnancy | 4 | 11.14 | 9.89 | 12.39 |  |  |  |
| Any | 2011-2019 | During Pregnancy | 5 | 10.36 | 9.16 | 11.57 | 0.60 | 0.56 | 0.64 |
| Any | 2011-2019 | During Pregnancy | 6 | 10.55 | 9.33 | 11.76 |  |  |  |
| Any | 2011-2019 | During Pregnancy | 7 | 9.67 | 8.50 | 10.83 |  |  |  |
| Any | 2011-2019 | During Pregnancy | 8 | 9.52 | 8.37 | 10.68 |  |  |  |
| Any | 2011-2019 | During Pregnancy | 9 | 9.37 | 8.23 | 10.52 |  |  |  |
| Any | 2011-2019 | During Pregnancy | 10 | 9.44 | 8.29 | 10.59 | 0.61 | 0.57 | 0.66 |
| Any | 2011-2019 | During Pregnancy | 11 | 9.48 | 8.33 | 10.63 |  |  |  |
| Any | 2011-2019 | During Pregnancy | 12 | 8.42 | 7.33 | 9.51 |  |  |  |
| Any | 2011-2019 | During Pregnancy | 13 | 9.26 | 8.12 | 10.39 |  |  |  |
| Any | 2011-2019 | During Pregnancy | 14 | 10.67 | 9.45 | 11.90 |  |  |  |
| Any | 2011-2019 | During Pregnancy | 15 | 9.61 | 8.45 | 10.77 | 0.65 | 0.60 | 0.69 |
| Any | 2011-2019 | During Pregnancy | 16 | 10.60 | 9.38 | 11.81 |  |  |  |
| Any | 2011-2019 | During Pregnancy | 17 | 10.99 | 9.75 | 12.23 |  |  |  |
| Any | 2011-2019 | During Pregnancy | 18 | 10.63 | 9.41 | 11.85 |  |  |  |
| Any | 2011-2019 | During Pregnancy | 19 | 10.04 | 8.85 | 11.22 |  |  |  |
| Any | 2011-2019 | During Pregnancy | 20 | 10.94 | 9.71 | 12.18 | 0.69 | 0.64 | 0.74 |
| Any | 2011-2019 | During Pregnancy | 21 | 10.54 | 9.33 | 11.76 |  |  |  |
| Any | 2011-2019 | During Pregnancy | 22 | 11.23 | 9.98 | 12.49 |  |  |  |
| Any | 2011-2019 | During Pregnancy | 23 | 9.89 | 8.71 | 11.06 |  |  |  |
| Any | 2011-2019 | During Pregnancy | 24 | 12.11 | 10.81 | 13.41 |  |  |  |
| Any | 2011-2019 | During Pregnancy | 25 | 10.33 | 9.13 | 11.53 | 0.69 | 0.65 | 0.74 |
| Any | 2011-2019 | During Pregnancy | 26 | 10.99 | 9.75 | 12.23 |  |  |  |
| Any | 2011-2019 | During Pregnancy | 27 | 9.79 | 8.62 | 10.96 |  |  |  |
| Any | 2011-2019 | During Pregnancy | 28 | 11.11 | 9.86 | 12.36 |  |  |  |
| Any | 2011-2019 | During Pregnancy | 29 | 11.59 | 10.32 | 12.87 |  |  |  |
| Any | 2011-2019 | During Pregnancy | 30 | 10.00 | 8.81 | 11.18 | 0.63 | 0.58 | 0.67 |
| Any | 2011-2019 | During Pregnancy | 31 | 10.27 | 9.07 | 11.47 |  |  |  |
| Any | 2011-2019 | During Pregnancy | 32 | 10.14 | 8.94 | 11.33 |  |  |  |
| Any | 2011-2019 | During Pregnancy | 33 | 9.36 | 8.22 | 10.51 |  |  |  |
| Any | 2011-2019 | During Pregnancy | 34 | 7.46 | 6.43 | 8.48 |  |  |  |
| Any | 2011-2019 | During Pregnancy | 35 | 7.63 | 6.58 | 8.67 | 0.29 | 0.27 | 0.32 |
| Any | 2011-2019 | During Pregnancy | 36 | 5.82 | 4.90 | 6.74 |  |  |  |
| Any | 2011-2019 | During Pregnancy | 37 | 4.37 | 3.56 | 5.18 |  |  |  |
| Any | 2011-2019 | During Pregnancy | 38 | 2.29 | 1.67 | 2.92 |  |  |  |
| Any | 2011-2019 | During Pregnancy | 39 | 2.14 | 1.44 | 2.84 |  |  |  |
| Any | 2011-2019 | During Pregnancy | 40 | 1.63 | 0.80 | 2.45 |  |  |  |
| Any | 2011-2019 | During Pregnancy | 41 | 2.18 | 0.57 | 3.80 |  |  |  |
| Any | 2011-2019 | After Pregnancy | 0 | 6.34 | 5.41 | 7.27 | 0.66 | 0.61 | 0.71 |
| Any | 2011-2019 | After Pregnancy | 1 | 12.13 | 10.84 | 13.42 |  |  |  |
| Any | 2011-2019 | After Pregnancy | 2 | 10.41 | 9.21 | 11.60 |  |  |  |
| Any | 2011-2019 | After Pregnancy | 3 | 10.57 | 9.37 | 11.77 |  |  |  |
| Any | 2011-2019 | After Pregnancy | 4 | 12.26 | 10.97 | 13.56 |  |  |  |
| Any | 2011-2019 | After Pregnancy | 5 | 10.37 | 9.18 | 11.56 | 0.89 | 0.83 | 0.95 |
| Any | 2011-2019 | After Pregnancy | 6 | 14.75 | 13.33 | 16.18 |  |  |  |
| Any | 2011-2019 | After Pregnancy | 7 | 14.17 | 12.78 | 15.57 |  |  |  |
| Any | 2011-2019 | After Pregnancy | 8 | 16.45 | 14.94 | 17.95 |  |  |  |
| Any | 2011-2019 | After Pregnancy | 9 | 14.89 | 13.46 | 16.33 |  |  |  |
| Any | 2011-2019 | After Pregnancy | 10 | 14.15 | 12.75 | 15.56 | 0.89 | 0.84 | 0.95 |
| Any | 2011-2019 | After Pregnancy | 11 | 13.80 | 12.41 | 15.18 |  |  |  |
| Any | 2011-2019 | After Pregnancy | 12 | 13.07 | 11.72 | 14.43 |  |  |  |
| Any | 2011-2019 | After Pregnancy | 13 | 12.82 | 11.48 | 14.16 |  |  |  |
| Any | 2011-2019 | After Pregnancy | 14 | 12.21 | 10.90 | 13.52 |  |  |  |
| Any | 2011-2019 | After Pregnancy | 15 | 11.51 | 10.23 | 12.78 | 0.78 | 0.73 | 0.84 |
| Any | 2011-2019 | After Pregnancy | 16 | 11.75 | 10.46 | 13.04 |  |  |  |
| Any | 2011-2019 | After Pregnancy | 17 | 11.82 | 10.53 | 13.12 |  |  |  |
| Any | 2011-2019 | After Pregnancy | 18 | 13.03 | 11.66 | 14.40 |  |  |  |
| Any | 2011-2019 | After Pregnancy | 19 | 12.09 | 10.78 | 13.41 |  |  |  |
| Any | 2011-2019 | After Pregnancy | 20 | 10.44 | 9.22 | 11.67 | 0.73 | 0.68 | 0.79 |
| Any | 2011-2019 | After Pregnancy | 21 | 10.85 | 9.60 | 12.10 |  |  |  |
| Any | 2011-2019 | After Pregnancy | 22 | 11.43 | 10.14 | 12.71 |  |  |  |
| Any | 2011-2019 | After Pregnancy | 23 | 11.78 | 10.47 | 13.09 |  |  |  |
| Any | 2011-2019 | After Pregnancy | 24 | 11.56 | 10.26 | 12.86 |  |  |  |
| Any | 2011-2019 | After Pregnancy | 25 | 11.23 | 9.95 | 12.51 | 0.75 | 0.70 | 0.80 |
| Any | 2011-2019 | After Pregnancy | 26 | 12.01 | 10.68 | 13.34 |  |  |  |
| Any | 2011-2019 | After Pregnancy | 27 | 11.80 | 10.48 | 13.12 |  |  |  |
| Any | 2011-2019 | After Pregnancy | 28 | 10.13 | 8.91 | 11.36 |  |  |  |
| Any | 2011-2019 | After Pregnancy | 29 | 10.96 | 9.68 | 12.24 |  |  |  |
| Any | 2011-2019 | After Pregnancy | 30 | 9.72 | 8.51 | 10.93 | 0.70 | 0.65 | 0.75 |
| Any | 2011-2019 | After Pregnancy | 31 | 9.44 | 8.25 | 10.64 |  |  |  |
| Any | 2011-2019 | After Pregnancy | 32 | 11.67 | 10.34 | 13.00 |  |  |  |
| Any | 2011-2019 | After Pregnancy | 33 | 9.28 | 8.09 | 10.47 |  |  |  |
| Any | 2011-2019 | After Pregnancy | 34 | 11.09 | 9.79 | 12.39 |  |  |  |
| Any | 2011-2019 | After Pregnancy | 35 | 10.54 | 9.27 | 11.82 | 0.70 | 0.66 | 0.76 |
| Any | 2011-2019 | After Pregnancy | 36 | 11.03 | 9.72 | 12.33 |  |  |  |
| Any | 2011-2019 | After Pregnancy | 37 | 11.09 | 9.77 | 12.40 |  |  |  |
| Any | 2011-2019 | After Pregnancy | 38 | 11.23 | 9.91 | 12.56 |  |  |  |
| Any | 2011-2019 | After Pregnancy | 39 | 11.28 | 9.95 | 12.61 |  |  |  |
| Any | 2011-2019 | After Pregnancy | 40 | 11.03 | 9.71 | 12.34 | 0.73 | 0.68 | 0.78 |
| Any | 2011-2019 | After Pregnancy | 41 | 9.89 | 8.64 | 11.14 |  |  |  |
| Any | 2011-2019 | After Pregnancy | 42 | 10.69 | 9.38 | 12.00 |  |  |  |
| Any | 2011-2019 | After Pregnancy | 43 | 11.70 | 10.33 | 13.07 |  |  |  |
| Any | 2011-2019 | After Pregnancy | 44 | 9.71 | 8.46 | 10.96 |  |  |  |
| Any | 2011-2019 | After Pregnancy | 45 | 11.44 | 10.07 | 12.80 | 0.78 | 0.73 | 0.84 |
| Any | 2011-2019 | After Pregnancy | 46 | 10.98 | 9.65 | 12.32 |  |  |  |
| Any | 2011-2019 | After Pregnancy | 47 | 12.06 | 10.65 | 13.46 |  |  |  |
| Any | 2011-2019 | After Pregnancy | 48 | 11.74 | 10.35 | 13.14 |  |  |  |
| Any | 2011-2019 | After Pregnancy | 49 | 12.10 | 10.68 | 13.52 |  |  |  |
| Any | 2011-2019 | After Pregnancy | 50 | 10.12 | 8.81 | 11.42 | 0.78 | 0.69 | 0.87 |
| Any | 2011-2019 | After Pregnancy | 51 | 11.32 | 10.03 | 12.62 |  |  |  |
| Depression | 2003-2010 | Before Pregnancy | 0 | 5.03 | 4.13 | 5.93 | Ref. | Ref. | Ref. |
| Depression | 2003-2010 | Before Pregnancy | 1 | 3.88 | 3.09 | 4.66 |  |  |  |
| Depression | 2003-2010 | Before Pregnancy | 2 | 3.65 | 2.88 | 4.42 |  |  |  |
| Depression | 2003-2010 | Before Pregnancy | 3 | 4.58 | 3.73 | 5.44 |  |  |  |
| Depression | 2003-2010 | Before Pregnancy | 4 | 5.23 | 4.32 | 6.14 |  |  |  |
| Depression | 2003-2010 | Before Pregnancy | 5 | 4.60 | 3.75 | 5.45 | Ref. | Ref. | Ref. |
| Depression | 2003-2010 | Before Pregnancy | 6 | 4.83 | 3.96 | 5.71 |  |  |  |
| Depression | 2003-2010 | Before Pregnancy | 7 | 4.47 | 3.63 | 5.31 |  |  |  |
| Depression | 2003-2010 | Before Pregnancy | 8 | 4.42 | 3.58 | 5.25 |  |  |  |
| Depression | 2003-2010 | Before Pregnancy | 9 | 4.77 | 3.91 | 5.64 |  |  |  |
| Depression | 2003-2010 | Before Pregnancy | 10 | 4.38 | 3.55 | 5.20 | Ref. | Ref. | Ref. |
| Depression | 2003-2010 | Before Pregnancy | 11 | 4.42 | 3.59 | 5.25 |  |  |  |
| Depression | 2003-2010 | Before Pregnancy | 12 | 3.50 | 2.77 | 4.24 |  |  |  |
| Depression | 2003-2010 | Before Pregnancy | 13 | 4.86 | 4.00 | 5.73 |  |  |  |
| Depression | 2003-2010 | Before Pregnancy | 14 | 4.25 | 3.44 | 5.06 |  |  |  |
| Depression | 2003-2010 | Before Pregnancy | 15 | 4.47 | 3.65 | 5.30 | Ref. | Ref. | Ref. |
| Depression | 2003-2010 | Before Pregnancy | 16 | 4.26 | 3.45 | 5.06 |  |  |  |
| Depression | 2003-2010 | Before Pregnancy | 17 | 4.45 | 3.62 | 5.27 |  |  |  |
| Depression | 2003-2010 | Before Pregnancy | 18 | 4.23 | 3.43 | 5.03 |  |  |  |
| Depression | 2003-2010 | Before Pregnancy | 19 | 4.10 | 3.31 | 4.89 |  |  |  |
| Depression | 2003-2010 | Before Pregnancy | 20 | 4.09 | 3.30 | 4.87 | Ref. | Ref. | Ref. |
| Depression | 2003-2010 | Before Pregnancy | 21 | 4.82 | 3.96 | 5.67 |  |  |  |
| Depression | 2003-2010 | Before Pregnancy | 22 | 4.87 | 4.02 | 5.73 |  |  |  |
| Depression | 2003-2010 | Before Pregnancy | 23 | 4.39 | 3.58 | 5.20 |  |  |  |
| Depression | 2003-2010 | Before Pregnancy | 24 | 3.99 | 3.22 | 4.76 |  |  |  |
| Depression | 2003-2010 | Before Pregnancy | 25 | 4.44 | 3.63 | 5.25 | Ref. | Ref. | Ref. |
| Depression | 2003-2010 | Before Pregnancy | 26 | 4.39 | 3.58 | 5.19 |  |  |  |
| Depression | 2003-2010 | Before Pregnancy | 27 | 4.52 | 3.71 | 5.34 |  |  |  |
| Depression | 2003-2010 | Before Pregnancy | 28 | 5.16 | 4.29 | 6.03 |  |  |  |
| Depression | 2003-2010 | Before Pregnancy | 29 | 5.06 | 4.20 | 5.92 |  |  |  |
| Depression | 2003-2010 | Before Pregnancy | 30 | 4.55 | 3.74 | 5.37 | Ref. | Ref. | Ref. |
| Depression | 2003-2010 | Before Pregnancy | 31 | 4.38 | 3.59 | 5.18 |  |  |  |
| Depression | 2003-2010 | Before Pregnancy | 32 | 4.44 | 3.64 | 5.25 |  |  |  |
| Depression | 2003-2010 | Before Pregnancy | 33 | 4.39 | 3.60 | 5.19 |  |  |  |
| Depression | 2003-2010 | Before Pregnancy | 34 | 4.74 | 3.92 | 5.57 |  |  |  |
| Depression | 2003-2010 | Before Pregnancy | 35 | 4.02 | 3.26 | 4.78 | Ref. | Ref. | Ref. |
| Depression | 2003-2010 | Before Pregnancy | 36 | 4.71 | 3.89 | 5.53 |  |  |  |
| Depression | 2003-2010 | Before Pregnancy | 37 | 3.69 | 2.97 | 4.42 |  |  |  |
| Depression | 2003-2010 | Before Pregnancy | 38 | 3.96 | 3.21 | 4.71 |  |  |  |
| Depression | 2003-2010 | Before Pregnancy | 39 | 3.99 | 3.24 | 4.74 |  |  |  |
| Depression | 2003-2010 | Before Pregnancy | 40 | 3.69 | 2.97 | 4.40 | Ref. | Ref. | Ref. |
| Depression | 2003-2010 | Before Pregnancy | 41 | 3.94 | 3.19 | 4.68 |  |  |  |
| Depression | 2003-2010 | Before Pregnancy | 42 | 4.06 | 3.31 | 4.82 |  |  |  |
| Depression | 2003-2010 | Before Pregnancy | 43 | 3.98 | 3.23 | 4.72 |  |  |  |
| Depression | 2003-2010 | Before Pregnancy | 44 | 4.47 | 3.69 | 5.26 |  |  |  |
| Depression | 2003-2010 | Before Pregnancy | 45 | 4.06 | 3.31 | 4.80 | Ref. | Ref. | Ref. |
| Depression | 2003-2010 | Before Pregnancy | 46 | 3.76 | 3.04 | 4.48 |  |  |  |
| Depression | 2003-2010 | Before Pregnancy | 47 | 4.64 | 3.84 | 5.43 |  |  |  |
| Depression | 2003-2010 | Before Pregnancy | 48 | 4.23 | 3.47 | 4.99 |  |  |  |
| Depression | 2003-2010 | Before Pregnancy | 49 | 3.91 | 3.18 | 4.64 |  |  |  |
| Depression | 2003-2010 | Before Pregnancy | 50 | 4.83 | 4.02 | 5.63 | Ref. | Ref. | Ref. |
| Depression | 2003-2010 | Before Pregnancy | 51 | 4.23 | 3.47 | 4.98 |  |  |  |
| Depression | 2003-2010 | During Pregnancy | 0 | 4.43 | 3.69 | 5.17 | 0.88 | 0.78 | 0.99 |
| Depression | 2003-2010 | During Pregnancy | 1 | 3.65 | 2.98 | 4.31 |  |  |  |
| Depression | 2003-2010 | During Pregnancy | 2 | 3.81 | 3.12 | 4.49 |  |  |  |
| Depression | 2003-2010 | During Pregnancy | 3 | 3.99 | 3.29 | 4.69 |  |  |  |
| Depression | 2003-2010 | During Pregnancy | 4 | 2.78 | 2.20 | 3.36 |  |  |  |
| Depression | 2003-2010 | During Pregnancy | 5 | 2.55 | 1.99 | 3.11 | 0.56 | 0.49 | 0.64 |
| Depression | 2003-2010 | During Pregnancy | 6 | 3.13 | 2.51 | 3.75 |  |  |  |
| Depression | 2003-2010 | During Pregnancy | 7 | 2.20 | 1.68 | 2.72 |  |  |  |
| Depression | 2003-2010 | During Pregnancy | 8 | 2.11 | 1.60 | 2.61 |  |  |  |
| Depression | 2003-2010 | During Pregnancy | 9 | 2.33 | 1.79 | 2.86 |  |  |  |
| Depression | 2003-2010 | During Pregnancy | 10 | 2.77 | 2.19 | 3.35 | 0.61 | 0.53 | 0.69 |
| Depression | 2003-2010 | During Pregnancy | 11 | 2.17 | 1.65 | 2.68 |  |  |  |
| Depression | 2003-2010 | During Pregnancy | 12 | 1.91 | 1.43 | 2.40 |  |  |  |
| Depression | 2003-2010 | During Pregnancy | 13 | 2.90 | 2.30 | 3.49 |  |  |  |
| Depression | 2003-2010 | During Pregnancy | 14 | 2.80 | 2.22 | 3.39 |  |  |  |
| Depression | 2003-2010 | During Pregnancy | 15 | 2.42 | 1.88 | 2.96 | 0.62 | 0.54 | 0.70 |
| Depression | 2003-2010 | During Pregnancy | 16 | 2.45 | 1.90 | 3.00 |  |  |  |
| Depression | 2003-2010 | During Pregnancy | 17 | 2.74 | 2.16 | 3.31 |  |  |  |
| Depression | 2003-2010 | During Pregnancy | 18 | 2.48 | 1.93 | 3.03 |  |  |  |
| Depression | 2003-2010 | During Pregnancy | 19 | 2.70 | 2.13 | 3.28 |  |  |  |
| Depression | 2003-2010 | During Pregnancy | 20 | 2.76 | 2.18 | 3.35 | 0.60 | 0.53 | 0.69 |
| Depression | 2003-2010 | During Pregnancy | 21 | 2.64 | 2.07 | 3.20 |  |  |  |
| Depression | 2003-2010 | During Pregnancy | 22 | 2.57 | 2.01 | 3.13 |  |  |  |
| Depression | 2003-2010 | During Pregnancy | 23 | 2.57 | 2.01 | 3.13 |  |  |  |
| Depression | 2003-2010 | During Pregnancy | 24 | 2.41 | 1.87 | 2.96 |  |  |  |
| Depression | 2003-2010 | During Pregnancy | 25 | 2.79 | 2.21 | 3.38 | 0.50 | 0.44 | 0.57 |
| Depression | 2003-2010 | During Pregnancy | 26 | 2.38 | 1.84 | 2.92 |  |  |  |
| Depression | 2003-2010 | During Pregnancy | 27 | 2.16 | 1.65 | 2.67 |  |  |  |
| Depression | 2003-2010 | During Pregnancy | 28 | 1.78 | 1.31 | 2.25 |  |  |  |
| Depression | 2003-2010 | During Pregnancy | 29 | 2.35 | 1.82 | 2.89 |  |  |  |
| Depression | 2003-2010 | During Pregnancy | 30 | 2.07 | 1.57 | 2.57 | 0.37 | 0.32 | 0.43 |
| Depression | 2003-2010 | During Pregnancy | 31 | 1.37 | 0.96 | 1.78 |  |  |  |
| Depression | 2003-2010 | During Pregnancy | 32 | 1.60 | 1.15 | 2.04 |  |  |  |
| Depression | 2003-2010 | During Pregnancy | 33 | 1.67 | 1.22 | 2.12 |  |  |  |
| Depression | 2003-2010 | During Pregnancy | 34 | 1.36 | 0.95 | 1.77 |  |  |  |
| Depression | 2003-2010 | During Pregnancy | 35 | 1.34 | 0.93 | 1.74 | 0.23 | 0.19 | 0.28 |
| Depression | 2003-2010 | During Pregnancy | 36 | 0.93 | 0.58 | 1.27 |  |  |  |
| Depression | 2003-2010 | During Pregnancy | 37 | 0.76 | 0.44 | 1.08 |  |  |  |
| Depression | 2003-2010 | During Pregnancy | 38 | 0.83 | 0.47 | 1.18 |  |  |  |
| Depression | 2003-2010 | During Pregnancy | 39 | 0.53 | 0.20 | 0.86 |  |  |  |
| Depression | 2003-2010 | During Pregnancy | 40 | 0.29 | 0.00 | 0.62 | - | - | |
| Depression | 2003-2010 | During Pregnancy | 41 | 0.29 | 0.00 | 0.86 |  |  |  |
| Depression | 2003-2010 | After Pregnancy | 0 | 1.85 | 1.37 | 2.33 | 0.84 | 0.75 | 0.95 |
| Depression | 2003-2010 | After Pregnancy | 1 | 3.09 | 2.47 | 3.71 |  |  |  |
| Depression | 2003-2010 | After Pregnancy | 2 | 4.16 | 3.44 | 4.89 |  |  |  |
| Depression | 2003-2010 | After Pregnancy | 3 | 4.59 | 3.83 | 5.35 |  |  |  |
| Depression | 2003-2010 | After Pregnancy | 4 | 4.43 | 3.69 | 5.18 |  |  |  |
| Depression | 2003-2010 | After Pregnancy | 5 | 4.16 | 3.44 | 4.89 | 1.02 | 0.92 | 1.14 |
| Depression | 2003-2010 | After Pregnancy | 6 | 4.76 | 3.99 | 5.54 |  |  |  |
| Depression | 2003-2010 | After Pregnancy | 7 | 5.09 | 4.29 | 5.88 |  |  |  |
| Depression | 2003-2010 | After Pregnancy | 8 | 4.54 | 3.78 | 5.29 |  |  |  |
| Depression | 2003-2010 | After Pregnancy | 9 | 4.50 | 3.75 | 5.25 |  |  |  |
| Depression | 2003-2010 | After Pregnancy | 10 | 5.12 | 4.32 | 5.92 | 1.25 | 1.12 | 1.39 |
| Depression | 2003-2010 | After Pregnancy | 11 | 4.93 | 4.15 | 5.72 |  |  |  |
| Depression | 2003-2010 | After Pregnancy | 12 | 5.66 | 4.81 | 6.50 |  |  |  |
| Depression | 2003-2010 | After Pregnancy | 13 | 5.36 | 4.54 | 6.18 |  |  |  |
| Depression | 2003-2010 | After Pregnancy | 14 | 5.12 | 4.31 | 5.92 |  |  |  |
| Depression | 2003-2010 | After Pregnancy | 15 | 4.86 | 4.08 | 5.64 | 1.08 | 0.97 | 1.21 |
| Depression | 2003-2010 | After Pregnancy | 16 | 4.47 | 3.72 | 5.22 |  |  |  |
| Depression | 2003-2010 | After Pregnancy | 17 | 4.64 | 3.87 | 5.40 |  |  |  |
| Depression | 2003-2010 | After Pregnancy | 18 | 4.51 | 3.76 | 5.27 |  |  |  |
| Depression | 2003-2010 | After Pregnancy | 19 | 4.42 | 3.67 | 5.16 |  |  |  |
| Depression | 2003-2010 | After Pregnancy | 20 | 4.13 | 3.40 | 4.85 | 0.94 | 0.84 | 1.05 |
| Depression | 2003-2010 | After Pregnancy | 21 | 4.30 | 3.56 | 5.04 |  |  |  |
| Depression | 2003-2010 | After Pregnancy | 22 | 4.27 | 3.53 | 5.01 |  |  |  |
| Depression | 2003-2010 | After Pregnancy | 23 | 3.35 | 2.70 | 4.00 |  |  |  |
| Depression | 2003-2010 | After Pregnancy | 24 | 4.55 | 3.79 | 5.31 |  |  |  |
| Depression | 2003-2010 | After Pregnancy | 25 | 4.19 | 3.46 | 4.92 | 0.92 | 0.82 | 1.03 |
| Depression | 2003-2010 | After Pregnancy | 26 | 4.06 | 3.34 | 4.78 |  |  |  |
| Depression | 2003-2010 | After Pregnancy | 27 | 4.30 | 3.56 | 5.05 |  |  |  |
| Depression | 2003-2010 | After Pregnancy | 28 | 4.75 | 3.97 | 5.53 |  |  |  |
| Depression | 2003-2010 | After Pregnancy | 29 | 4.25 | 3.51 | 4.99 |  |  |  |
| Depression | 2003-2010 | After Pregnancy | 30 | 3.96 | 3.25 | 4.68 | 1.00 | 0.89 | 1.11 |
| Depression | 2003-2010 | After Pregnancy | 31 | 4.71 | 3.93 | 5.49 |  |  |  |
| Depression | 2003-2010 | After Pregnancy | 32 | 4.38 | 3.63 | 5.14 |  |  |  |
| Depression | 2003-2010 | After Pregnancy | 33 | 4.90 | 4.10 | 5.70 |  |  |  |
| Depression | 2003-2010 | After Pregnancy | 34 | 4.33 | 3.58 | 5.09 |  |  |  |
| Depression | 2003-2010 | After Pregnancy | 35 | 4.31 | 3.56 | 5.06 | 1.10 | 0.99 | 1.24 |
| Depression | 2003-2010 | After Pregnancy | 36 | 4.29 | 3.54 | 5.04 |  |  |  |
| Depression | 2003-2010 | After Pregnancy | 37 | 4.95 | 4.14 | 5.75 |  |  |  |
| Depression | 2003-2010 | After Pregnancy | 38 | 4.45 | 3.68 | 5.21 |  |  |  |
| Depression | 2003-2010 | After Pregnancy | 39 | 4.46 | 3.69 | 5.23 |  |  |  |
| Depression | 2003-2010 | After Pregnancy | 40 | 4.47 | 3.70 | 5.24 | 1.13 | 1.01 | 1.26 |
| Depression | 2003-2010 | After Pregnancy | 41 | 4.77 | 3.97 | 5.56 |  |  |  |
| Depression | 2003-2010 | After Pregnancy | 42 | 4.57 | 3.79 | 5.36 |  |  |  |
| Depression | 2003-2010 | After Pregnancy | 43 | 4.62 | 3.83 | 5.41 |  |  |  |
| Depression | 2003-2010 | After Pregnancy | 44 | 4.22 | 3.46 | 4.97 |  |  |  |
| Depression | 2003-2010 | After Pregnancy | 45 | 4.05 | 3.32 | 4.79 | 1.10 | 0.98 | 1.23 |
| Depression | 2003-2010 | After Pregnancy | 46 | 4.61 | 3.82 | 5.40 |  |  |  |
| Depression | 2003-2010 | After Pregnancy | 47 | 4.72 | 3.92 | 5.52 |  |  |  |
| Depression | 2003-2010 | After Pregnancy | 48 | 4.53 | 3.74 | 5.31 |  |  |  |
| Depression | 2003-2010 | After Pregnancy | 49 | 4.66 | 3.86 | 5.45 |  |  |  |
| Depression | 2003-2010 | After Pregnancy | 50 | 4.56 | 3.77 | 5.35 | 1.03 | 0.87 | 1.22 |
| Depression | 2003-2010 | After Pregnancy | 51 | 4.65 | 3.91 | 5.40 |  |  |  |
| Depression | 2011-2019 | Before Pregnancy | 0 | 8.49 | 7.31 | 9.68 | Ref. | Ref. | Ref. |
| Depression | 2011-2019 | Before Pregnancy | 1 | 6.67 | 5.62 | 7.71 |  |  |  |
| Depression | 2011-2019 | Before Pregnancy | 2 | 7.31 | 6.22 | 8.40 |  |  |  |
| Depression | 2011-2019 | Before Pregnancy | 3 | 8.11 | 6.96 | 9.26 |  |  |  |
| Depression | 2011-2019 | Before Pregnancy | 4 | 7.86 | 6.73 | 8.99 |  |  |  |
| Depression | 2011-2019 | Before Pregnancy | 5 | 6.93 | 5.87 | 7.98 | Ref. | Ref. | Ref. |
| Depression | 2011-2019 | Before Pregnancy | 6 | 7.32 | 6.23 | 8.40 |  |  |  |
| Depression | 2011-2019 | Before Pregnancy | 7 | 7.46 | 6.37 | 8.55 |  |  |  |
| Depression | 2011-2019 | Before Pregnancy | 8 | 7.27 | 6.19 | 8.35 |  |  |  |
| Depression | 2011-2019 | Before Pregnancy | 9 | 7.86 | 6.74 | 8.97 |  |  |  |
| Depression | 2011-2019 | Before Pregnancy | 10 | 7.74 | 6.63 | 8.84 | Ref. | Ref. | Ref. |
| Depression | 2011-2019 | Before Pregnancy | 11 | 6.61 | 5.59 | 7.63 |  |  |  |
| Depression | 2011-2019 | Before Pregnancy | 12 | 5.88 | 4.92 | 6.85 |  |  |  |
| Depression | 2011-2019 | Before Pregnancy | 13 | 7.03 | 5.99 | 8.08 |  |  |  |
| Depression | 2011-2019 | Before Pregnancy | 14 | 7.49 | 6.41 | 8.57 |  |  |  |
| Depression | 2011-2019 | Before Pregnancy | 15 | 7.10 | 6.05 | 8.15 | Ref. | Ref. | Ref. |
| Depression | 2011-2019 | Before Pregnancy | 16 | 6.34 | 5.36 | 7.33 |  |  |  |
| Depression | 2011-2019 | Before Pregnancy | 17 | 6.88 | 5.85 | 7.91 |  |  |  |
| Depression | 2011-2019 | Before Pregnancy | 18 | 7.80 | 6.71 | 8.89 |  |  |  |
| Depression | 2011-2019 | Before Pregnancy | 19 | 7.21 | 6.17 | 8.26 |  |  |  |
| Depression | 2011-2019 | Before Pregnancy | 20 | 6.83 | 5.81 | 7.85 | Ref. | Ref. | Ref. |
| Depression | 2011-2019 | Before Pregnancy | 21 | 6.28 | 5.31 | 7.26 |  |  |  |
| Depression | 2011-2019 | Before Pregnancy | 22 | 7.23 | 6.19 | 8.28 |  |  |  |
| Depression | 2011-2019 | Before Pregnancy | 23 | 6.31 | 5.34 | 7.28 |  |  |  |
| Depression | 2011-2019 | Before Pregnancy | 24 | 7.06 | 6.03 | 8.08 |  |  |  |
| Depression | 2011-2019 | Before Pregnancy | 25 | 6.87 | 5.86 | 7.88 | Ref. | Ref. | Ref. |
| Depression | 2011-2019 | Before Pregnancy | 26 | 6.88 | 5.87 | 7.88 |  |  |  |
| Depression | 2011-2019 | Before Pregnancy | 27 | 6.81 | 5.81 | 7.81 |  |  |  |
| Depression | 2011-2019 | Before Pregnancy | 28 | 6.97 | 5.96 | 7.98 |  |  |  |
| Depression | 2011-2019 | Before Pregnancy | 29 | 7.17 | 6.14 | 8.19 |  |  |  |
| Depression | 2011-2019 | Before Pregnancy | 30 | 7.32 | 6.29 | 8.35 | Ref. | Ref. | Ref. |
| Depression | 2011-2019 | Before Pregnancy | 31 | 6.77 | 5.78 | 7.75 |  |  |  |
| Depression | 2011-2019 | Before Pregnancy | 32 | 7.29 | 6.26 | 8.31 |  |  |  |
| Depression | 2011-2019 | Before Pregnancy | 33 | 6.74 | 5.76 | 7.72 |  |  |  |
| Depression | 2011-2019 | Before Pregnancy | 34 | 6.45 | 5.49 | 7.41 |  |  |  |
| Depression | 2011-2019 | Before Pregnancy | 35 | 6.12 | 5.19 | 7.06 | Ref. | Ref. | Ref. |
| Depression | 2011-2019 | Before Pregnancy | 36 | 7.17 | 6.16 | 8.18 |  |  |  |
| Depression | 2011-2019 | Before Pregnancy | 37 | 7.49 | 6.46 | 8.51 |  |  |  |
| Depression | 2011-2019 | Before Pregnancy | 38 | 6.18 | 5.25 | 7.11 |  |  |  |
| Depression | 2011-2019 | Before Pregnancy | 39 | 7.14 | 6.14 | 8.13 |  |  |  |
| Depression | 2011-2019 | Before Pregnancy | 40 | 5.99 | 5.08 | 6.91 | Ref. | Ref. | Ref. |
| Depression | 2011-2019 | Before Pregnancy | 41 | 6.57 | 5.62 | 7.52 |  |  |  |
| Depression | 2011-2019 | Before Pregnancy | 42 | 6.66 | 5.70 | 7.62 |  |  |  |
| Depression | 2011-2019 | Before Pregnancy | 43 | 7.05 | 6.07 | 8.04 |  |  |  |
| Depression | 2011-2019 | Before Pregnancy | 44 | 6.05 | 5.14 | 6.96 |  |  |  |
| Depression | 2011-2019 | Before Pregnancy | 45 | 6.49 | 5.55 | 7.43 | Ref. | Ref. | Ref. |
| Depression | 2011-2019 | Before Pregnancy | 46 | 5.42 | 4.56 | 6.27 |  |  |  |
| Depression | 2011-2019 | Before Pregnancy | 47 | 5.80 | 4.92 | 6.69 |  |  |  |
| Depression | 2011-2019 | Before Pregnancy | 48 | 5.95 | 5.06 | 6.85 |  |  |  |
| Depression | 2011-2019 | Before Pregnancy | 49 | 7.27 | 6.29 | 8.26 |  |  |  |
| Depression | 2011-2019 | Before Pregnancy | 50 | 5.89 | 5.01 | 6.77 |  |  |  |
| Depression | 2011-2019 | Before Pregnancy | 51 | 5.76 | 4.89 | 6.63 | Ref | Ref. | Ref. |
| Depression | 2011-2019 | During Pregnancy | 0 | 6.62 | 5.71 | 7.52 | 0.73 | 0.67 | 0.80 |
| Depression | 2011-2019 | During Pregnancy | 1 | 5.42 | 4.61 | 6.24 |  |  |  |
| Depression | 2011-2019 | During Pregnancy | 2 | 6.28 | 5.40 | 7.15 |  |  |  |
| Depression | 2011-2019 | During Pregnancy | 3 | 4.83 | 4.06 | 5.61 |  |  |  |
| Depression | 2011-2019 | During Pregnancy | 4 | 4.39 | 3.65 | 5.12 |  |  |  |
| Depression | 2011-2019 | During Pregnancy | 5 | 4.44 | 3.71 | 5.18 | 0.59 | 0.54 | 0.66 |
| Depression | 2011-2019 | During Pregnancy | 6 | 4.25 | 3.53 | 4.97 |  |  |  |
| Depression | 2011-2019 | During Pregnancy | 7 | 4.67 | 3.91 | 5.42 |  |  |  |
| Depression | 2011-2019 | During Pregnancy | 8 | 4.06 | 3.35 | 4.76 |  |  |  |
| Depression | 2011-2019 | During Pregnancy | 9 | 4.25 | 3.53 | 4.97 |  |  |  |
| Depression | 2011-2019 | During Pregnancy | 10 | 5.01 | 4.23 | 5.80 | 0.74 | 0.67 | 0.82 |
| Depression | 2011-2019 | During Pregnancy | 11 | 5.01 | 4.23 | 5.79 |  |  |  |
| Depression | 2011-2019 | During Pregnancy | 12 | 5.17 | 4.37 | 5.96 |  |  |  |
| Depression | 2011-2019 | During Pregnancy | 13 | 4.59 | 3.84 | 5.34 |  |  |  |
| Depression | 2011-2019 | During Pregnancy | 14 | 6.02 | 5.17 | 6.88 |  |  |  |
| Depression | 2011-2019 | During Pregnancy | 15 | 5.29 | 4.49 | 6.09 | 0.72 | 0.66 | 0.80 |
| Depression | 2011-2019 | During Pregnancy | 16 | 5.26 | 4.45 | 6.06 |  |  |  |
| Depression | 2011-2019 | During Pregnancy | 17 | 4.97 | 4.19 | 5.75 |  |  |  |
| Depression | 2011-2019 | During Pregnancy | 18 | 5.67 | 4.83 | 6.50 |  |  |  |
| Depression | 2011-2019 | During Pregnancy | 19 | 4.65 | 3.89 | 5.40 |  |  |  |
| Depression | 2011-2019 | During Pregnancy | 20 | 4.68 | 3.92 | 5.43 | 0.73 | 0.66 | 0.81 |
| Depression | 2011-2019 | During Pregnancy | 21 | 4.83 | 4.06 | 5.60 |  |  |  |
| Depression | 2011-2019 | During Pregnancy | 22 | 5.62 | 4.80 | 6.45 |  |  |  |
| Depression | 2011-2019 | During Pregnancy | 23 | 4.45 | 3.71 | 5.19 |  |  |  |
| Depression | 2011-2019 | During Pregnancy | 24 | 5.28 | 4.47 | 6.08 |  |  |  |
| Depression | 2011-2019 | During Pregnancy | 25 | 5.47 | 4.65 | 6.29 | 0.70 | 0.64 | 0.77 |
| Depression | 2011-2019 | During Pregnancy | 26 | 4.77 | 4.01 | 5.54 |  |  |  |
| Depression | 2011-2019 | During Pregnancy | 27 | 4.68 | 3.92 | 5.43 |  |  |  |
| Depression | 2011-2019 | During Pregnancy | 28 | 4.81 | 4.04 | 5.57 |  |  |  |
| Depression | 2011-2019 | During Pregnancy | 29 | 4.90 | 4.13 | 5.68 |  |  |  |
| Depression | 2011-2019 | During Pregnancy | 30 | 5.16 | 4.37 | 5.96 | 0.61 | 0.55 | 0.67 |
| Depression | 2011-2019 | During Pregnancy | 31 | 4.66 | 3.90 | 5.41 |  |  |  |
| Depression | 2011-2019 | During Pregnancy | 32 | 4.16 | 3.44 | 4.87 |  |  |  |
| Depression | 2011-2019 | During Pregnancy | 33 | 3.81 | 3.13 | 4.50 |  |  |  |
| Depression | 2011-2019 | During Pregnancy | 34 | 3.32 | 2.68 | 3.96 |  |  |  |
| Depression | 2011-2019 | During Pregnancy | 35 | 3.12 | 2.50 | 3.74 | 0.28 | 0.25 | 0.33 |
| Depression | 2011-2019 | During Pregnancy | 36 | 2.31 | 1.77 | 2.85 |  |  |  |
| Depression | 2011-2019 | During Pregnancy | 37 | 1.55 | 1.10 | 2.00 |  |  |  |
| Depression | 2011-2019 | During Pregnancy | 38 | 1.32 | 0.87 | 1.76 |  |  |  |
| Depression | 2011-2019 | During Pregnancy | 39 | 1.00 | 0.55 | 1.45 |  |  |  |
| Depression | 2011-2019 | During Pregnancy | 40 | 0.58 | 0.12 | 1.04 | - | - | |
| Depression | 2011-2019 | During Pregnancy | 41 | 0.56 | 0.00 | 1.33 |  |  |  |
| Depression | 2011-2019 | After Pregnancy | 0 | 2.71 | 2.14 | 3.28 | 0.87 | 0.80 | 0.96 |
| Depression | 2011-2019 | After Pregnancy | 1 | 6.76 | 5.86 | 7.65 |  |  |  |
| Depression | 2011-2019 | After Pregnancy | 2 | 6.68 | 5.79 | 7.58 |  |  |  |
| Depression | 2011-2019 | After Pregnancy | 3 | 7.37 | 6.43 | 8.30 |  |  |  |
| Depression | 2011-2019 | After Pregnancy | 4 | 8.12 | 7.14 | 9.11 |  |  |  |
| Depression | 2011-2019 | After Pregnancy | 5 | 7.71 | 6.75 | 8.67 | 1.25 | 1.15 | 1.37 |
| Depression | 2011-2019 | After Pregnancy | 6 | 8.55 | 7.54 | 9.56 |  |  |  |
| Depression | 2011-2019 | After Pregnancy | 7 | 9.09 | 8.05 | 10.14 |  |  |  |
| Depression | 2011-2019 | After Pregnancy | 8 | 10.48 | 9.36 | 11.60 |  |  |  |
| Depression | 2011-2019 | After Pregnancy | 9 | 8.20 | 7.21 | 9.20 |  |  |  |
| Depression | 2011-2019 | After Pregnancy | 10 | 8.39 | 7.38 | 9.40 | 1.21 | 1.11 | 1.32 |
| Depression | 2011-2019 | After Pregnancy | 11 | 8.74 | 7.71 | 9.77 |  |  |  |
| Depression | 2011-2019 | After Pregnancy | 12 | 8.04 | 7.05 | 9.03 |  |  |  |
| Depression | 2011-2019 | After Pregnancy | 13 | 8.22 | 7.22 | 9.22 |  |  |  |
| Depression | 2011-2019 | After Pregnancy | 14 | 6.87 | 5.95 | 7.78 |  |  |  |
| Depression | 2011-2019 | After Pregnancy | 15 | 7.35 | 6.40 | 8.31 | 1.01 | 0.92 | 1.10 |
| Depression | 2011-2019 | After Pregnancy | 16 | 6.74 | 5.82 | 7.65 |  |  |  |
| Depression | 2011-2019 | After Pregnancy | 17 | 7.42 | 6.46 | 8.38 |  |  |  |
| Depression | 2011-2019 | After Pregnancy | 18 | 7.01 | 6.08 | 7.94 |  |  |  |
| Depression | 2011-2019 | After Pregnancy | 19 | 5.95 | 5.09 | 6.81 |  |  |  |
| Depression | 2011-2019 | After Pregnancy | 20 | 5.61 | 4.77 | 6.45 | 0.90 | 0.82 | 0.98 |
| Depression | 2011-2019 | After Pregnancy | 21 | 5.46 | 4.63 | 6.29 |  |  |  |
| Depression | 2011-2019 | After Pregnancy | 22 | 6.08 | 5.21 | 6.96 |  |  |  |
| Depression | 2011-2019 | After Pregnancy | 23 | 5.97 | 5.10 | 6.84 |  |  |  |
| Depression | 2011-2019 | After Pregnancy | 24 | 6.36 | 5.46 | 7.26 |  |  |  |
| Depression | 2011-2019 | After Pregnancy | 25 | 5.69 | 4.83 | 6.54 | 0.88 | 0.80 | 0.97 |
| Depression | 2011-2019 | After Pregnancy | 26 | 5.91 | 5.04 | 6.78 |  |  |  |
| Depression | 2011-2019 | After Pregnancy | 27 | 6.11 | 5.22 | 6.99 |  |  |  |
| Depression | 2011-2019 | After Pregnancy | 28 | 6.25 | 5.36 | 7.15 |  |  |  |
| Depression | 2011-2019 | After Pregnancy | 29 | 6.12 | 5.23 | 7.01 |  |  |  |
| Depression | 2011-2019 | After Pregnancy | 30 | 5.81 | 4.94 | 6.68 | 0.88 | 0.80 | 0.96 |
| Depression | 2011-2019 | After Pregnancy | 31 | 6.03 | 5.14 | 6.92 |  |  |  |
| Depression | 2011-2019 | After Pregnancy | 32 | 6.06 | 5.17 | 6.95 |  |  |  |
| Depression | 2011-2019 | After Pregnancy | 33 | 5.50 | 4.65 | 6.35 |  |  |  |
| Depression | 2011-2019 | After Pregnancy | 34 | 6.17 | 5.26 | 7.07 |  |  |  |
| Depression | 2011-2019 | After Pregnancy | 35 | 5.60 | 4.74 | 6.47 | 0.86 | 0.78 | 0.94 |
| Depression | 2011-2019 | After Pregnancy | 36 | 6.11 | 5.20 | 7.01 |  |  |  |
| Depression | 2011-2019 | After Pregnancy | 37 | 5.89 | 5.00 | 6.79 |  |  |  |
| Depression | 2011-2019 | After Pregnancy | 38 | 5.87 | 4.98 | 6.76 |  |  |  |
| Depression | 2011-2019 | After Pregnancy | 39 | 5.41 | 4.55 | 6.27 |  |  |  |
| Depression | 2011-2019 | After Pregnancy | 40 | 5.52 | 4.65 | 6.39 | 0.87 | 0.79 | 0.96 |
| Depression | 2011-2019 | After Pregnancy | 41 | 5.69 | 4.80 | 6.57 |  |  |  |
| Depression | 2011-2019 | After Pregnancy | 42 | 5.43 | 4.56 | 6.30 |  |  |  |
| Depression | 2011-2019 | After Pregnancy | 43 | 5.87 | 4.96 | 6.77 |  |  |  |
| Depression | 2011-2019 | After Pregnancy | 44 | 5.31 | 4.45 | 6.17 |  |  |  |
| Depression | 2011-2019 | After Pregnancy | 45 | 5.77 | 4.87 | 6.67 | 0.89 | 0.81 | 0.98 |
| Depression | 2011-2019 | After Pregnancy | 46 | 5.18 | 4.32 | 6.04 |  |  |  |
| Depression | 2011-2019 | After Pregnancy | 47 | 4.72 | 3.90 | 5.53 |  |  |  |
| Depression | 2011-2019 | After Pregnancy | 48 | 5.84 | 4.93 | 6.76 |  |  |  |
| Depression | 2011-2019 | After Pregnancy | 49 | 5.60 | 4.71 | 6.50 |  |  |  |
| Depression | 2011-2019 | After Pregnancy | 50 | 5.00 | 4.15 | 5.85 | 0.92 | 0.79 | 1.08 |
| Depression | 2011-2019 | After Pregnancy | 51 | 5.43 | 4.60 | 6.27 |  |  |  |
| IR = incidence rate; IRR = incidence rate ratio; CI = confidence intervals; | | | | | | | | | |
| IRR adjusted for age and calendar year at delivery. week at follow-up. country of birth. region of residence. education. season at follow-up. age and calendar year at delivery. week at follow-up. country of birth. region of residence. education. season at follow-up. civil status. smoking. BMI category. multiple gestation. hypertensive disease. diabetes. and parity. | | | | | | | | | |

**Supplementary Figure 1**. Incidence rate ratio of psychiatric disorder subtypes during and after pregnancy compared with before pregnancy, by excluding those with a history of any other psychiatric disorders.


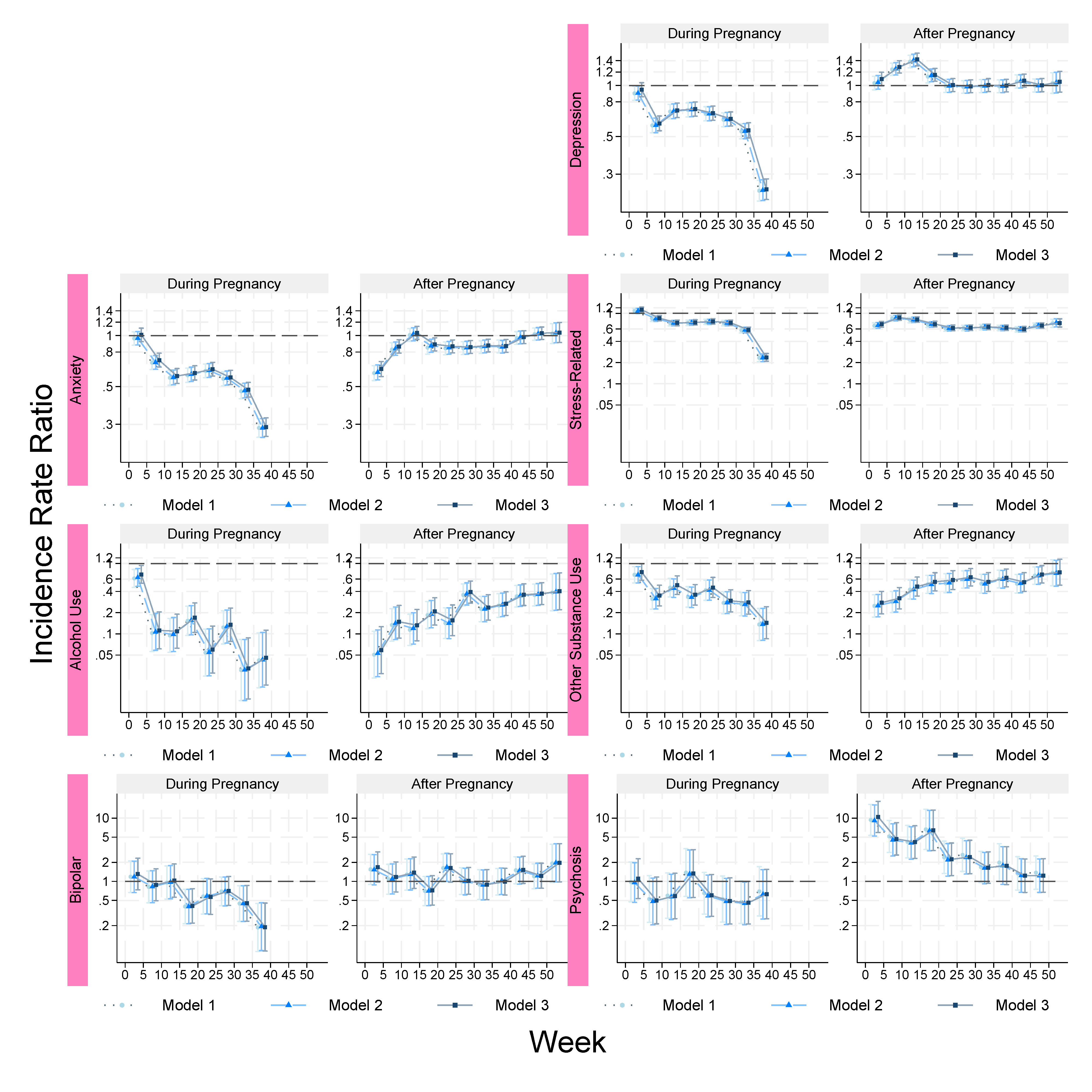


The incidence rate ratio was estimated by every 5 weeks. Model 1 was adjusted for age and calendar year at delivery and week at follow-up. Model 2 was additionally adjusted for country of birth, region of residence, education, and season at follow-up. Model 3 was additionally adjusted for civil status, smoking, BMI category, multiple gestation, hypertensive disease, diabetes, and parity.

**Supplementary Figure 2.** Standardized incidence rate of psychiatric disorders before, during, and after pregnancy by week, by restricting to three most populated counties where both primary care and specialist care data are available.


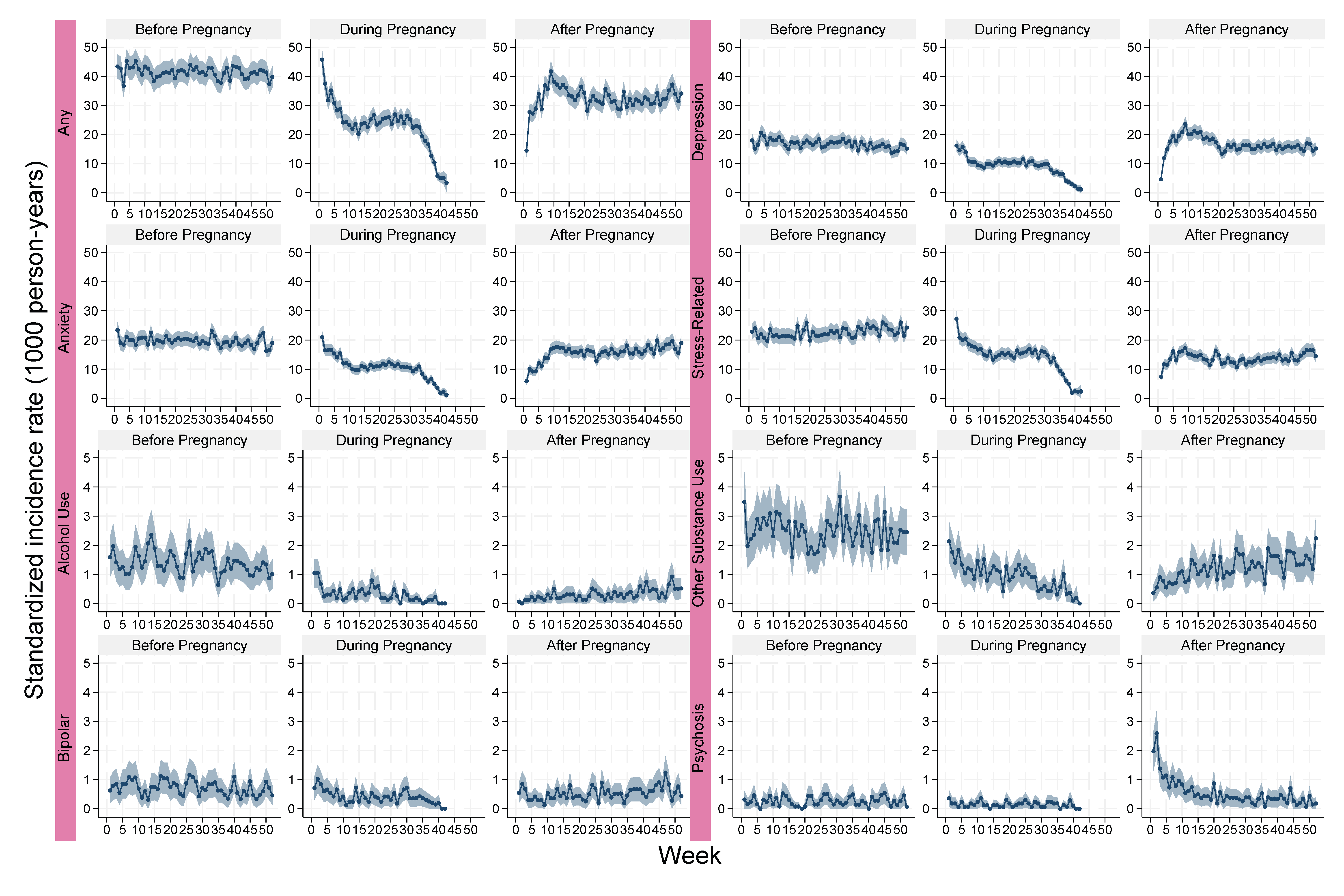


The incidence rate was standardized by age and calendar year at delivery.

**Supplementary Figure 3**. Incidence rate ratio of psychiatric disorders during and after pregnancy compared with before pregnancy, by restricting to three most populated counties where both primary care and specialist care data are available.


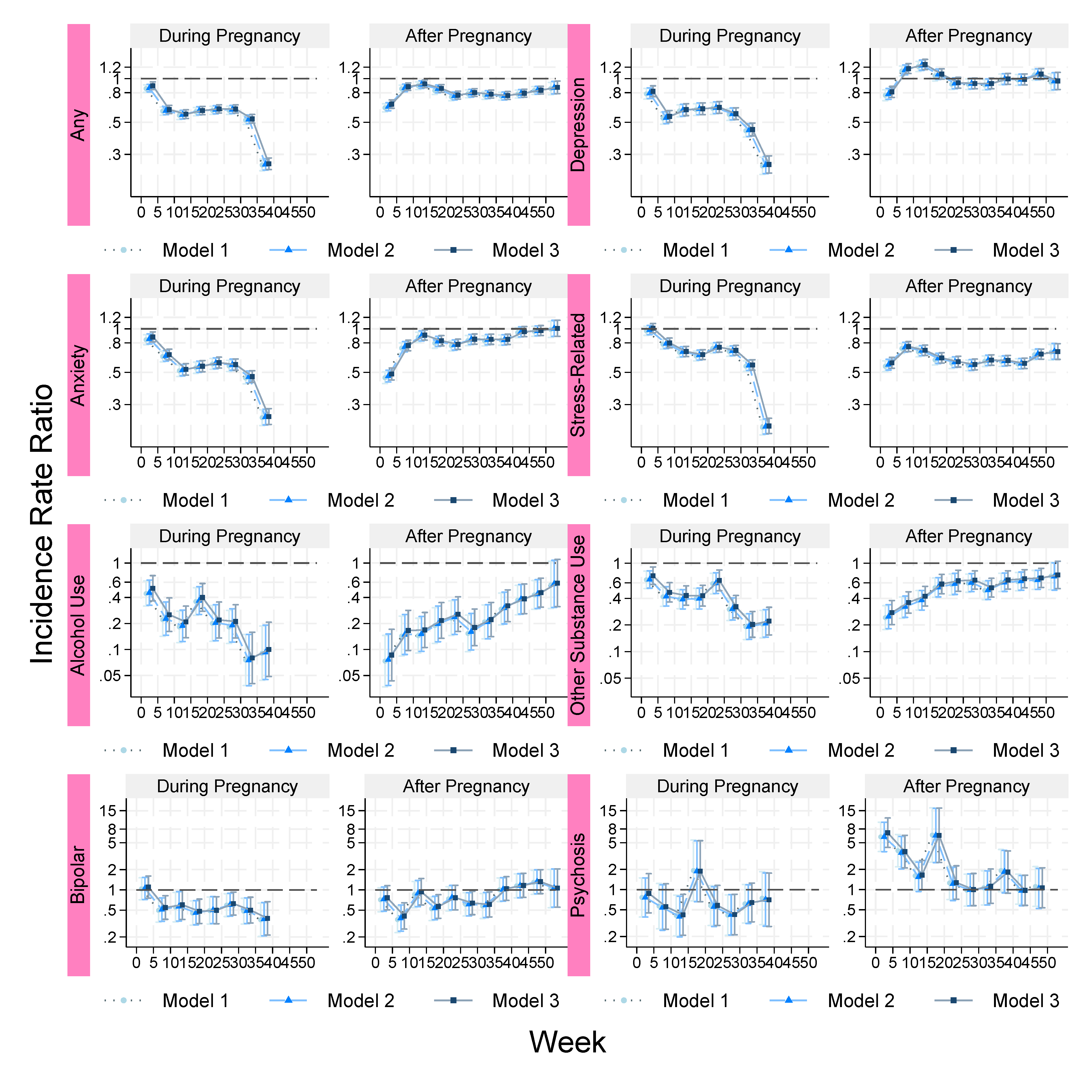
The incidence rate ratio was estimated by every 5 weeks. Model 1 was adjusted for age and calendar year at delivery and week at follow-up. Model 2 was additionally adjusted for country of birth, region of residence, education, and season at follow-up. Model 3 was additionally adjusted for civil status, smoking, BMI category, multiple gestation, hypertensive disease, diabetes, and parity.

**Supplementary Figure 4**. Standardized incidence rate and incidence rate ratio of any psychiatric disorder, and depression, stratified on educational level.


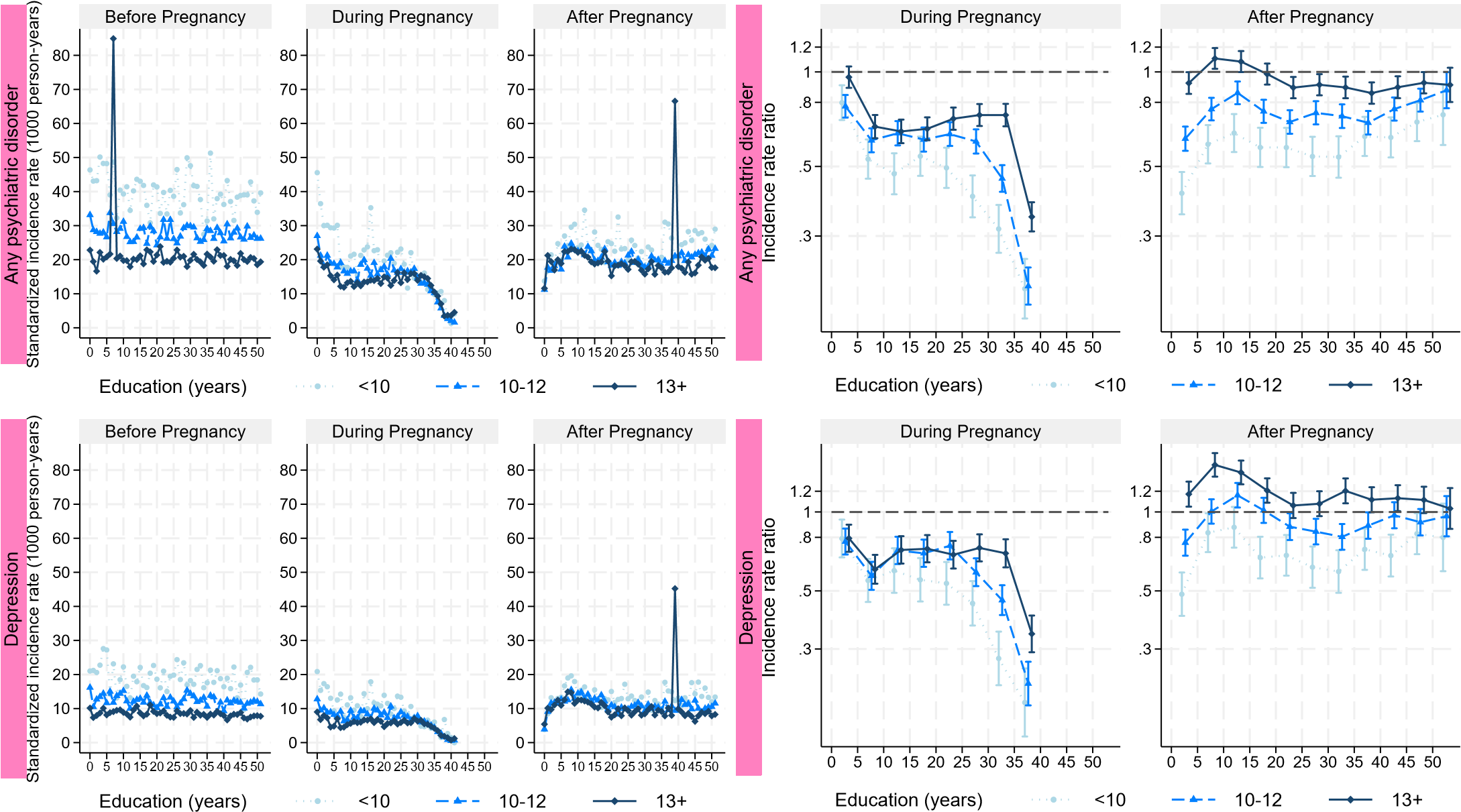


Week

The incidence rate was standardized by age and calendar year at delivery. The incidence rate ratio was estimated by every 5 weeks and adjusted for age and calendar year at delivery, week at follow-up, country of birth, region of residence, season at follow-up, civil status, smoking, BMI category, multiple gestation, hypertensive disease, diabetes, and parity.

**Supplementary Figure 5.** Standardized incidence rate and incidence rate ratio of any psychiatric disorder, and depression, stratified on parity.


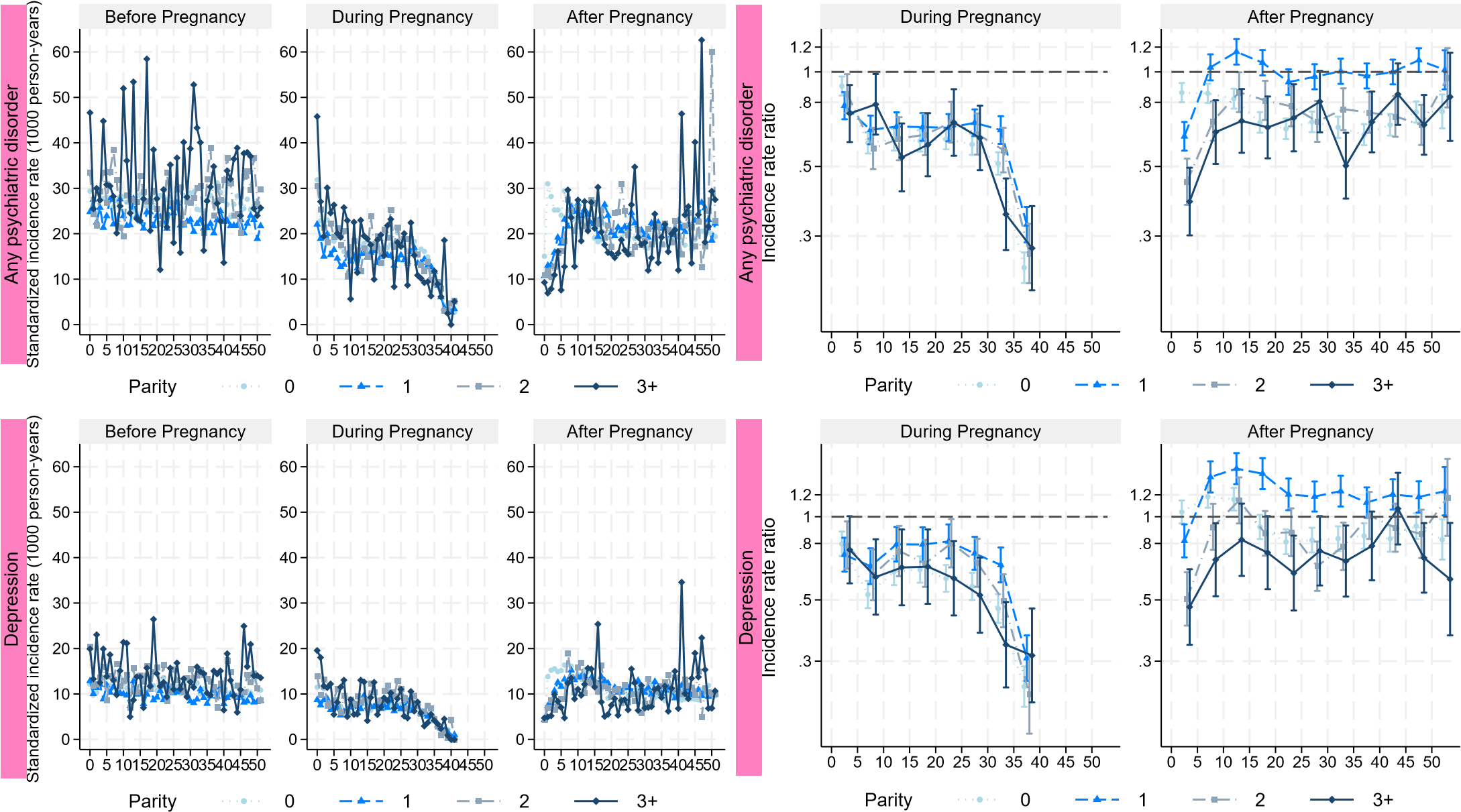


Week

The incidence rate was standardized by age and calendar year at delivery. The incidence rate ratio was estimated by every 5 weeks and adjusted for age and calendar year at delivery, week at follow-up, country of birth, region of residence, education, season at follow-up, civil status, smoking, BMI category, multiple gestation, hypertensive disease, and diabetes.

**Supplementary Figure 6.** Standardized incidence rate and incidence rate ratio of any psychiatric disorder, and depression, stratified on country of birth.


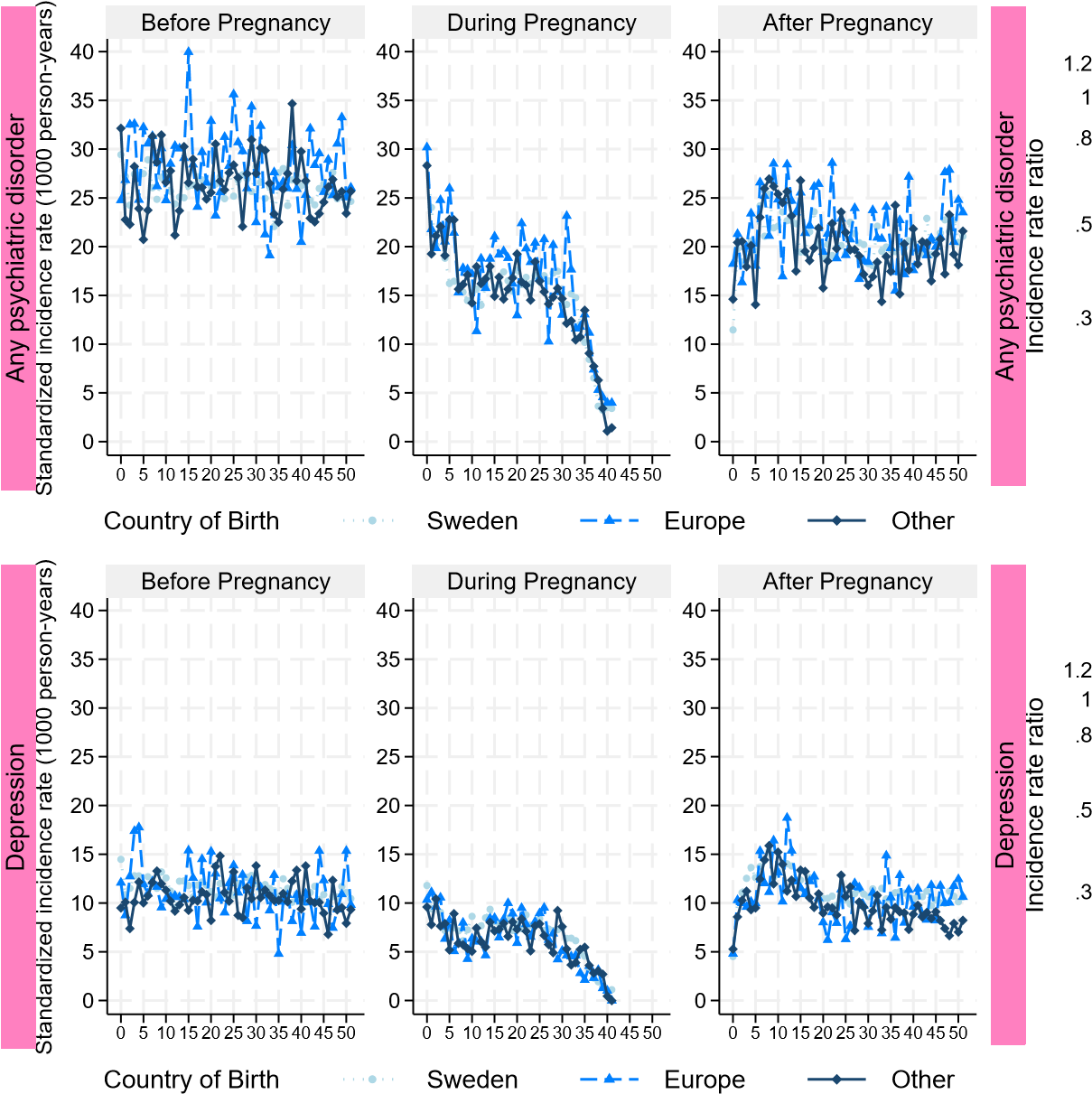

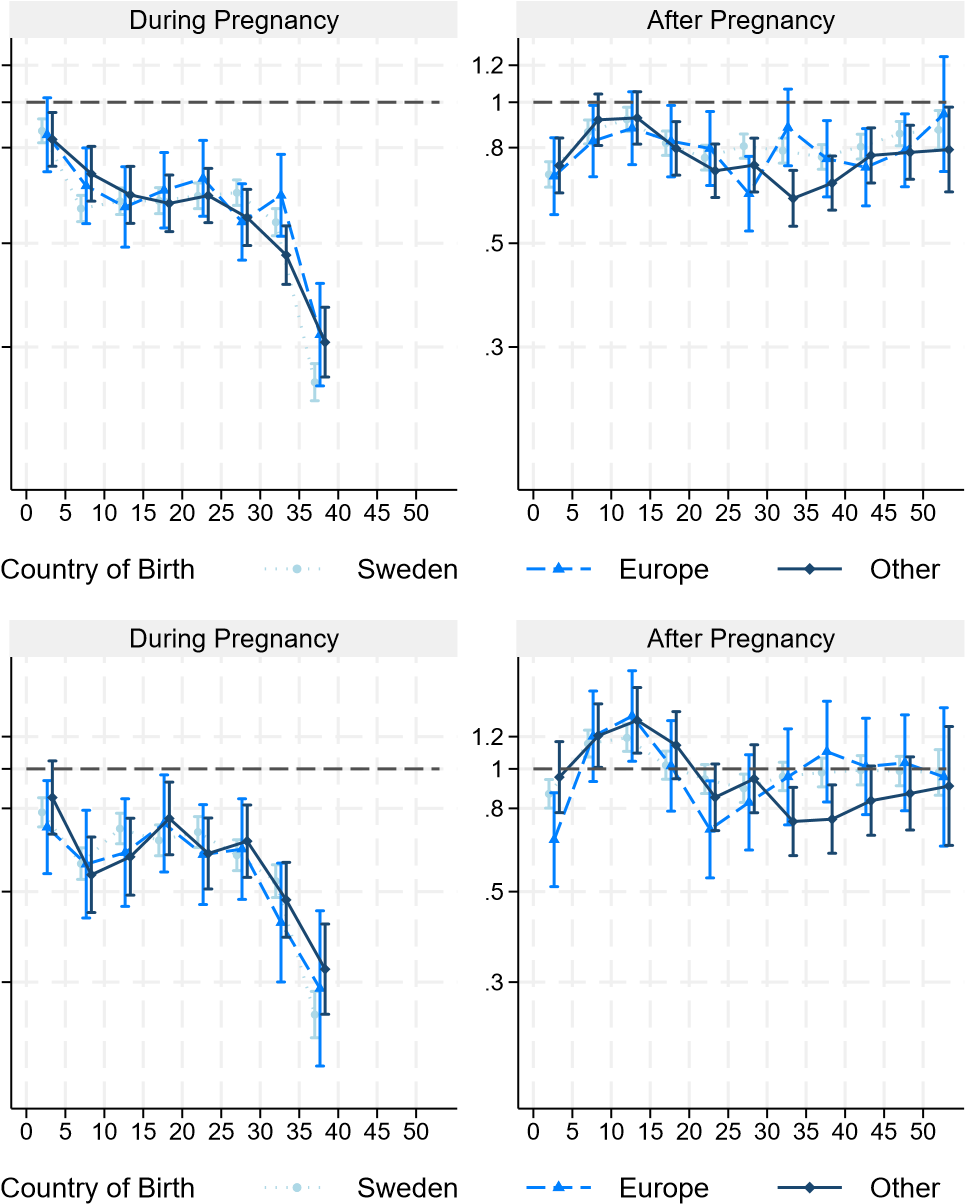


Week

The incidence rate was standardized by age and calendar year at delivery. The incidence rate ratio was estimated by every 5 weeks and adjusted for age and calendar year at delivery, week at follow-up, region of residence, education, season at follow-up, civil status, smoking, BMI category, multiple gestation, hypertensive disease, diabetes, and parity.
